# Drivers of spatio-temporal population dynamics of game species in a mountain landscape

**DOI:** 10.1038/s41598-024-53019-x

**Published:** 2024-02-01

**Authors:** Erich Tasser, Birgith Unterthurner, Andreas Agreiter, Lothar Gerstgrasser, Marco Giardino, Ulrike Tappeiner, Janette Walde, Johannes Rüdisser

**Affiliations:** 1 Eurac Research, Institute of Alpine Environment, Drususallee 1, 39100 Bozen/Bolzano, South Tyrol Italy; 2 South Tyrolian Hunting Association, Bozen/Bolzano, Italy; 3 Office for Hunting and Fisheries, Autonomous Province of Bozen/Bolzano, Italy; 4 Department of Ecology, Universität Innsbruck, Innsbruck, Austria; 5 Department of Statistics, Universität Innsbruck, Innsbruck, Austria

**Keywords:** Biodiversity, Community ecology, Forest ecology, Population dynamics, Urban ecology, Ecology

## Abstract

Since the end of the nineteenth century, socio-economic changes have greatly altered the Central European landscape and the structural and functional quality of habitats. Urban sprawl areas have appeared, a reduction of multiple forest uses has resulted in the densification of forests and agricultural land use has changed fundamentally through specialisation and intensification. Many of these changes affect biodiversity. To determine the important drivers of spatio-temporal dynamics of the population of 28 game species, we first considered a total of 130 potential explanatory variables. Second, we aggregated the main drivers of single-species models for habitat guilds. Third, we evaluated the results to aid in the development and implementation of mitigation measures for different ecoregions. We used harvest data as a surrogate for population density from 1875 to 2014 in South Tyrol, Italy. In generalised linear models, we used environmental characteristics such as climate, landscape diversity and structures, land cover, hunting, wildlife diseases, competition and predation, land-use type, and intensity (including pesticide use) as explanatory variables to predict the spatio-temporal dynamics of game species. The important drivers are land use and management changes (intensification in the agriculturally favourable areas, extensification or abandonment in the unfavourable areas) as well as associated changes in the landscape features, diversity and structure, and hunting management. Climatic variables, interspecific competition and diseases only play a subordinate role. The dynamics of the habitat guilds and their drivers provide concrete indications for measures to maintain or improve the habitat quality for the investigated species. Particularly important are transfer payments to ensure extensive agricultural use, increasingly through the takeover of personnel costs, but also for the installation of an independent body that monitors and evaluates the effectiveness of the measures.

## Introduction

Social and economic developments, the integrity of ecosystems and, thus, biodiversity and the quality of human life are interlinked and ultimately interdependent. Such complex social-ecological systems can adapt to new conditions to a certain extent but are limited by spatial or functional boundaries^1^. However, balancing all major drivers and beneficiaries is the most challenging problem in natural resource management. Only if the entire system remains resilient can it last for a long time^2^. Thereby, social-ecological resilience includes permanent adaptability and transformability in the context of trends, shocks and seasonality, which can appear as natural disturbances (e.g. weather-related disasters) or as anthropogenic disturbances (e.g. human–environmental mismanagement)^3^. It is important to have integrative variables to be able to recognise the responses of the social-ecological system to economic development, especially when there are misdevelopments^2^.

Because quantitative biodiversity data (e.g. species abundance) are rarely available for historic time series^4^, researchers have proposed using harvest data from hunting as variables for population abundance. The suitability of such harvest data to analyse long-term population dynamics has been demonstrated in studies with ungulates^5,6^, carnivores^7^, lagomorphs^8,9^ and birds^10,11^. These studies have also shown that many different drivers influence the dynamics of game species. There are known effects through agricultural and forestry use practices^5,6,10^; natural and anthropogenic habitat changes (e.g. fragmentation or isolation)^7^; climate and weather effects^5,8,9^; and influences through hunting, predation and interspecific competition^5,7^. However, these effects do not always occur everywhere and simultaneously in space because specific landscape quality changes are the results of socio-economic fluctuations and natural processes, which can vary significantly depending on the environmental conditions in the space. Especially in mountainous regions, there are different situations within a very small area as a consequence of high variability in site conditions (biotic and abiotic), land use and urban sprawl^12^. According to the biology of game species, certain species only occur in specific habitats, whereas others occur almost everywhere. Because populations are subject to natural and artificial fluctuations and different species are also influenced by different drivers, considering a single species only provides partial information about the overall status of a habitat. Therefore, various authors have combined several species to better reflect long-term dynamics^13^. Composite trend indicators are an approach where a group of population trends are used to describe environmental changes and reflect the population responses^14^. Some of them, such as the Wild Bird Index, are adopted by the EU and incorporated in the Streamlining European Biodiversity Indicators (SEBI) set to address the EU biodiversity targets^15^. For example, 17 or 28 forest species were used to analyse the ecological status of forests and calculate the European Union (EU) Forest Bird Index^16,17^, and 33 common bird species associated with agricultural land were combined for the EU Farmland Bird Index^18^. Hence, in this study we used habitat guilds to determine key drivers in each habitat and to make the broadest possible statement about habitat condition.

Our study is based on the assumption that the abundance dynamics of game species is driven mainly by changes in habitat or landscape quality^19,20^. These, in turn, are influenced by human land use. In the case of game species, of course, hunting management also plays a decisive role. Abiotic variables, such as climate and topography, as well as biotic variables, such as epidemics, predation and competition, play only a minor role^5^. Using the example of South Tyrol, a mountainous region in the Italian Alps, we have attempted to elicit the variables for the development of 28 game species—including habitat specialists and generalists. This framework has allowed us:

- to investigate the main drivers of the spatio-temporal dynamics of the 28 game species in South Tyrol by using single species models;
- to compile the main drivers for habitat guilds, associated ecoregions, and landscapes; and
- to evaluate and communicate the effects of landscape changes to aid the development and implementation of necessary mitigation measures.

## Results

### Drivers for game species dynamics

**Figure 1:**
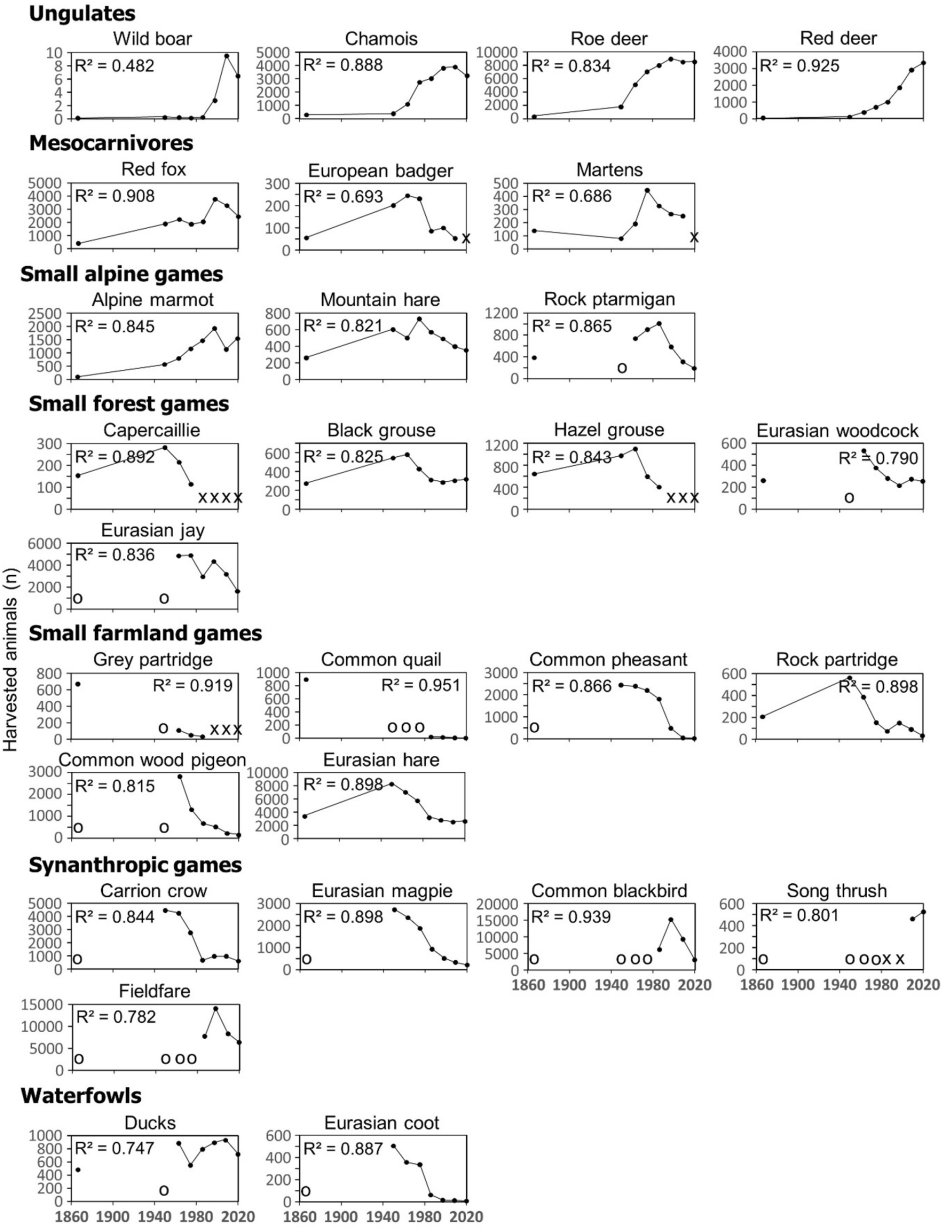
Dynamics of harvests of the game species in South Tyrol since the end of the nineteenth century grouped into guilds according to habitat preferences. The pseudo-R^2^ (in the plots just R^2^) of the single species models are given. o = no data, x = protected. Species: wild boar (*Sus scrofa),* chamois (*Rupicapra rupicapra),* roe deer (*Capreolus capreolus)*, red deer (*Cervus elaphus)*, red fox (*Vulpes vulpes),* Eurasian badger (*Meles meles), martens* (*Martes foina*, *Martes martes*), Alpine marmot (*Marmota marmota*), mountain hare (*Lepus timidus*), rock ptarmigan (*Lagopus muta*), capercaillie (*Tetrao urogallus*), black grouse (*Lyrurus tetrix*), hazel grouse (*Tetrastes bonasia*), Eurasian woodcock (*Scolopax rusticola*), Eurasian jay (*Garrulus glandarius*), grey partridge (*Perdix perdix*), common quail (*Coturnix coturnix*), common pheasant (*Phasianus colchicus*), rock partridge (*Alectorix graeca*), common wood pigeon (*Columba palumbus*), Eurasian hare (*Lepus europaeus*), carrion crow (*Corvus corone*), Eurasian magpie (*Pica pica*), common blackbird (*Turdus merula*), song thrush (*Turdus philomelos*), fieldfare (*Turdus pilaris*), ducks (*Anas platyrhynchos, Anas querquedula* and* Anas crecca*) and Eurasian coot (*Fulica atra*).

The explanatory power of the generalized linear models (GLM) varied significantly among the 28 game species (Fig. 1). The pseudo coefficients of determination (pseudo-R^2^) at the link level were lowest for the wild boar (pseudo-R^2^ = 0.482), martens (pseudo-R^2^ = 0.686) and European badger (pseudo-R^2^ = 0.693) and highest for the common quail (pseudo-R^2^ = 0.951), common blackbird (pseudo-R^2^ = 0.939) and red deer (pseudo-R^2^ = 0.925). The median pseudo-R^2^ was 0.861 for ungulates with increasing abundance, 0.693 for mesocarnivores, 0.845 for alpine game species and 0.836 for forest game species. Habitat guilds with a decreasing dynamic yielded higher pseudo-R^2^ values: for farmland game species, the median pseudo-R^2^ was 0.898 and for synanthropic species, it was 0.869. The dynamics of the waterfowl could also be explained appropriately with a median pseudo-R^2^ of 0.817.

**Figure 2:**
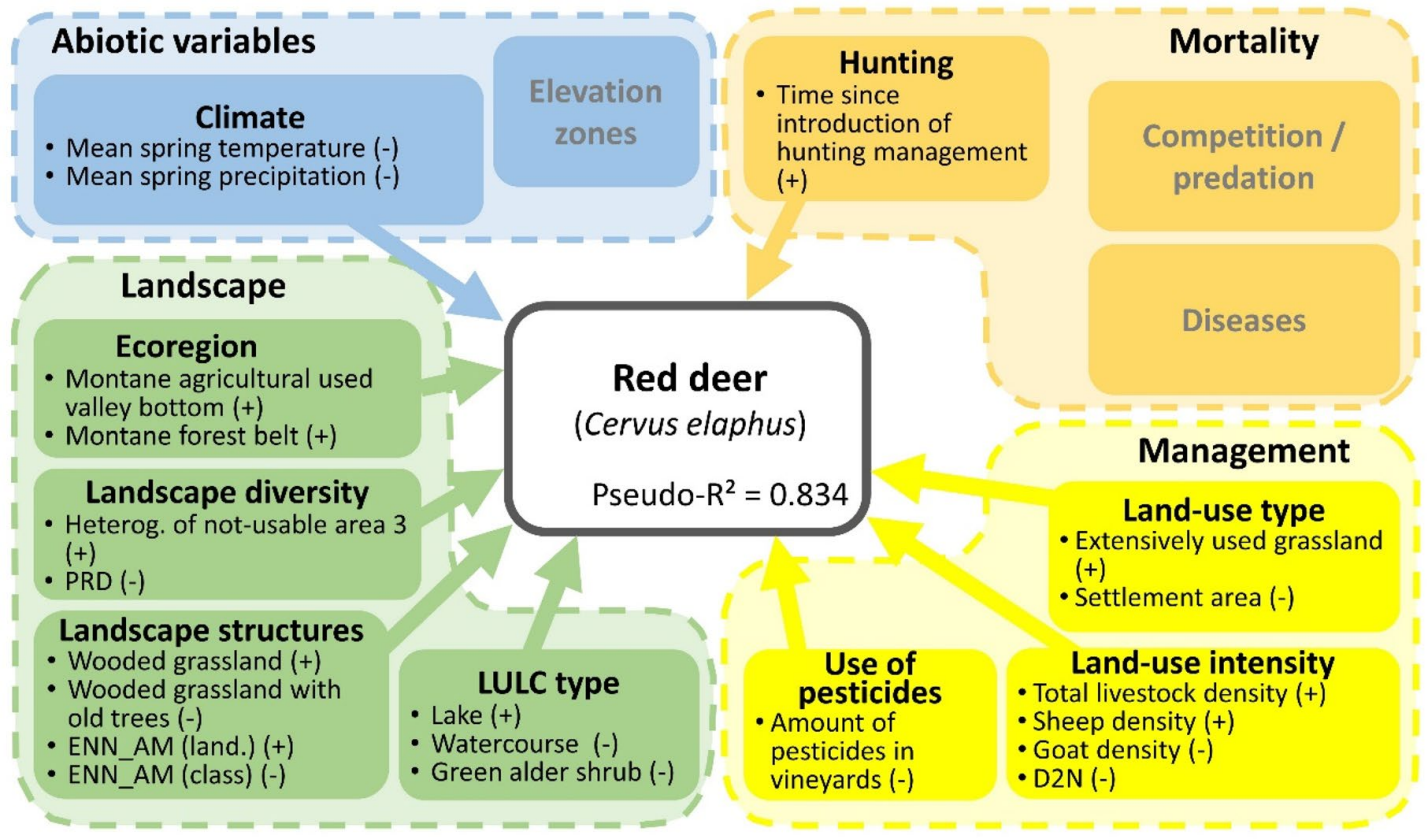
The results of the single species generalised linear model for red deer, modelling the relationship between the abundance of harvested red deer and the environmental and land-use variables. Only the significant correlation coefficients and their signs (in parentheses) are shown (for coefficient estimates, see Appendix E). LULC: land use/land cover; PRD: patch richness density; ENN_AM (land.): Euclidean mean nearest neighbor distance, landscape level; ENN_AM (class): Euclidean mean nearest neighbor distance, class level; D2N: distance to nature.

We used the example of explaining the spatio-temporal harvest of red deer to describe the findings obtained from the GLM and to facilitate the interpretation of drivers (Fig. 1, for details Appendix C). This species has a dynamic population development (Fig. 2, for details Appendix C). Landscape characteristics had the greatest effects on abundance. There were positive relationships between abundance and the proportion of agriculturally used valley bottom (montane) as well as the proportion of montane forest area. A high proportion of wooded grasslands with young trees and lakes as well as high variability of areas that could not be used for agriculture (natural areas) had a positive effect. On the other hand, an increase in the patch richness density (PRD) and a decrease in the mean nearest neighbour distance (ENN_AM) at the landscape level—both of which indicate a small-scale, fragmented landscape—hurt the harvest. In addition, there were negative correlations with three land-use/land cover (LULC) types. First, the proportion of lakes had a positive effect on abundance, green alders (which correlated with the proportion of subalpine forest areas) and large streams in the lower-lying valley floors but a negative effect on abundance. Furthermore, the harvest quota of red deer was negatively influenced by the warm and humid spring months. From the land-use perspective, a proportion of grasslands—in this case, measured by high livestock density (total livestock density and sheep density) and by the proportion of extensively used grasslands in the hunting ground area—increased deer abundance. In contrast, the expansion of anthropogenically changed habitats such as settlement areas and permanent cultures (measured by the high amounts of pesticides and the distance to nature (D2N)) hurt deer abundance. Finally, the introduction of hunting management in South Tyrol, with strict controls, had a highly positive effect on the number of red deer harvested.

### Drivers of habitat guild dynamics

**Figure 3:**
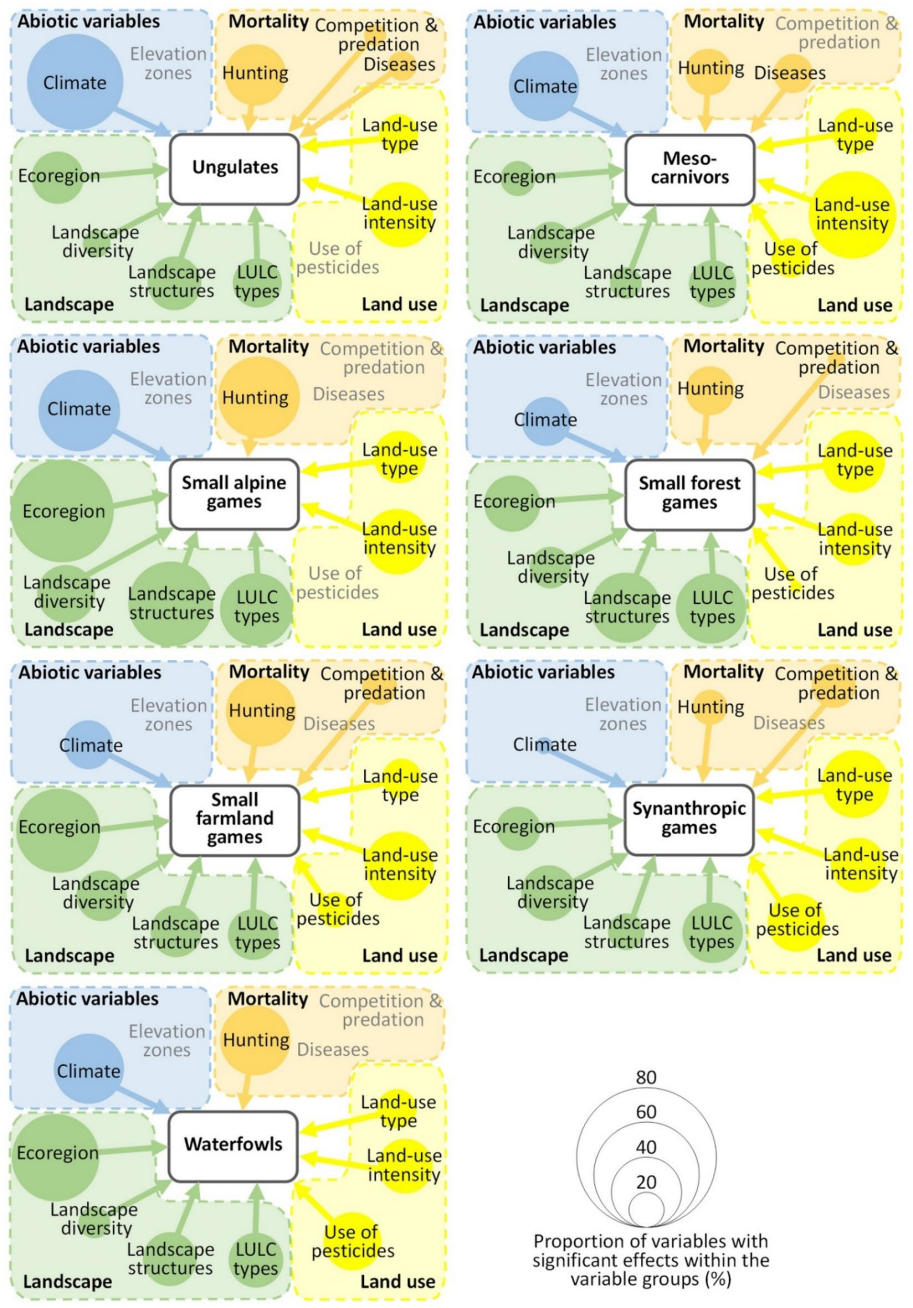
The results of the generalised linear models to detect correlations between the abundance of harvested individuals per habitat guild and the explanatory variables (drivers). The size of the circles reflects the number of significant correlations between the single variables within a variable group and the harvest abundance relative to the total number of potential correlations (based on the number of variables within the group multiplied by the number of species per guild). For information on the game species contained in each habitat guild, see Fig. 1. There were no correlations for the variable groups written in grey without an arrow. The significant correlation coefficients of the explanatory variables on the single species can be found in Tables E1–E4 in Appendix E, and correlations between the explanatory variables are presented in Appendix D. LULC: land use/land cover.

The dynamics of the abundance of game species with the same habitat preferences correlated with different variables or variable groups, providing indications for the significant drivers of spatio-temporal changes in the different ecoregions/landscapes (Fig. 3).

#### Ungulates

For ungulate species with an increasing population (Fig. 1), climate, hunting restriction and land-use intensity were of great importance (Fig. 3, Table E1 in Appendix E). Precipitation correlated positively and temperature correlated negatively with all ungulates, and an increase in land-use intensity (change in permanent cultures or vineyards or increasing livestock density) correlated negatively. In addition, hunting restrictions had a positive effect on population trends over time. There were also minor effects from epidemics and competition. Compared with the other habitat guilds, landscape features played a subordinate role for the ungulates. The same drivers were also important for mesocarnivores (Fig. 3, Table E1). The abundance of these species correlated, above all, with warm years with little rainfall. Furthermore, greater agricultural land-use intensity with more livestock as well as hunting regulation corresponded positively. The mesocarnivores were also affected by epidemics and diseases, whereby the harvests correlated positively with them. This can be explained by the fact that in South Tyrol when rabies, mange and distemper occurred, massive countermeasures were taken through intensive hunting and a significant reduction in the populations. In addition, vaccination campaigns were carried out.

#### Small alpine game species (Fig. 3, Table E2)

Alpine marmot, mountain hare and rock ptarmigan occurred primarily in the subalpine to alpine belt. Only the mountain hare also had its habitat in the montane slope belt (a positive correlation with the proportion of montane slope area). Regarding climate conditions, warm temperatures had a negative effect on the mountain hare population, and all species benefited from high precipitation. These species also had higher-than-average interactions with landscape characteristics. High Shannon Diversity Index (SHDI) and Patch Richness Density (PRD) values correlated positively with harvest abundance, as did high structural diversity and a high proportion of wetland habitats in the area. Furthermore, there were positive interactions with the proportion of richly structured but forest-free areas in the hunting ground. A high proportion of grassland use and a high sheep density, indicating extensive agricultural use, had led to positive dynamics until the 1970s/1980s. Since then, however, they have again decreased significantly, and these declines correlated with the expansion of forest area and intensively used agricultural land.

#### Small forest game species (Fig. 3, Table E2)

These five species showed similar harvest dynamics as the alpine species (an initial increase in harvests was followed by a decrease). The species correlated positively with a high proportion of montane forest areas and slopes in the hunting ground. A high SHDI and PRD correlated positively with abundance, whereas an increase in the abandonment of agro-forestry systems (e.g. larch meadows) correlated negatively with it. In contrast, there were differences among species concerning habitats. In general, the expansion of dense forests had a negative effect on subalpine areas and uncultivated agricultural land, whereas wetlands had a positive effect. Finally, significant correlations with variables from the hunting management group occurred frequently. Hunting ground size correlated with abundance, as did hunter densities and hunting protection status.

#### Small farmland species (Fig. 3, Table E3 in Appendix E)

These species showed a strong decrease in abundance or even disappeared completely (e.g. partridge and quail). The population sizes of these species hardly interacted with the climate conditions, but even more so with the presence of certain land-use types and high landscape diversity. The percentages of hay meadows, arable land and vineyards, as well as high landscape structuring and SDHI, correlated positively with harvest abundance. However, many of these species also correlated negatively with orchard expansion, land-use intensification or increased pesticide use. As in the case of the farmland game species, the abundance of the synanthropic game species (Fig. 3, Table E3) also decreased. This was mainly due to the removal of hedge structures, the clearing of the agricultural landscape and a decrease in arable land. On the other hand, the increase in the intensity of grassland use and the expansion of vineyards and settlement areas correlated especially positively with thrush species abundance. In contrast, the expansion of apple orchards (including pesticide use and hail nets) correlated negatively with raven and magpie harvest abundances. There were some correlations with predators and interspecific competition for synanthropic game and farmland species.

#### Waterfowl (Fig. 3, Table E4)

Finally, there was no consistent population trend in waterfowl. Common drivers were winter precipitation, montane belt, edge density and a decrease in forest. Winter precipitation, a high reforestation and edge density correlated negatively and greater grassland use in montane agrarian landscapes and more settlements positively with the abundances of both species.

## Discussion

### Population dynamics and landscape development

Population dynamics of wildlife species reflect changes in the availability of key resources, such as food, refuges and nesting sites^10,21^, and these factors are highly connected with changes in land use and management due to social-economic development^13,16,22^. Conversely, changes in species abundance or habitat guild abundance can provide relevant information regarding positive or negative trends in habitat and landscape quality, even if these landscape developments do not appear to be immediately visible. We took advantage of this fact by interpreting the development of single species or habitat guilds to derive important drivers for habitat quality (see also^19,20^). This is especially possible when models of density or viability are used in combination with spatial habitat information (such as the Habitat Suitability Index (HSI))^23^. Accordingly, statements on habitat and landscape qualities and their changes can be derived from population trends^19,24^—not only in space but also in time^20^.

While ubiquist species can adapt easier to changing landscapes, smaller, specialised game species are particularly sensitive to changes in their habitat. Thus, their population development reflects the consequences of land use and management decisions^22,25–27^.

### Drivers of the spatio-temporal dynamics of specialists

#### Small alpine game species

The harvest abundance of all alpine game species showed an increase until the 1970/80s; subsequently, the mountain hare and rock ptarmigan populations decreased substantially, whereas the marmot population decreased only slightly. At the global level, the mountain hare is classified as a species of least concern, with an unknown population trend^28^. In some parts of the Alps, such as France and Carinthia (Austria), the populations are decreasing or are even near threatened^29^. In our case, the abundance of the mountain hare correlated mainly with some climate conditions, the proportion of forest-free but richly structured landscapes and the naturalness of the landscape as a whole. Other authors have documented similar relationships^26,30,31^. The rock ptarmigan is even more sensitive to climate and land-use changes as well as the recreational use of alpine terrain. Delayed snowfall in autumn had an especially negative effect, possibly due to a mismatch in time to moult to the white winter plumage, which increased the predation risk^32^. Furrer et al.^33^ also proved that an upward shift of the treeline and a local increase in winter and/or summer tourism have a negative effect. We also noted sensitivity concerning climate and land use in our results; however, we did not find a negative correlation with tourism intensity.

In our study, the Alpine marmot abundance correlated with the proportion of alpine pastures, with a higher mowing frequency and sheep density. Furthermore, we noted a greater abundance in areas with higher tourism intensity, which in turn could indicate increased use of the alpine pastures in these areas. Other researchers have come to similar conclusions. According to^34^, marmots prefer especially anthropogenic sites (meadows, summer pastures and dry stone walls), where their reproduction rate was highest. Thus, marmots compete with farmers for space^35^. As a result of the decrease in summer pastures in South Tyrol^12^, the marmot is losing habitats, a phenomenon that explains the slight decrease in population densities. In summary, the alpine ecoregion in South Tyrol has been losing habitat quality since the middle of the last century due to a decrease in areas used for agriculture and the accompanying reforestation.

#### Small forest game species

In South Tyrol, the typical forest grouse species (capercaillie, black grouse and hazel grouse) and the Eurasian woodcock showed similar abundance dynamics. After the nineteenth century, their abundances increased significantly until the middle of the twentieth century and then slowly decreased again until the end of the twentieth century, when they stabilised at a low level. These species have profited from rigorous hunting management, but they have subsequently suffered because of modern forestry and agricultural activities. In South Tyrol, as in many Alpine countries, since 1950 the forests in the interior have become much denser due to the marginalisation of forestry and the separation of forest and pasture^12,36,37^. As a result, the understorey vegetation has been reduced significantly, whereby the breeding success and the survival of these species depend, above all, on the presence of insects (in the chick phase) as well as bilberry and cowberry^38^. Therefore, open forests and semi-open areas with a high density of young trees have a positive effect on these species, whereas grazing by goats has a negative effect. In summary, forest quality in terms of habitat for forest birds has improved over the past decades, initially through the extensification of forest use, but has slowly deteriorated due to increasing densification.

#### Small farmland game species

The farmland game species in South Tyrol have declined sharply, and some of them have even disappeared from the landscape. As our results show, this phenomenon correlates mainly with changes in the use of agricultural land. On the one hand, almost no arable land is being cultivated anymore and, on the other hand, many structures (e.g. hedges and stone walls) have been removed, wetlands have been drained, and land use has been intensified^12^. Traditional orchard meadows have given way to modern orchards, where highly effective pesticides are increasingly used and hail nets have been installed. As shown in other studies from Europe^10,22,27^, it is precisely these developments that are responsible for a reduced supply of key resources, such as food and nest sites, for many of these species. Therefore, in South Tyrol, the agricultural area as habitat for these farmland species has qualitatively deteriorated since the middle of the last century.

#### Synanthropic game species

As mentioned elsewhere^39^, synanthropic species are mainly granivorous, aerial insectivorous, ground foragers, cavity nesters, sedentary birds, social birds and species with a wide latitudinal and altitudinal tolerance as well as exotic species. The synanthropic game species in our work are also mainly granivores, ground foragers and sedentary birds and have a wide latitudinal and altitudinal tolerance. Accordingly, they have their highest densities not only in the settlement and urban areas but also in the environment used for agriculture^25^. As with the farmland species, the abundance of synanthropic species has decreased markedly in South Tyrol. As with farmland species, this is due to the decrease in the necessary resources (food and nesting sites) outside of urban areas. In the urban areas themselves, however, the population trends are likely to be different. In the cities of Central Europe, urbanisation has increased densities and biomass but also reduced species richness^40^. We could show a direct connection with settlement development for the thrush species.

#### Waterfowl

Ducks (in our case, especially mallard ducks) showed an increasing trend. Ducks were hunted intensively in the nineteenth century, which decimated the population. However, due to the high potential reproduction, the populations recovered rapidly in the middle of the twentieth century and stabilised at a high level. In principle, areas in which open land is closely interlinked with water bodies are suitable duck habitats^21^. Ducks seek their food both on land and in water, but they nest on land. According to our results, these species benefit from highly structured agrarian habitats but also from an intensification of grassland farming and the associated increase in the frequency of cutting, which makes it easier to find food on land^41^. The development of the Eurasian coot is different. The population of this species has decreased significantly since 1980, a phenomenon that has also been observed in other regions of Europe^42^. Coot populations are sensitive to food resources, especially submerged macrophytes and aquatic insects, and compete with fish^43^. In sum, the population trends of ducks and Eurasian coot indicate that the quantity of habitat in South Tyrol has not changed, but the quality of existing wetlands (especially of eutrophic wetlands) may have decreased. This is an alarming sign because during the last century, more than half of the wetlands throughout the world were lost because of anthropogenic actions^44^.

### Drivers of the spatio-temporal dynamics of ubiquist species

Ungulates and mesocarnivores inhabit forests or a mosaic of forests and open land over a wide habitat and elevation spectrum. In our study, all ungulate species showed increasing population dynamics. The population of roe deer and chamois have stabilised at high levels in recent decades; the other two species are still in the process of dispersal in South Tyrol. The ungulates were all severely decimated or, in the case of the red deer and wild boar, exterminated in the nineteenth century by habitat loss and the intense human hunting pressure in South Tyrol^45^. These species could recover again through regulated hunting management. Moreover, we showed that these species benefit, above all, from the expansion of forest areas and natural alpine grasslands as a result of the abandonment of sites that are unfavourable for agriculture, and in the case of red deer and wild boar also from the intensification of agricultural use in favourable locations^12,46^. A reduction in direct food competition from small ruminants (sheep and goats) in forest and subalpine and alpine summer pastures also has a positive effect, a finding consistent with previous studies^47,48^.

Like the ungulates, the mesocarnivores have also benefited from a reduction in hunting pressure and a large and diverse landscape of forests and agricultural land. The three dominant species in the area (red fox, European badger and stone marten) feed in highly varied ways. Besides insects, worms, small mammals, birds, wild fruit and carrion, they also benefit from cultivated fruit^49^. Thus, the development of land use in South Tyrol does not directly explain the decreasing hunting harvest of these species since the 1980s. However, since then, both grassland use and orchards and vineyards have intensified significantly, and arable land has largely disappeared^12^. Plant as well as faunistic diversity and abundance, and thus food supply, has decreased^50,51^. Furthermore, intensive pesticide use in particular hurts habitat quality^52^. In summary, the habitat for ungulates and mesocarnivores has improved qualitatively and quantitatively in South Tyrol through the abandonment of marginal land, increased reforestation, a reduction in the competition with small ruminants and separation of forest and pasture use in agroforestry systems. However, the intensification of agricultural land use over the past four decades has led to an overall reduction in the availability of food for mesocarnivores.

### Implications for landscape management and planning

Based on our interpretation of the developments of the habitat guilds in terms of the qualitative changes in the individual ecoregions, our results provide concrete indications of which drivers are responsible for the developments. Furthermore, measures can be derived that can be taken to maintain or improve the situation in the ecoregions.

- *Subalpine-alpine ecoregion* The future temperature rise will permit a shift in the timber line to higher elevations^37^, which will automatically reduce the natural habitat for alpine species^53^. Humans can counteract this development by using such areas as summer pastures or by artificially preventing reforestation. This approach requires direct payments for pasture management^54^ or agri-environmental measures^55^. However, in most regions, the current payments cannot compensate for the high labour costs and the low product prices^46^. Therefore, the level of payments would have to be adapted to ensure low-input grassland use. Otherwise, manual felling of trees and removal of shrubs could be done by trained personnel in a perennial rhythm. Of course, it must be examined whether such management can be an alternative, sustainable and permanent solution to preserve or create additional habitats for the target species. Furthermore, it must be clarified what public costs are involved and how these can be financed.
- *Forest ecoregion* Agroforestry systems (larch meadows and forest pastures) and semi-open forest stands are preferred habitats for many plant and animal species^51,56^. Some game species, such as capercaillie, black grouse, roe deer and red deer, also benefit from such forests^57^. On the other hand, these systems have negative consequences, especially in times of climate change, such as reduced carbon storage and ecosystem stability compared with a near-natural forest^56^. Therefore, from a social perspective, it is important to strive for a near-natural forest over as large an area as possible^58^. All measures that conflict with this goal (e.g. intensive and large-scale forest grazing) should be reduced to those areas whose preservation makes sense from a cultural-historical, hunting and nature conservation perspective.
- *Agriculturally used ecoregion in the colline and montane belt* Many farmland species are endangered or have disappeared, even though the EU has increased the emphasis on greening agriculture for the past 20 years^59,60^. Therefore, the EU is currently stepping up its efforts to counteract species extinction in the agricultural landscape with the Green Deal and the EU Biodiversity Strategy 2030^61,62^. In this sense, it is not a lack of legal targets but rather, as our results also show, a lack of implementation. Greening has not yet been noticeably implemented in the area. For example, the compensation areas are often lacking, the quality of such compensation areas is not defined, their management measures are missing, and the specifications for spatial distribution are missing. Furthermore, no specific management information and no data about pesticide use are available. From our point of view, it would also be particularly important to have an independent organisation that monitors and evaluates these measures.

## Material and methods

### Study area

**Figure 4:**
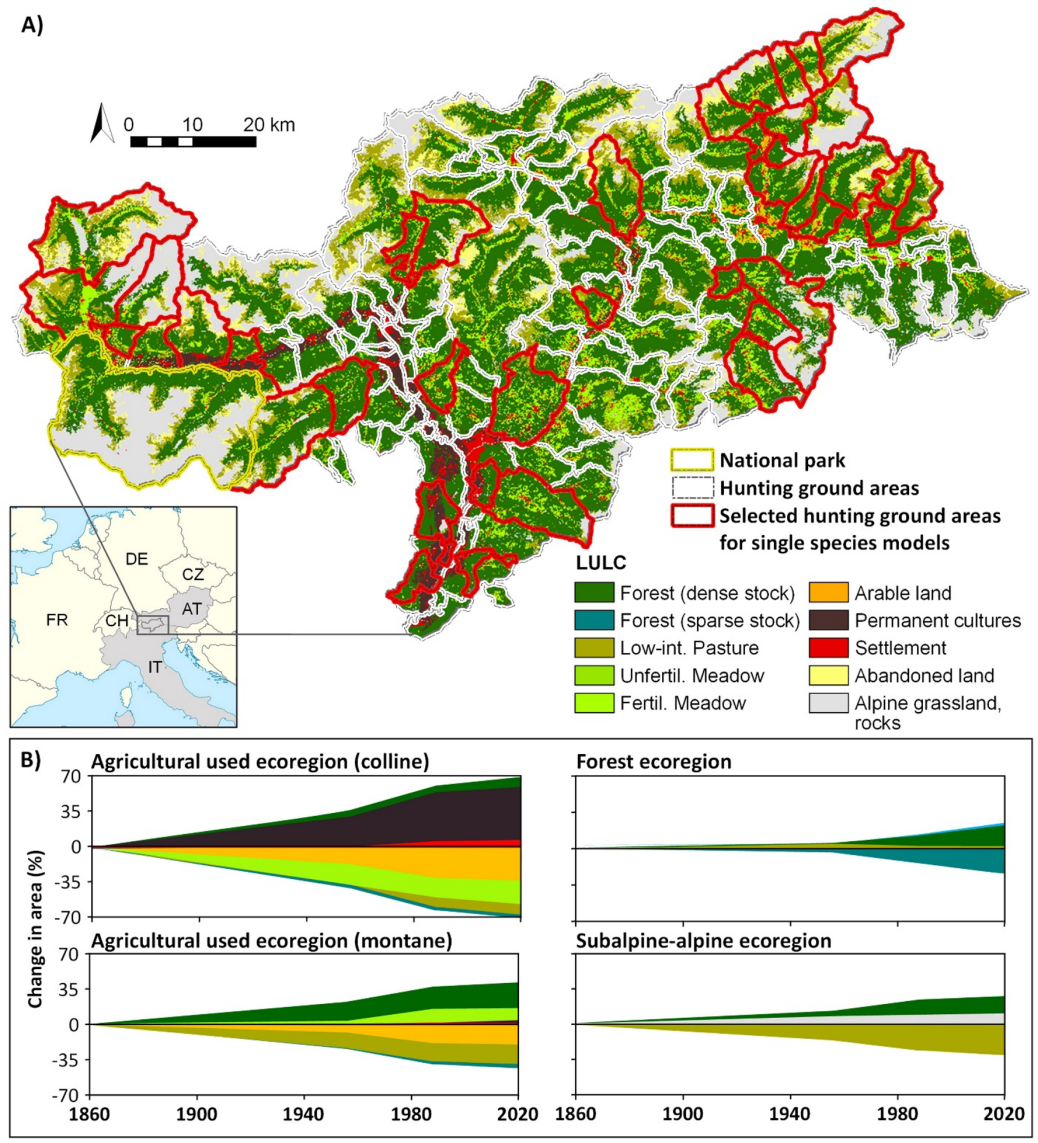
(**A**) The study area, indicating the current distribution of the main land use/land cover (LULC) types and hunting ground areas (map created with ArcGis Desktop v. 10.8; https://www.esri.com). (**B**) LULC changes since 1860 for the most important ecoregions (adapted from^64^). The graphs show the accumulative increase and decrease for each LULC type using 1860 as the reference year.

We carried out this investigation in the territory of the Autonomous Province of South Tyrol (Italy) in the Central Alps in northern Italy (Fig. 4). The area covers a surface of 7399 km^2^. The elevation ranges from 194 to 3893 m above sea level (a.s.l.), with about 40% of the area above 2000 m a.s.l. South Tyrol's landscape is extremely diverse due to the wide climatic, geological and topographical ranges as well as the long history of human settlement (see Fig. 4A). Accordingly, South Tyrol is a region with extremely high biodiversity^63^. In this study, we have interpreted trends in game abundance in terms of changes within their habitats. Specialists in particular only occur in certain ecoregions, whereas others are habitat ubiquist species. We define ecoregions in the sense of^12^ as landscape units that have developed through the interaction of site conditions (biotic and abiotic), agricultural and forestry use, as well as settlement and infrastructure development. Thus, in South Tyrol, in addition to the agriculturally used ecoregions in the valley floors and at the valley slopes in the colline to montane elevation zone, there is also an extensive forest ecoregion and a subalpine-alpine ecoregion (mostly used as alpine summer pastures). Anthropogenic pressure is most pronounced in the agriculturally used ecoregions, where most people live, the most infrastructure is found and the most intensive forms of agriculture are practised. These ecoregions have been undergoing massive changes since 1860. In the colline belt, the mosaic of grassland and arable land has given way to a monoculture of orchards and vineyards (see Fig. 4B). In the montane belt, farmers are specialised in dairy farming, and many grasslands have been intensified to increase production (Fig. 4B). Hard-to-reach, steep and high-elevation slopes, on the other hand, have been increasingly abandoned, including many alpine pastures. Accordingly, the forest has spread there. However, the forest has also undergone massive changes. Around 150 years ago, about half of all forests were semi-open (crown cover < 70%); this proportion has dropped to about 22%^12^. In addition, the higher elevations are coming under increasing pressure from rising tourism intensity and new infrastructures. Thanks to the extensive network of hiking trails, numerous ski resorts and mountain huts offering lodging and meals, more and more recreationists and tourists can easily reach these areas in both summer and winter.

The study area covers 145 hunting grounds. Because it was not feasible to collect all landscape variables (some had to be mapped manually) for the entire study region, we selected 40 hunting grounds to analyse the drivers of population dynamics (Fig. 4A). These hunting grounds are representative of geographic and geological variability as well as of elevation zones and LULC types of South Tyrol.

### Methods

#### Research design

**Figure 5:**
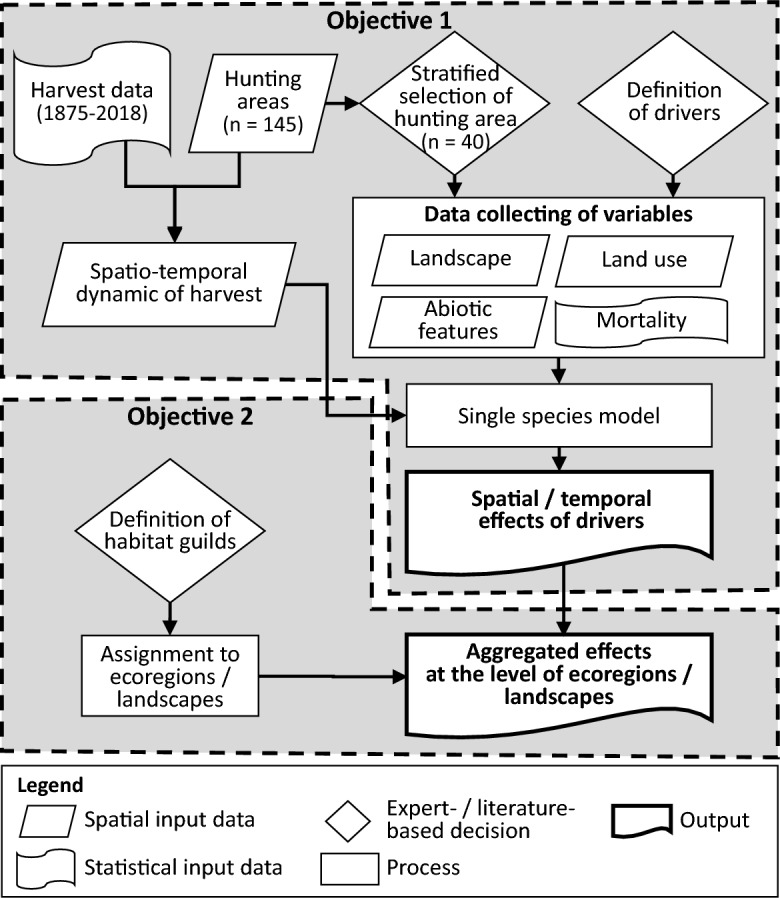
Conceptual design used in the study.

Our objectives (see Fig. 5) were to analyse the drivers of population dynamics of single game species over the past 150 years (Objective 1) and to derive general conclusions based on habitat guilds about the aggregated effects at the ecoregion and landscape levels (Objective 2).

We based the analyses on collected harvest statistics from 1875 to 1895 and then again yearly from 1953 onwards for 28 game species at the hunting-district level (for more details, see^64,65^). To assess potential variables for the spatio-temporal population dynamics, we conducted expert surveys and a literature review. We defined four main variable groups (abiotic variables, landscape, land use and mortality) and 12 subgroups, covering a total of 130 variables. We compiled all variables for the 40 hunting grounds and four time periods between the years 1875 and 2014 (Appendix A) for which landscape maps were also available (total sample size = 160). The periods were 1875–1895, 1955–1964, 1975–1984 and 2005–2014. For a detailed spatial and temporal representation of the harvest statistics, however, we also considered the periods 1965–1974, 1985–1994, 1995–2004 and 2015–2018.

#### Harvest data

We obtained harvest data from the end of the nineteenth century from statistical reports and the statistical yearbooks of the Ministry of Agriculture^66,67^. From 1953 onwards, the South Tyrolean hunting association has provided yearly harvest statistics for the 28 game species on the hunting ground level. The hunting system has changed only insignificantly since the province of South Tyrol took over responsibility in 1950. The number of maximum harvests generally follows a shooting plan drawn up by a commission consisting of five members. During planning, strict attention is paid to ensuring that the number of kills does not endanger the population, nor may such a high density be allowed that agricultural and forestry crops are excessively affected. Furthermore, strict attention has always been paid to ensuring that all harvested individuals have been properly reported by the hunters^45^, which ensures high-quality hunting data. We also collected hunting statistics from the nineteenth century for the hunting grounds, but the original data have been lost. Only data at the district level were available, which we converted to the assigned hunting grounds using data from the period 1955–1964. This conversion is subject to a certain degree of uncertainty.

We used the mean harvest number per species and year (n ha^−1^ a^−1^) based on species-specific habitats (Appendix C, Table C1) per hunting ground and per 10-year time period for modelling (see also^68^). The sample size for the single species ranged from 39 (song thrush) to 160 (for most species) (see also Appendix E, Tables E1–E4).

Although wild species exhibit individual long-term dynamics in response to environmental changes and human pressures, studies have shown that considering a guild of several species colonising the same habitats allows drawing general conclusions about the qualitative development of these habitats^7,13,16,22^. For practical relevance, we chose habitat guilds because most political, administrative and legal measures in South Tyrol are targeted at specific habitats (e.g. forest, agricultural land, alpine summer pasture and settlement area). Therefore, we assigned the 28 game species to habitat guilds by considering the preferred habitats based on a literature review (see^68^).

1. *Ungulates* (roe deer (*Capreolus capreolus*), red deer (*Cervus elaphus*), chamois (*Rupicapra rupicapra*) and wild boar (*Sus scrofa*)): Ungulates are generalists but prefer large and highly diverse forests, as well as mosaics of open cultivated areas and forests^69^. Negative impacts on habitat quality result from infrastructure (e.g. roads, fences, energy production plants and tourist resorts) and other forms of human disturbances (e.g. hiking and skiing)^70^.
2. *Mesocarnivores* (stone marten (*Martes foina*), red fox (*Vulpes vulpes*), Eurasian badger (*Meles meles*) and pine marten (*Martes martes*)): the mammalian predators require—similarly to ungulates—large forest-dominated habitats^71^. However, many of these species have expanded their ranges in the last centuries into the open rural and urban environments due to the higher food availability^72^.
3. *Small alpine game species* (mountain hare (*Lepus timidus*), Alpine marmot (*Marmota marmota*) and rock ptarmigan (*Lagopus muta*)): these species prefer open, forest-free habitats in the subalpine to the nivale belt with various protection and feeding possibilities^73^.
4. *Small forest game species* (capercaillie (*Tetrao urogallus*), black grouse (*Lyrurus tetrix*), hazel grouse (*Tetrastes bonasia*), Eurasian woodcock (*Scolopax rusticola*) and Eurasian jay (*Garrulus glandarius*)): these species prefer a mix of intact mature forests and semi-open forests, as well as forest management practices that emulate natural disturbance processes^74^.
5. *Small farmland game species* (grey partridge (*Perdix perdix*), common quail (*Coturnix coturnix*), common pheasant (*Phasianus colchicus*), rock partridge (*Alectorix graeca*), common wood pigeon (*Columba palumbus*) and brown hare (*Lepus europaeus*)): the ideal habitat for these species consists of a mosaic of extensively used arable land, orchard meadows and grasslands with hedgerows and standing open waters^22^.
6. *Synanthropic game species* (Eurasian magpie (*Pica pica*), carrion crow (*Corvus corone*), common blackbird (*Turdus merula*), song thrush (*Turdus philomelos*) and fieldfare (*Turdus pilaris*)): as generalists and omnivorous, these species occupy a wide variety of habitats such as agricultural landscapes and forests and respond positively to urban sprawl^75^.
7. *Waterfowl* (mallard (*Anas platyrhynchos*), garganey (*Anas querquedula*), common teal (*Anas crecca*) and Eurasian coot (*Fulica atra*)): these species are water bound and are therefore found near slow-running rivers, lakes and wetlands^44^.

#### Potential explanatory variables

To identify drivers, we conducted an inquiry by asking 40 gamekeepers and four authority representatives for hunting about the development of species populations in recent decades and their assessment of the driving forces. The participants were informed before the interview that participation in the survey was voluntary and anonymous and that no personal data are requested. Therewith, the survey was in line with the principles established by national and international regulations, including the Declaration of Helsinki (64th WMA General Assembly, Fortaleza, Brazil, Oct 2013) and the Code of Ethics. All drivers mentioned in the survey together with those found in the literature review via http://www.sciencedirect.com/ and https://scholar.google.com/ formed the basis to define the four main variable groups (abiotic variables, landscape, land use and mortality) and 12 subgroups (Table 1). All spatial data were projected in the UTM-WGS84 coordinate system (zone 32N). The resolution of the maps was harmonised to 5 × 5 m using the resample tool of the ESRI ArcMap 10.6 software. In sum, we collected 130 variables for the selected 40 hunting grounds (for more details, see Table  A1 in the Appendix).

**Table 1:** Overview of explanatory variables for the single species models, with details of total number of variables, their data sources, methods used, and spatial resolution (for more details see Appendix A). If there are several variables per group with different data sources and methods, the letters in the brackets indicate the corresponding references.

Variables (n)	Description	Data sources	Methods	Resolution
Abiotic drivers				
Elevation zones (5)	Elevation zones from montane to nival belt	https://natura-territorio.provincia.bz.it		5 m grid
Climate (20)	Annual and seasonal temperature and precipitation	HISTALP (http://www.zamg.ac.at/histalp)		5-min grid
Landscape				
Ecoregions (10)	Differently used ecoregions	Own mapping	^12^	1:25.000
Landscape diversity (16)	LULC heterogeneity levels for main land-use types (a), Patch richness density (b), Shannon diversity index (c)	(a) Own mapping	(a)^12^, (b, c)^76^	1:25.000
Landscape structures (19)	Structure classes (a), sprawl (b), landscape metrices (c)	(a, b) Own mapping	(a, b)^12^, (c)^76^	1:25.000
LULC types (24)	LULC (land use/land cover) types	Own mapping	^12^,	1:25.000
Mortality				
Hunting (6)	Hunting are, hunter density, release, protection status	South Tyrolean hunting association	Census	Hunting ground
Diseases (3)	Rabies, distemper, mange	Animal Disease Control Institute Bozen	Census	Hunting ground
Competition and predation (2)	Harvested predators, competitor	South Tyrolean hunting association	Census	Hunting ground
Land use				
Land-use types (9)	Main land-use types	Own mapping after	^12^	1:25.000
Land-use intensity (11)	Livestock densities (a), cutting frequency (b), amount of fertilizer (c), hail nets (d), tourism density (e), distance to nature (f)	(a)^77^, (d)^78^, (e)^79^, (f)^80^	(a) census, (b)^81^, (c)^82^	(a, d) Hunting area, (b, c, f) 5 m grid, (e) 100 m grid
Use of pesticides (5)	Pesticides within land-use types	http://www.istat.it^83,84^	Census	Hunting area

With all possible independent variables, we performed principal component analyses to identify contextual components, representative variables for components and original variables that did not load on any principal component (see Appendix D).

#### Analysis

##### Single species model

We applied generalized linear models (GLMs) to estimate the relationship between harvested individuals per period and hunting ground and the selected variables/components for each of the 28 game species. Therefore, we e used the gamma distribution and the natural logarithm as the link. The gamma distribution can take on a wide range of shapes and is suited to deal with heteroscedasticity in non-negative data^85^. Depending on the game species, a few variables had no variation and, therefore, we excluded them from the analysis. We ultimately selected the model with the smallest Akaike information criterion (AIC)^86^ dependent on the included variables obtained via a stepwise model selection procedure. We checked for outliers, the model fit and influential observation units (via residual diagnostics and statistical measures)^87^.

A priori to model estimation, we checked for multi-collinearity of the variables with the variance inflation factor (VIF). We excluded variables with VIF values > 10^88^. However, we considered these multi-collinearities when interpreting the single-species models; variables that highly correlate with other variables in the model are indicated by letter suffixes in the results (see Appendix E, Tables E1–E4).

Because we considered longitudinal data (although we used the means for 10-year periods), we included, as a robustness check, the one-period time lag of the dependent variable as an independent variable in the model. With this approach, we lost the observations of one time period for each species model. The findings remained qualitatively the same, independent of whether the lag of the dependent variable was statistically significant. Therefore, we decided to show the model results without the lag to obtain more precise estimates.

To check for overfitting—because the degrees of freedom for a few models were small—we split the sample into a training set (80% of observation units) and a validation set (20%) and calculated the root-mean-squared error (RMSE) between fitted/predicted and observed values for both the training and validation sets (see Appendix F). We repeated this procedure 1000 times. We calculated the mean and the standard deviation of the RMSE for the training set (RMSE_T_) as well as the mean of the validation set (RMSE_V_). For all of our models, the mean RMSE of the validation set was in the distribution of the RMSE of the training set; hence, we assumed that overfitting was not a problem.

For the final model (Appendix E), we provide (i) the sample size and the model degrees of freedom (df), (ii) the p-value of the test concerning the significance of the model as a whole, i.e. null model (just intercept) versus proposed model (pvalue1), (iii) the p-value of the residual deviance test concerning model fit, i.e. proposed model (null hypothesis) versus saturated model (pvalue2), and (iv) as pseudo-R^2^ measure, the squared correlation between the estimates of the dependent variable and the observed values both at the response and link level.

We used the software R^89^ for all calculations. To determine the VIF, we used the car package^90^.

##### Deduction of compiled effects at the level of ecoregions/landscapes

The deduction of aggregated effects at the level of ecoregions/landscapes is based on the assignment of species to habitat guilds. For this purpose, we counted for each variable how often it exerted a statistically significant association (p < 0.1) in the single-species models per habitat guild. Specifically, we counted the number of significant correlations and related this number to the total number of potential correlations per variable or variable group. Thus, the resulting frequency reflects the importance of the variable groups and subgroups for the habitat guilds present in the ecoregion or landscape. Therefore, it is also a measure that quantifies the possible causes of change in the regions.

### Supplementary Information

Supplementary Information.

## Data Availability

Input data used in this analysis are cited in the text. Results data are available in the Appendices. The whole datasets used and analysed during the current study available from the corresponding author on request.
