# Supplementary material for: Drivers of spatio-temporal population dynamics of game species in a mountain landscape
Source: Sci Rep. 2024 Feb 1;14:2740. doi: 10.1038/s41598-024-53019-x (PMC10834489; doi:10.1038/s41598-024-53019-x)
Supplement: Supplementary file 1 — Supplementary Information. [file 41598_2024_53019_MOESM1_ESM.docx]

**Appendix**

Appendix A: Potential explanatory variables

To explain the variability of the amount of harvested game species we used a set of 128 potential explanatory variables (see Table A1). Therein were variables on climate, landscape diversity and structures, land cover, hunting, wildlife diseases, competition and predation, land use type and intensity (including pesticide use).

***Abiotic variables***

- Elevation zones: To include the long-term climate and environmental conditions in the analysis, the proportion of the area of each elevation zones (see ^1^ per hunting area was included in the evaluation. We distinguished 5 zones: colline (<800 m a.s.l.), montane (800-1600 m a.s.l.), subalpine (1600-2400 m a.s.l.), alpine (2400-3200 m a.s.l.) and nival (>3200 m a.s.l.).
- Climate: Mean annual and seasonal temperature and mean precipitation as well as the standard deviation for temperature and precipitation were calculated for each of the selected hunting grounds and for the respective 10-year time steps. Temperature and precipitation data are from the HISTALP project (http://www.zamg.ac.at/histalp). 5x5-minute grid files for historical and current temperature ^2,3^ and precipitation ^4^ distributions were downloaded and interpolated for the hunting areas in South Tyrol.

***Landscape variables***

As a basis for the different landscape variables, the LULC was mapped (see Fig. 1) after the method of ^5^. This approach uses information from different sources such as historical maps and orthophotos, interviews and census data to map the land-use development of four-time steps (1856, 1953, 1985 and 2006) on a scale of 1:25,000. The mapped LULC types are based on phytosociological principles and are hierarchically classified into natural, near-natural and artificial habitat types found in the Central and Southern Alps.

- Ecoregion: We subdivided the study area into 10 ecoregions: agriculturally used valley bottom (colline/montane), agriculturally used valley slopes (colline/montane), forest region (colline/montane/subalpine), agriculturally used alpine pastures, near-natural grassland region (alpine belt) and vegetationless region (nival belt). For each hunting ground the proportion of ecoregions (%) was calculated.
- Landscape diversity: Based on LULC mapping the heterogeneity of the LULC types was estimated. As monocrop (heterogeneity level 1) an area where only one LULC type covers up at least 95% of the total area is defined. Level 2 indicates an area having a share of 5 and 25% of other LULC types within a main type. Heterogeneity level 3 refers to an area with a share of 25 and 50% of other LULC types (for more details see ^5^). Furthermore, we calculated the Patch richness density (PRD) and Shannon diversity index (SHDI) on the landscape level using FRAGSTATS v4.2.1.
- Landscape structures: Five non-scale-sensitive landscape metrics relevant for mammals and birds were calculated based on a selection approach (see Appendix B): Patch density (PD), Edge density (ED), Mean shape index (MSI), Total core area (TCA), Euclidean mean nearest neighbour distance (ENN_AM). These metrics were computed on the landscape and the class level FRAGSTATS v4.2.1 (for more details on the metrics, see ^6^). b) Structuring degree: The number of structural elements such as groves, hedges, single trees, banks, debris areas and smaller habitats (i.e. marshland, rocks, and debris acres) per homogenous area were counted and the percentage (%) of the areas with different structuring degrees was calculated ^5^.
- Urban sprawl: the proportion of settlement areas in the hunting area (%) and the building density in open cultural landscapes (n ha^-1^) were calculated. As a settlement area, an area with less than 25% of green areas (gardens, parks) is defined and by building, we mean a building having a ground area of more than 16 m^2^.
- LULC type: In South Tyrol, 24 LULC types occur with a cover percentage of more than 1% (see Table A1; ^5^. The shares (%) of these LULC types per hunting area were included in the analysis.

***Management variables***

- Land-use type: Hay meadows, alpine summer pastures, orchards and vineyards are important when discussing landscape dynamics in South Tyrol. Therefore, the main land-use types (permanent grassland, arable field, permanent culture, forest, unused) were mapped for the selected 40 hunting grounds (for methodical aspects see ^5^) and the proportions of these per hunting ground were used as explanatory variables.
- Land-use intensity: In this study, the following intensity variables were included:
  a) Cutting frequency of hay meadows: It can influence food availability for single species, survival of juveniles (e.g. hares, roe deer) and breeding success of ground-nesting birds. The assessment of historical and current cutting frequency is based on survey data and historical records ^7^. Elevation and cutting frequency were correlated for the different periods, based on which the cutting frequency map were modelled for the years 1856, 1953, 1985 and 2006.
  b) Livestock density: Information on livestock units for important livestock breedings (cattle, equidae, sheep, goats) and the total density of livestock units within an area (LSU) were integrated to take account of changes in animal husbandry over time. As ^8^ show, the number of small ruminants has decreased significantly at the expense of cattle husbandry in the region.
  c) Amount of fertilizer: Based on the number of LSU, the nitrogen output was calculated via fertilizer livestock unit (FLU) per hunting ground and time step. A FLU corresponds to the annual amount of liquid manure produced by a cow weighing 600 kilograms of 105 kg of nitrogen ^9–11^.
  d) Hail nets: Orchards and viticulture are cultivation forms vulnerable to hail damage. Therefore, the use of hail nets in orchards increased strongly over the last decades ^12^ reconstructed the spatio-temporal expansion of hail nets in the middle Etsch valley since 1996, giving the proportion of area covered by hail nets. We used the proportion to reconstruct the expansion of hail nets for the rest of South Tyrol.
  e) Tourism intensity: It was quantified using a recreation flow map developed by ^13^. The researchers used social media meta-data to create a point density map of photo density in the Alps (n km^-2^). The map was clipped with the 40 hunting grounds investigated in this study and was used to calculate the mean value of tourism activity.
  f) Distance to nature: We calculated on basis of the habitat maps for all time steps the distance to nature (D2N) suggested by ^14^. The composite index incorporates the degree of naturalness (N_d_) and distance to natural habitat (D_n_) and describes the influence of anthropogenic land use on biodiversity. The scale goes from zero to one, where 0 stands for natural habitats and 1 represents artificial habitats. The mean value and standard deviation of the index were computed at pixel level for each hunting ground.
- Use of pesticides: The systematic use of pesticides started in Europe in the 1950ies. However, reliable data on the amount of pesticides purchased in South Tyrol only became available from 1996 onwards (www.istat.it). Assuming a similar development in the amount of pesticides used in Italy and Germany ^15,16^, the amount of used pesticides (kg ha^-1^) were reconstructed for South Tyrol back until 1950. The amount of pesticides used increased massively from 1960 onwards and reached a peak at the beginning of the 1990s with 180 kg ha^-1^ per year in orchards and 73 kg ha^-1^ in viticulture. Since then, the amount of pesticides used has decreased to about 85 kg ha^-1^ in orchards and 31 kg in vineyards in 2019. With 5 kg ha^-1^, the amount of pesticide used in arable farming is significantly lower. However, the trends in quantities is partly caused by the fact that over time the efficacy of the preparations used has increased significantly ^17^. To break down information to the hunting ground level, the amount of pesticides purchased was standardized via the a) total hunting area, b) the area of orchards, c) vineyards and d) permanent crops (sum of orchards and vineyards) present per hunting ground for the single time steps. Possible resale of pesticides and changes in composition and concentration of the products over time were not considered.

***Mortality***

In Europe, hunting ^18^ and periodically recurring game diseases ^19^ are seen as the main mortality reasons for game species at the adult stage. Juvenile mortality is strongly influenced by the lack of food, weather influences, predation and competition.

- Hunting: a) Adjusted hunting area (ha) was included as a variable and gives the total area per hunting ground. By law, hunting is prohibited in South Tyrol in and close to settlement areas. Therefore, we corrected the hunting area by excluding settlement areas and the bordering buffer zone of 100 m. b) Hunter density gives the 10-year mean number of hunters (provided by the South Tyrolean hunting association) per hunting ground for the four time steps. c) Time of hunting management incorporates the time period of active hunting regulations in the region, starting with zero years in 1953 and ending with 65 years of hunting management in 2017. Thus, this variable is also a variable that reflects the time effect. d) Release: Active release by the hunters has only been carried out on common pheasant (between 1953 and 1997). e) Protection status: Some species, such as partridge or badger, were temporarily protected due to critical population dynamics and thus could not be harvested (see Tasser et al. 2023). This information was integrated into the analysis using a separate variable (protection yes or no).
- Diseases: Data on wildlife disease (rabies, mange and distemper) were provided by the Animal Disease Control Institute Bozen, the Office for Hunting and Fishing and the South Tyrolean hunting association and contain officially reported cases (n) for every hunting ground, which were included as variables.
- Competition and predation: Within the scope of an intensive literature review, predators and interspecific competitors were searched for the game species, which were included in the individual species models as further explanatory variables. However, only those predator species for which reliable population data or harvest data are available could be considered. These species include all birds of prey. Bears could be detected sporadically in South Tyrol since 2005. Wolves have been present in South Tyrol as migrants since 2000 and permanently since 2018. However, their low numbers so far, the short time span of their return, and their complex interaction with meso-carnivores do not yet allow conclusions on the impact of these large carnivores on the harvested games. Important predators limiting nest success and reproduction and/or general predators are:

1. Red fox: for roe deer ^20^, Alpine marmot ^21^, European hare ^22,23^, mountain hare ^24^, Capercaillie ^25,26^, black grouse ^26^, rock ptarmigan ^27^, rock partridge ^28^, grey partridge ^29^, common pheasant ^30^ and duck species ^31^.
2. European Badgers: for rock partridge ^28^
3. Martens: for Capercaillie and black grouse ^26^, rock partridge ^28^ and common wood pigeon ^32^.
4. Raven crow: Capercaillie and black grouse ^26^, common pheasant ^30^ and duck species ^31^.

Furthermore, interspecific competitions for access to food between some game species are known, such as between the red and roe deer ^33^ and magpies and crows ^34^. In addition, there is of course also a food competition with livestock breedings. This interspecific competition is used as a land-use intensity variable in this study.

Table A1 The explanatory variables collected for the explanation of the harvest data.
Abbreviations: s.d. = standard deviation, a = *annus*.

| **Variables** | **Description** | **Unit** | **Min** | **Median** | **Max** | **Mean** |
| --- | --- | --- | --- | --- | --- | --- |
| **Abiotic drivers** | | | | | | |
| Elevation zones | | | | | | |
| C_zone | Colline (<800 m a.s.l.) | % | 0.0 | 0.0 | 76.5 | 8.1 |
| M_zone | Montane (800-1600 m a.s.l.) | % | 4.9 | 39.1 | 82.6 | 37.7 |
| SA_zone | Subalpine (1600-2400 m a.s.l.) | % | 0.0 | 41.5 | 67.8 | 39.4 |
| A_zone | Alpine (2400-3200 m a.s.l.) | % | 0.0 | 11.1 | 52.3 | 12.9 |
| N_zone | Nival (>3200 m a.s.l.) | % | 0.0 | 0.0 | 16.8 | 1.1 |
| Climate | | | | | | |
| T_10 | Mean annual temperature | °C | -2.4 | 3.0 | 11.9 | 3.5 |
| T_10_s.d. | S.d. of annual temperature | °C | 0.4 | 0.6 | 0.9 | 0.6 |
| T_SP_10 | Mean spring temperature (months 4-6) | °C | -0.9 | 5.9 | 17.0 | 6.5 |
| T_SP_10_s.d. | S.d. of spring temperature (months 4-6) | °C | 0.6 | 0.8 | 1.0 | 0.8 |
| T_SU_10 | Mean summer temperature (months 7-9) | °C | 4.7 | 10.4 | 20.5 | 11.1 |
| T_SU_10_s.d. | S.d. of summer temperature (months 7-9) | °C | 0.5 | 0.8 | 1.2 | 0.9 |
| T_A_10 | Mean autumn temperature (months 10-12) | °C | -4.8 | -0.3 | 6.3 | 0.0 |
| T_A_10_s.d. | S.d. of autumn temperature (months 10-12) | °C | 0.7 | 1.0 | 1.6 | 1.0 |
| T_W_10 | Mean winter temperature (months 1-3) | °C | -9.3 | -4.1 | 3.7 | -3.8 |
| T_W_10_s.d. | S.d. of winter temperature (months 1-3) | °C | 0.7 | 1.2 | 1.6 | 1.2 |
| P_10 | Mean annual precipitation | mm | 796 | 996 | 1603 | 1043 |
| P_10_s.d. | S.d. of annual precipitation | mm | 58 | 144 | 215 | 137 |
| P_SP_10 | Mean spring precipitation (months 4-6) | mm | 198 | 287 | 455 | 293 |
| P_SP_10_s.d. | S.d. of spring precipitation (months 4-6) | mm | 18 | 60 | 87 | 55 |
| P_SU_10 | Mean summer precipitation (months 7-9) | mm | 249 | 354 | 601 | 366 |
| P_SU_10_s.d. | S.d. of summer precipitation (months 7-9) | mm | 38 | 62 | 133 | 67 |
| P_A_10 | Mean autumn precipitation (months 10-12) | mm | 172 | 220 | 363 | 235 |
| P_A_10_s.d. | S.d. of autumn precipitation (months 10-12) | mm | 45 | 69 | 101 | 70 |
| P_W_10 | Mean winter precipitation (months 1-3) | mm | 110 | 149 | 264 | 154 |
| P_W_10_s.d. | S.d. of winter precipitation (months 1-3) | mm | 16 | 68 | 151 | 75 |
| **Landscape** | | | | | | |
| Ecoregion | | | | | | |
| ER_C_VB | Agriculturally used valley bottom, colline zone | % | 0.0 | 0.0 | 39.3 | 2.6 |
| ER_C_S | Agriculturally used valley slopes, colline zone | % | 0.0 | 0.0 | 31.1 | 3.0 |
| ER_C_F | Forest belt, colline zone | % | 0.0 | 0.0 | 28.2 | 2.6 |
| ER_M_VB | Agriculturally used valley bottom, montane zone | % | 0.0 | 5.8 | 36.7 | 8.0 |
| ER_M_S | Agriculturally used valley slopes, montane zone | % | 0.0 | 4.7 | 38.4 | 9.4 |
| ER_M_F | Forest belt, montane zone | % | 0.0 | 17.8 | 52.3 | 20.4 |
| ER_SA_F | Forest belt, subalpine zone | % | 0.0 | 18.5 | 50.1 | 20.8 |
| ER_SA_P | Agriculturally used alpine pastures | % | 0.0 | 19.0 | 35.7 | 18.6 |
| ER_A | Near-natural grassland belt, alpine zone | % | 0.0 | 11.1 | 52.3 | 12.9 |
| ER_N | Vegetationless belt, nival zone | % | 0.0 | 0.0 | 16.8 | 1.1 |
| Landscape diversity | | | | | | |
| H_F_lev2 | LULC heterogeneity level 2 in forest | % | 0.00 | 0.48 | 59.34 | 3.83 |
| H_F_lev3 | LULC heterogeneity level 3 in forest | % | 0.00 | 0.00 | 12.45 | 0.55 |
| H_PC_lev2 | LULC heterogeneity level 2 in permanent cultures | % | 0.00 | 0.00 | 7.86 | 0.27 |
| H_PC_lev3 | LULC heterogeneity level 3 in permanent cultures | % | 0.00 | 0.00 | 2.76 | 0.08 |
| H_AL_lev2 | LULC heterogeneity level 2 in arable land | % | 0.00 | 0.00 | 8.69 | 0.90 |
| H_AL_lev3 | LULC heterogeneity level 3 in arable land | % | 0.00 | 0.00 | 9.40 | 0.46 |
| H_G_lev2 | LULC heterogeneity level 2 in grassland | % | 0.00 | 5.35 | 37.00 | 8.01 |
| H_G_lev3 | LULC heterogeneity level 3 in grassland | % | 0.00 | 1.87 | 21.08 | 3.06 |
| H_AB_lev2 | LULC heterogeneity level 2 in abandoned land | % | 0.00 | 0.00 | 8.87 | 0.38 |
| H_AB_lev3 | LULC heterogeneity level 3 in abandoned land | % | 0.00 | 0.00 | 8.48 | 0.23 |
| H_S_lev2 | LULC heterogeneity level 2 in settlements | % | 0.00 | 0.00 | 0.83 | 0.04 |
| H_S_lev3 | LULC heterogeneity level 3 in settlements | % | 0.00 | 0.00 | 1.56 | 0.02 |
| H_NU_lev2 | LULC heterogeneity level 2 in agricultural not-usable areas | % | 0.00 | 0.00 | 1.99 | 0.12 |
| H_NU_lev3 | LULC heterogeneity level 3 in agricultural not-usable areas | % | 0.00 | 0.00 | 0.85 | 0.02 |
| SHDI | Shannon diversity index (SHDI), landscape level | index | 0.04 | 0.16 | 1.47 | 0.20 |
| PRD | Patch richness density (PRD), landscape level | n 100 ha^-1^ | 0.59 | 1.32 | 1.78 | 1.27 |
| Landscape structures | | | | | | |
| S_10 | Not or scarcely structured woodless areas (0-0.25 objects ha^-1^) | % | 0.0 | 2.9 | 41.3 | 8.2 |
| S_20 | Structured woodless areas (>0.25-2.5 objects ha^-1^) | % | 0.0 | 6.6 | 26.3 | 7.7 |
| S_30 | Richly structured woodless areas (>2.5 - 5 objects ha^-1^) | % | 0.0 | 23.1 | 81.2 | 29.6 |
| S_40 | Wooded grassland and pasture (>5 single trees in the area, but <30% crown closure) | % | 0.0 | 0.5 | 6.1 | 1.0 |
| S_41 | Wooded grassland and pasture, share of young trees >70% | % | 0.0 | 0.3 | 7.3 | 0.8 |
| S_42 | Wooded grassland and pasture, share of old trees >70% | % | 0.0 | 0.0 | 9.5 | 0.7 |
| S_51 | Sparse forest stock (crown closure 30% - 70%) | % | 0.0 | 3.7 | 40.8 | 6.4 |
| S_52 | Dense forest stock (crown closure >70%) | % | 1.7 | 41.0 | 76.5 | 41.9 |
| Sprawl | Building density in open cultural landscapes | n ha^-1^ | 1.1 | 11.7 | 72.4 | 14.6 |
| PD_l | Patch density, landscape level | n 100 ha^-1^ | 0.1 | 0.8 | 8.8 | 1.0 |
| ED_l | Edge density, landscape level | m ha^-1^ | 7.9 | 29.4 | 61.7 | 29.9 |
| MSI_l | Mean shape index, landscape level | index | 1.3 | 2.2 | 2.8 | 2.2 |
| ENN_AM_l | Euclidean mean nearest neighbour distance, landscape level | m | 42.0 | 65.8 | 1279.1 | 110.9 |
| TCA_l | Total core area, landscape level | m² ha^-1^ | 218 | 3068 | 17286 | 4178 |
| PD_cl | Patch density, class level | n 100 ha^-1^ | 0.0 | 0.4 | 1.5 | 0.5 |
| ED_cl | Edge density, class level | m ha^-1^ | 7.8 | 27.0 | 57.0 | 28.0 |
| MSI_cl | Mean shape index, class level | index | 1.4 | 2.1 | 3.0 | 2.1 |
| ENN_AM_cl | Euclidean mean nearest neighbor distance, class level | m | 40.0 | 69.6 | 878.9 | 118.8 |
| TCA_cl | Total core area, class level | m² ha^-1^ | 120 | 1357 | 13792 | 2370 |
| LULC types | | | | | | |
| SH_111000 | Lake | % | 0.0 | 0.0 | 3.5 | 0.2 |
| SH_113000 | Watercourse | % | 0.0 | 0.0 | 1.8 | 0.2 |
| SH_122100 | Gravel-and-sand bank | % | 0.0 | 0.0 | 2.0 | 0.0 |
| SH_131000 | Wetland, moor | % | 0.0 | 0.0 | 1.4 | 0.1 |
| SH_133000 | Cane brake | % | 0.0 | 0.0 | 2.6 | 0.0 |
| SH_210000 | Knee timber | % | 0.0 | 0.0 | 9.5 | 0.9 |
| SH_212000 | Green alder shrub | % | 0.0 | 0.0 | 1.6 | 0.1 |
| SH_221000 | Sub-alpine coniferous forest | % | 0.0 | 21.1 | 77.0 | 20.8 |
| SH_222100 | Montane spruce-fire forest | % | 0.0 | 16.4 | 63.3 | 17.5 |
| SH_222200 | Pine-forest | % | 0.0 | 0.7 | 27.1 | 3.8 |
| SH_230000 | Wet forest | % | 0.0 | 0.0 | 5.4 | 0.2 |
| SH_232000 | Thermopile oak forest | % | 0.0 | 0.0 | 21.7 | 1.8 |
| SH_234000 | Mesophilic mixed deciduous forest | % | 0.0 | 0.0 | 24.0 | 1.6 |
| SH_310000 | Alpine pioneer formation | % | 0.0 | 0.0 | 8.4 | 0.6 |
| SH_311000 | Glacier and snow-pack | % | 0.0 | 0.0 | 21.9 | 1.3 |
| SH_313000 | Rock | % | 0.0 | 4.0 | 21.7 | 6.0 |
| SH_314000 | Scree slope | % | 0.0 | 3.5 | 23.8 | 5.0 |
| SH_322000 | Natural alpine grassland | % | 0.0 | 1.4 | 27.1 | 4.6 |
| SH_322100 | Natural alpine grassland on basic soils | % | 0.0 | 0.0 | 11.2 | 0.4 |
| SH_323000 | Natural alpine grassland on silicatic soils | % | 0.0 | 1.3 | 24.2 | 4.2 |
| SH_412000 | Xeric grassland | % | 0.0 | 0.0 | 44.9 | 1.6 |
| SH_413000 | Bushland | % | 0.0 | 0.0 | 2.1 | 0.1 |
| SH_451000 | Orchard meadow | % | 0.0 | 0.0 | 13.3 | 0.9 |
| SH_452000 | Larch meadow | % | 0.0 | 0.0 | 2.4 | 0.1 |
| **Mortality** | | | | | | |
| Hunting | | | | | | |
| Hunt_area | Hunting area | km² | 4.8 | 46.4 | 209.7 | 63.5 |
| Adj_hunt_area | Adjusted hunting area | km² | 4.0 | 43.7 | 209.2 | 61.9 |
| Hunter_dens | Hunter density | n km^-2^ | 0.00 | 0.43 | 5.32 | 0.62 |
| Release | Active release by the hunters (yes or no) | 0/1 | 0.0 | 0.0 | 1.0 | 0.0 |
| Prot_status | Protection status (yes or no) | 0/1 | 0.0 | 0.0 | 1.0 | 0.0 |
| Diseases | | | | | | |
| KH_T | Rabies | n | 0.0 | 0.0 | 1.0 | 0.0 |
| KH_S | Distemper | n | 0.0 | 0.0 | 1.3 | 0.0 |
| KH_R | Mange | n | 0.0 | 0.0 | 1.0 | 0.0 |
| Competition and predation | | | | | | |
| Predators_n | Harvested predators for the single species within the hunting areas | n | 0.0 | 0.0 | 158.9 | 8.5 |
| Interspec_comp_n | Competitors for the single species within the hunting areas | n | 0.0 | 0.0 | 221.1 | 1.1 |
| **Land use** | | | | | | |
| Land-use type | | | | | | |
| LU_gras_i | Hay meadows, intensively used | % | 0.0 | 10.9 | 53.8 | 12.0 |
| LU_gras_e | Hay meadows, extensively used | % | 0.0 | 11.0 | 44.9 | 12.6 |
| LU_pasture | Alpine summer pasture | % | 0.0 | 11.2 | 45.1 | 13.2 |
| LU_arable | Arable land | % | 0.0 | 0.7 | 19.3 | 2.5 |
| LU_orchard | Orchard area | % | 0.0 | 0.0 | 30.3 | 1.5 |
| LU_vine | Vineyard area | % | 0.0 | 0.0 | 20.8 | 0.9 |
| LU_aband | Abandoned land | % | 0.0 | 0.0 | 0.0 | 0.0 |
| LU_forest | Forest area | % | 0.1 | 45.8 | 76.8 | 46.3 |
| LU_settl | Settlement | % | 0.0 | 0.4 | 10.4 | 0.8 |
| Land-use intensity | | | | | | |
| LI_LSU | Total livestock units | n | 39 | 884 | 6094 | 1159 |
| LI_LSD | Total livestock density | n ha^-1^ | 0.4 | 1.9 | 20.1 | 2.3 |
| LI_cattle | Cattle density | n ha^-1^ | 0.0 | 680.8 | 5552.0 | 968.1 |
| LI_horse | Horse density | n ha^-1^ | 0.0 | 40.1 | 324.9 | 59.7 |
| LI_sheep | Sheep density | n ha^-1^ | 0.0 | 24.2 | 344.8 | 38.1 |
| LI_goat | Goat density | n ha^-1^ | 0.0 | 8.2 | 214.2 | 22.3 |
| CUT_mean | Cutting frequency of hay meadows per annum | n a^-1^ | 0.0 | 1.7 | 3.0 | 1.7 |
| Fertilizer | Animal fertilizer | kg ha^-1^ | 0.8 | 19.0 | 105.5 | 23.3 |
| Hail_net | Hail nets | ha | 0.0 | 0.0 | 535.0 | 12.3 |
| Tourism | Tourism intensity, photo density in the Alps | n km^-2^ | 0.0 | 20625 | 978398 | 76461 |
| d2n | Mean distance to nature | index | 0.03 | 0.13 | 0.28 | 0.13 |
| Use of pesticides | | | | | | |
| Pest_total | Total amount of used pesticides within hunting area | kg a^-1^ | 0.0 | 1.0 | 171485.8 | 8388.3 |
| Pest_arable | Amount of used pesticides within the permanent crops | kg a^-1^ | 0.0 | 0.0 | 1170.7 | 62.9 |
| Pest_vine | Amount of used pesticides within vineyards | kg a^-1^ | 0.0 | 0.0 | 45683.8 | 1013.1 |
| Pest_orchard | Amount of used pesticides within orchards | kg a^-1^ | 0.0 | 0.0 | 170664.3 | 7312.3 |
| Pest_dens | Mean amount of pesticides per hunting area | kg a^-1^ ha^-1^ | 0.0 | 0.0 | 34.4 | 1.1 |
| Time trend | Time since the introduction of hunting management (time trend factor) | a | 0.0 | 22.5 | 65.0 | 27.5 |

**Appendix B: Definition of relevant landscape metrics**

To identify landscape metrics for monitoring landscape structure that may be relevant to mammals and birds, an extensive literature search was conducted at www.sciencedirect.com/ and https://scholar.google.com/. ^35^ give an overview of landscape metrics and their use in biodiversity and habitat research. The review paper contains a list summarizing statistically significant metrics for different taxa (e.g. amphibia, mammals, birds, insects and plants) and gives a first insight into the ecological meaning of these metrics. Also, we found case studies of several mammal and bird species: mule deer ^36^, moose ^37^, white-tailed deer ^38^, ruffed grouse ^39^ and analysis including several mammal and bird species ^40,41^.

As a result, we selected a set of 24 landscape metrics. To avoid inappropriate scale-effects, non-scale-sensitive metrics were favored ^42–44^. Finally 7 landscape metrics were used.

Table B1: Selected landscape metrics based on the literature study with the number of documented effects (DE#) and corresponding sources.

| **Metric** | **Metric description** | **Fragstats category** | **Level** | **DE#** | **Sources** |
| --- | --- | --- | --- | --- | --- |
| AWMSI | Area-weighted mean shape index | Shape metrics | Class/Landscape | 2 | ^43,44^ |
| CONTAG | Contagion index (%) | Contagion and interspersion metrics | Landscape | 7 | ^35–38,42,44,45^ |
| CWED | Contrast-weighted edge density (m ha^-1^) | Edge metrics | Class/Landscape | 3 | ^35,37,40^ |
| DLFD | Double log fractal dimension | Shape metrics | Class/Landscape | 2 | ^37,45^ |
| ED | Edge density (m ha^-1^) | Edge metrics | Class/Landscape | 5 | ^35–37,40,44^ |
| ENN_MN | Euclidean Mean Nearest Neighbor Distance (m) | Nearest-neighbor metrics | Class/Landscape | 5 | ^35,39,42,45^ |
| IJI | Interspersion and Juxtaposition index (%) | Contagion and interspersion metrics | Class/Landscape | 3 | ^38,42,43^ |
| LPI | Largest patch index (%) | Area metrics | Class/Landscape | 6 | ^35,39–41,44,46^ |
| MCA | Mean core area per patch (ha) | Core area metrics | Class/Landscape | 2 | ^40,41^ |
| MECI | Mean edge contrast index (%) | Edge metrics | Class/Landscape | 2 | ^36,37^ |
| MPFD | Mean patch fractal dimension | Shape metrics | Class/Landscape | 2 | ^40,42^ |
| MPS | Mean patch size (ha) | Patch density, patch size and variability metrics | Class/Landscape | 10 | ^35–37,39–42,44–46^ |
| MSI | Mean shape index | Shape metrics | Class/Landscape | 6 | ^35–37,39,42,44,47^ |
| NP | Number of patches (n) | Patch density, patch size and variability metrics | Class/Landscape | 5 | ^35,39,41,44,45^ |
| PD | Patch density (n 100 ha^-1^) | Patch density, patch size and variability metrics | Class/Landscape | 5 | ^40,42–45^ |
| PR | Patch richness (n) | Diversity metrics | Landscape | 4 | ^43–46^ |
| PRD | Patch richness density (n 100 ha^-1^) | Diversity metrics | Landscape | 3 | ^36–38^ |
| PSCV | Patch size coefficient of variation (%) | Patch density, patch size and variability metrics | Class/Landscape | 4 | ^36,37,40,43^ |
| PSSD | Patch size standard deviation (ha) | Patch density, patch size and variability metrics | Class/Landscape | 3 | ^39,41,43^ |
| SHDI | Shannon's diversity index | Diversity metrics | Landscape | 5 | ^38,39,43,44,46^ |
| TA^*^ | Total landscape area (ha) | Area metrics | Landscape | 1 | ^41^ |
| TCAI | Total core area index (%) | Core area metrics | Class/Landscape | 3 | ^38,39,41^ |
| TCAI | Total core area | Core area metrics | Class/Landscape | 1 | ^38,39,41^ |
| TECI | Total edge contrast index (%) | Edge metrics | Class/Landscape | 3 | ^38,39,41^ |

^*^ Instead of TA, the hunting area is included in the analysis.

**Appendix C: Spatial trends for three typical game species**

Figure C1 shows the harvest development of the red deer, the rock partridge and the black grouse. The red deer is one of those game species that have developed positively since the end of the 19th century. In the 19th century, it was largely extinct in South Tyrol due to intensive hunting. It was not until the middle of the 20th century that the populations slowly began to recover from the northwest. Over time, this deer species spread over the whole of South Tyrol, although the highest densities can still be found in the western part of South Tyrol in the area around the Stelvio National Park. Today in South Tyrol an average of 3800 deer are harvested per year. The black grouse, on the other hand, shows no major changes over the 150 years. The number of harvests increased slightly in the middle of the 20th century, but then decreased again until the 1980s. Since then, the number of birds harvested has remained stable at around 300 individuals per year. Finally, the example of the rock partridge shows a third development trend. The species has declined considerably over time. At the end of the 19th century, 200 rock partridges were harvested annually, but by the middle of the 20th century the number had risen to more than 550 individuals. Since then, however, the number has decreased dramatically. In recent years, only 30 rock partridges have been harvested.


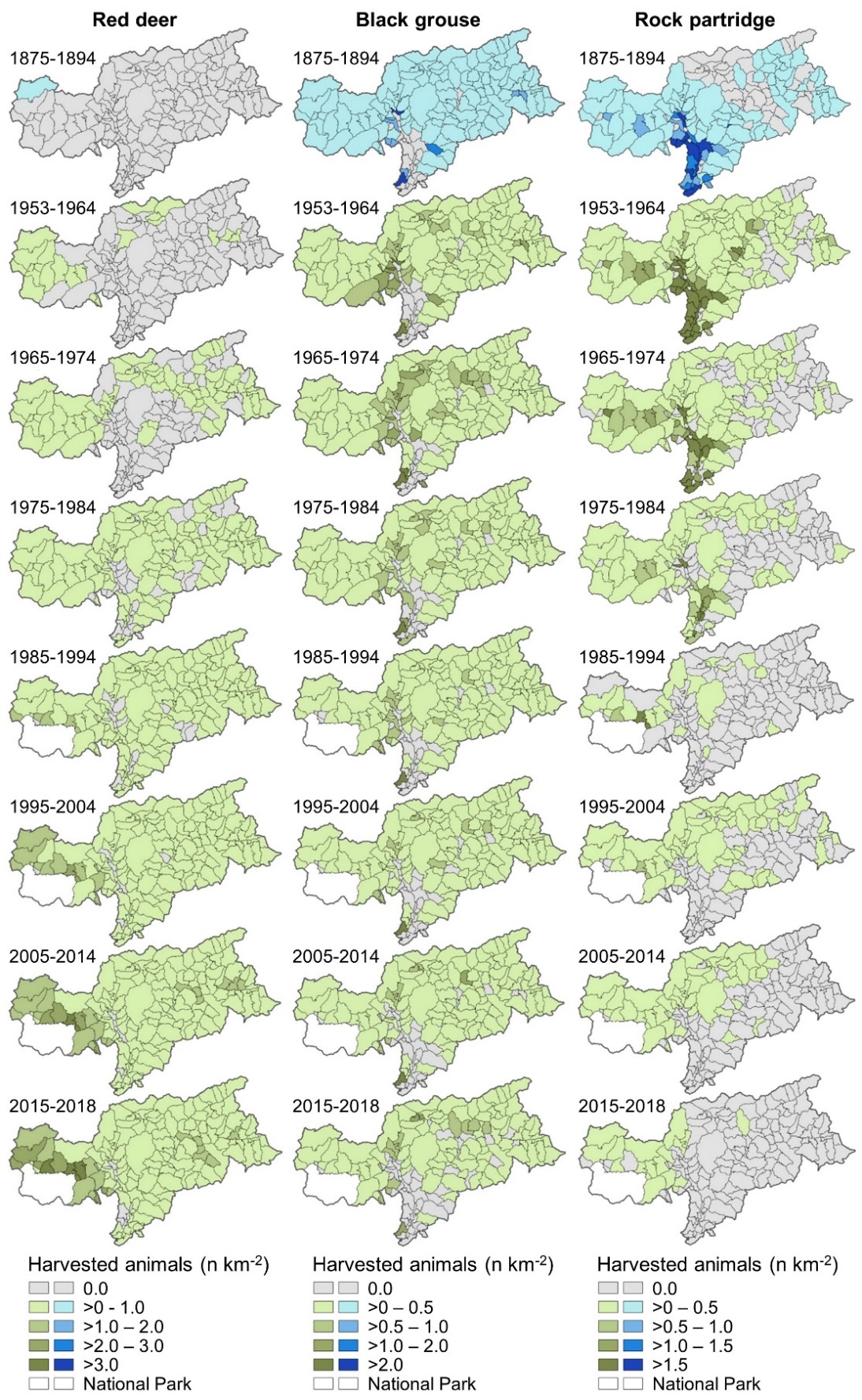


Fig. C1: Development of harvested individuals in the 145 South Tyrolean hunting grounds using red deer, black grouse and rock partridge. Harvest numbers were normalised to the species-specific habitat area per hunting ground (Table C1, changed after ^48^). Maps produced by E. Tasser using ArcGIS for Desktop (ArcMap 10.7.1, ESRI).

Table C1: Potential habitats for the individual game species as basis for the calculation of the species density.

| **Specie** | Orchards | Vineyards | Tree nursery | Arable land | Intensively managed hay meadows | Intensively managed pastures | Extensively managed hay meadows | Larch meadows | Alpine pastures | Abandoned agricultural area | Density urban area | Loose urban and rural areas | Single houses, scattered settlement | Industrial and commercial areas | Other anthropogenic areas | Montane spruce forests | Subalpine spruce forests | Larch and stone pine forests | Green alder and mountain pine | Spruce-fir forests | Oak woods | Wet forests | Beech forests | Spruce-fir-beech forests | Oak-pine forests | Hardwood forests | Pine forests | Larch forests | Manna ash-hop beech forests | Krummholz | Hedges and flywoods | Not-used alpine grassland | Rocks and screen slops | Vegetationless areas | Glacier | Moors | Watercourses | Lacks |
| --- | --- | --- | --- | --- | --- | --- | --- | --- | --- | --- | --- | --- | --- | --- | --- | --- | --- | --- | --- | --- | --- | --- | --- | --- | --- | --- | --- | --- | --- | --- | --- | --- | --- | --- | --- | --- | --- | --- |
| Wild boar | 1 | 1 | 1 | 1 | 1 | 1 | 1 | 1 | 1 | 1 |  |  | 1 |  | 1 |  | 1 |  | 1 |  | 1 | 1 | 1 | 1 | 1 | 1 | 1 | 1 | 1 |  | 1 | 1 |  |  |  | 1 | 1 | 1 |
| Chamois |  |  |  |  | 1 |  |  | 1 | 1 | 1 |  |  |  |  |  | 1 | 1 | 1 | 1 |  |  |  |  |  |  |  |  | 1 |  | 1 |  | 1 | 1 | 1 |  |  |  |  |
| Roe deer | 1 | 1 | 1 | 1 | 1 | 1 | 1 | 1 | 1 | 1 |  |  |  |  |  | 1 | 1 | 1 | 1 | 1 | 1 | 1 | 1 | 1 | 1 | 1 | 1 | 1 | 1 | 1 | 1 | 1 |  | 1 |  | 1 | 1 |  |
| Red deer |  |  |  | 1 | 1 | 1 | 1 | 1 | 1 | 1 |  |  |  |  |  | 1 | 1 | 1 | 1 | 1 | 1 | 1 | 1 | 1 | 1 | 1 | 1 | 1 | 1 | 1 | 1 | 1 |  | 1 |  | 1 | 1 |  |
| Alpine marmot |  |  |  |  |  |  | 1 |  | 1 | 1 |  |  |  |  |  |  |  |  | 1 |  |  |  |  |  |  |  |  |  |  | 1 |  | 1 | 1 | 1 |  |  |  |  |
| Eurasian hare | 1 | 1 | 1 | 1 | 1 | 1 | 1 | 1 | 1 | 1 |  |  |  |  |  | 1 | 1 | 1 | 1 | 1 | 1 | 1 | 1 | 1 | 1 | 1 | 1 | 1 | 1 | 1 | 1 | 1 |  |  |  |  |  |  |
| Mountain hare |  |  |  |  |  |  | 1 | 1 | 1 | 1 |  |  |  |  |  |  | 1 |  | 1 |  |  |  |  |  |  |  |  | 1 |  | 1 |  | 1 | 1 | 1 | 1 |  |  |  |
| Red fox | 1 | 1 | 1 | 1 | 1 | 1 | 1 | 1 | 1 | 1 |  | 1 | 1 | 1 | 1 | 1 | 1 | 1 | 1 | 1 | 1 | 1 | 1 | 1 | 1 | 1 | 1 | 1 | 1 | 1 | 1 | 1 | 1 | 1 |  | 1 | 1 |  |
| European badger | 1 | 1 | 1 | 1 | 1 |  | 1 | 1 | 1 | 1 |  |  |  |  | 1 | 1 | 1 | 1 | 1 | 1 | 1 | 1 | 1 | 1 | 1 | 1 | 1 | 1 | 1 |  | 1 |  |  |  |  | 1 | 1 |  |
| Stone Martens | 1 | 1 | 1 | 1 | 1 | 1 | 1 | 1 | 1 | 1 |  | 1 | 1 | 1 | 1 | 1 | 1 | 1 | 1 | 1 | 1 | 1 | 1 | 1 | 1 | 1 | 1 | 1 | 1 | 1 | 1 | 1 | 1 | 1 |  | 1 | 1 |  |
| Pine martens |  |  |  |  |  |  |  |  |  |  |  |  |  |  |  | 1 | 1 | 1 | 1 | 1 | 1 | 1 | 1 | 1 | 1 | 1 | 1 | 1 | 1 | 1 | 1 |  |  |  |  |  |  |  |
| Capercaillie |  |  |  |  |  |  | 1 | 1 |  |  |  |  |  |  |  | 1 | 1 | 1 |  | 1 |  |  |  | 1 |  |  |  | 1 |  | 1 |  |  |  |  |  |  |  |  |
| Black Grouse |  |  |  |  |  |  | 1 | 1 |  | 1 |  |  |  |  |  |  | 1 | 1 | 1 |  |  |  |  |  |  |  |  | 1 |  | 1 |  |  |  |  |  | 1 |  |  |
| Hazel Grouse |  |  |  |  |  |  |  | 1 |  |  |  |  |  |  |  | 1 | 1 | 1 |  | 1 | 1 |  | 1 | 1 | 1 | 1 | 1 | 1 |  |  |  |  |  |  |  |  |  |  |
| Rock ptarmigan |  |  |  |  |  |  |  |  | 1 | 1 |  |  |  |  |  |  |  |  |  |  |  |  |  |  |  |  |  |  |  | 1 |  | 1 | 1 | 1 | 1 |  |  |  |
| Rock Partridge |  |  |  | 1 | 1 | 1 | 1 | 1 | 1 | 1 |  |  |  |  |  |  |  |  |  |  |  |  |  |  |  |  |  |  |  |  |  | 1 | 1 | 1 |  |  |  |  |
| Grey partridge |  |  |  | 1 | 1 | 1 | 1 |  |  |  |  |  |  |  |  |  |  |  |  |  |  |  |  |  |  |  |  |  |  |  | 1 | 1 |  |  |  |  |  |  |
| Common pheasant | 1 | 1 | 1 | 1 | 1 | 1 | 1 | 1 |  | 1 |  |  |  |  |  | 1 |  |  | 1 |  | 1 | 1 | 1 |  | 1 | 1 |  |  | 1 |  | 1 |  |  |  |  | 1 | 1 | 1 |
| Common quail |  |  |  | 1 | 1 | 1 | 1 |  |  |  |  |  |  |  |  |  |  |  |  |  |  |  |  |  |  |  |  |  |  |  | 1 |  |  |  |  | 1 | 1 | 1 |
| Common wood pigeon | 1 | 1 | 1 | 1 | 1 | 1 | 1 | 1 |  | 1 |  |  |  |  |  | 1 |  |  | 1 | 1 | 1 | 1 | 1 | 1 | 1 | 1 | 1 | 1 | 1 |  | 1 |  |  |  |  | 1 | 1 | 1 |
| Eurasian woodcock |  |  |  |  |  |  |  | 1 |  |  |  |  |  |  |  | 1 |  |  | 1 | 1 | 1 | 1 | 1 | 1 | 1 | 1 | 1 | 1 | 1 |  | 1 |  |  |  |  | 1 | 1 | 1 |
| Ducks |  |  |  |  |  |  |  |  |  |  |  |  |  |  |  |  |  |  |  |  |  |  |  |  |  |  |  |  |  |  |  |  |  |  |  | 1 | 1 | 1 |
| Eurasian coot |  |  |  |  |  |  |  |  |  |  |  |  |  |  |  |  |  |  |  |  |  |  |  |  |  |  |  |  |  |  | 1 |  |  |  |  | 1 | 1 | 1 |
| Ravens | 1 | 1 | 1 | 1 | 1 | 1 |  | 1 | 1 |  |  | 1 | 1 | 1 |  | 1 | 1 | 1 | 1 | 1 | 1 | 1 | 1 | 1 | 1 | 1 | 1 | 1 | 1 |  | 1 |  |  |  |  | 1 | 1 | 1 |
| Eurasian magpie | 1 | 1 |  | 1 | 1 | 1 | 1 | 1 |  |  | 1 | 1 | 1 | 1 | 1 | 1 |  |  | 1 |  | 1 | 1 | 1 | 1 | 1 | 1 |  |  | 1 |  | 1 |  |  |  |  | 1 | 1 | 1 |
| Eurasian jay | 1 | 1 | 1 |  |  |  |  |  |  |  |  |  |  |  |  | 1 |  | 1 | 1 | 1 | 1 | 1 | 1 | 1 | 1 | 1 | 1 | 1 | 1 |  | 1 |  |  |  |  | 1 | 1 | 1 |
| Common blackbird | 1 | 1 | 1 | 1 | 1 | 1 | 1 | 1 |  |  | 1 | 1 | 1 | 1 |  | 1 | 1 | 1 | 1 | 1 | 1 | 1 | 1 | 1 | 1 | 1 | 1 | 1 | 1 | 1 | 1 |  |  |  |  | 1 | 1 | 1 |
| Song thrush | 1 | 1 | 1 | 1 | 1 |  |  | 1 |  |  |  | 1 | 1 | 1 |  | 1 |  |  | 1 |  | 1 | 1 | 1 | 1 | 1 | 1 |  | 1 | 1 |  | 1 |  |  |  |  | 1 | 1 | 1 |
| Fieldfare | 1 | 1 | 1 | 1 | 1 |  |  | 1 |  |  |  | 1 | 1 | 1 |  | 1 |  |  | 1 |  | 1 | 1 | 1 | 1 | 1 | 1 |  | 1 | 1 |  | 1 |  |  |  |  | 1 | 1 | 1 |

**Appendix D: Correlations between the explanatory variables**

A high number of independent variables reduces the degrees of freedom of the single species models. Therefore, a PCA per driver group (i.e. 6 PCAs in total, see Table D1-D6) was computed. For the main part of the principal components, the variable with the highest loadings were used for the further analyses. For those components that contain a number of thematically quite different variables (and thus could not be proxied by a variable in terms of content), the corresponding principal components were used (e.g., H_PRD_comp1, H_S_comp3). In addition, we included the mean annual temperature and the mean annual precipitation sum, as well as the corresponding mean seasonal averages. We also included the area shares of the land use types. In total, the number of variables was reduced from the original 128 to 79 independent variables including the 9 components (see Table E1-E4). These variables and components were used for the further analyses.

Remaining multicollinearities between these 79 variables are shown in Table D7. These were removed in the model selection process and are marked in the single species models with corresponding letters (see Table E1-E4).

Table D1: Sorted pattern matrix for the 15 dimensions of potential explanatory variables for elevation zones and ecoregions emerging from principal components analysis with a varimax rotation. Item loading values >0.6 or <-0.6 are in bold, and indicators selected for further analysis are underlined. For an explanation of the abbreviations of the explanatory variable, see Appendix A.

|  | Component | | | |
| --- | --- | --- | --- | --- |
|  | 1 | 2 | 3 | 4 |
| ER_M_F | **-0.823** | -0.367 | -0.338 | -0.046 |
| A_zone | **0.798** | 0.463 | 0.108 | -0.157 |
| ER_A | **0.798** | 0.463 | 0.108 | -0.157 |
| SA_zone | **-0.788** | -0.240 | 0.221 | -0.035 |
| C_zone | **-0.761** | 0.591 | -0.218 | -0.044 |
| ER_SA_P | **0.759** | -0.085 | -0.427 | 0.066 |
| ER_C_S | **-0.726** | 0.523 | -0.126 | -0.045 |
| ER_C_F | **-0.641** | 0.478 | -0.167 | -0.091 |
| ER_C_VB | **-0.600** | 0.514 | -0.260 | 0.016 |
| M_zone | **-0.640** | **-0.649** | 0.341 | 0.132 |
| ER_SA_F | 0.565 | **-0.603** | -0.079 | -0.178 |
| ER_N (N_zone) | 0.470 | 0.568 | 0.545 | 0.315 |
| ER_M_S | -0.070 | -0.549 | -0.090 | **0.738** |
| ER_M_VB | -0.118 | -0.440 | 0.556 | -0.568 |
| **Variance explained (%)** | 41.1 | 23.3 | 9.9 | 7.4 |
| Extraction Method: Principal Component Analysis. Rotation Method: Varimax with Kaiser Normalization. | | | | |
| a. Rotation converged in 10 iterations. | | | | |

Table D2: Sorted pattern matrix for the 20 dimensions of potential explanatory climate variables emerging from principal components analysis with a varimax rotation. Item loading values >0.6 or <-0.6 are in bold, and indicators selected for further analysis are underlined. For an explanation of the abbreviations of the explanatory variable, see Appendix A.

|  | Component | | | |
| --- | --- | --- | --- | --- |
|  | 1 | 2 | 3 | 4 |
| T_10 | **0.959** | -0.125 | -0.193 | 0.105 |
| T_SU_10 | **0.959** | -0.078 | -0.195 | 0.085 |
| T_A_10 | **0.949** | -0.160 | -0.197 | 0.127 |
| T_SP_10 | **0.944** | -0.168 | -0.180 | 0.108 |
| T_W_10 | **0.934** | -0.140 | -0.211 | 0.139 |
| T_A_10_s.d. | **-0.651** | -0.446 | 0.164 | 0.479 |
| P_SP_10_s.d. | -0.074 | **0.901** | -0.072 | -0.075 |
| P_10_s.d. | -0.035 | **0.877** | 0.039 | -0.335 |
| T_SU_10_s.d. | -0.297 | **0.797** | 0.115 | -0.091 |
| T_SP_10_s.d. | -0.483 | **0.775** | -0.086 | 0.350 |
| T_W_10_s.d. | -0.005 | **-0.754** | 0.221 | -0.585 |
| P_A_10_s.d. | 0.077 | **0.753** | 0.294 | 0.393 |
| P_A_10 | 0.113 | **-0.695** | 0.624 | -0.021 |
| P_10 | -0.253 | -0.119 | **0.946** | 0.066 |
| P_SP_10 | -0.246 | 0.183 | **0.891** | -0.130 |
| P_SU_10 | -0.347 | -0.076 | **0.881** | 0.018 |
| P_W_10 | -0.380 | 0.380 | 0.592 | 0.462 |
| P_10_s.d. | -0.080 | 0.626 | 0.075 | **0.872** |
| T_10_s.d. | -0.321 | 0.062 | 0.240 | **-0.863** |
| P_S_10_s.d. | 0.311 | -0.098 | 0.260 | **0.607** |
| Variance explained (%) | 37.7 | 25.9 | 14.8 | 10.0 |
| Extraction Method: Principal Component Analysis. Rotation Method: Varimax with Kaiser Normalization. | | | | |
| a. Rotation converged in 10 iterations. | | | | |

Table D3: Sorted pattern matrix for the 16 dimensions of potential explanatory variables for landscape diversity emerging from principal components analysis with a varimax rotation. Item loading values >0.6 or <-0.6 are in bold, and indicators selected for further analysis are underlined. For an explanation of the abbreviations of the explanatory variable, see Appendix A.

|  | Component | | | | | | |
| --- | --- | --- | --- | --- | --- | --- | --- |
|  | 1 | 2 | 3 | 4 | 5 | 6 | 7 |
| H_PRD_comp1 |  |  |  |  |  |  |  |
| PRD | **0.756** | -0.033 | -0.027 | -0.160 | 0.039 | 0.199 | -0.148 |
| H_PC_lev3 | **0.686** | -0.040 | 0.187 | 0.002 | -0.243 | -0.237 | -0.006 |
| H_G_lev3 | **0.621** | 0.366 | -0.046 | 0.232 | 0.182 | 0.186 | 0.201 |
| H_AB_comp2 |  |  |  |  |  |  |  |
| H_AB_lev3 | -0.067 | **0.728** | 0.038 | 0.006 | -0.286 | -0.102 | -0.070 |
| H_F_lev3 | 0.188 | **0.720** | -0.031 | 0.183 | 0.283 | 0.034 | 0.066 |
| H_S_comp3 |  |  |  |  |  |  |  |
| H_S_lev3 | -0.033 | -0.039 | **0.759** | -0.092 | 0.126 | -0.018 | -0.043 |
| H_PC_lev2 | 0.287 | 0.060 | **0.721** | -0.061 | -0.277 | -0.070 | 0.037 |
| H_AB_lev2 | -0.029 | 0.045 | -0.070 | **0.681** | -0.097 | 0.184 | -0.167 |
| H_F_lev2 | -0.011 | 0.038 | 0.051 | -0.118 | **0.804** | -0.011 | 0.099 |
| H_G_lev2 | 0.064 | -0.043 | -0.082 | 0.063 | 0.028 | **0.848** | -0.048 |
| H_S_lev3 | -0.063 | -0.110 | -0.085 | 0.118 | 0.191 | -0.189 | **0.748** |
| H_AL_lev3 | 0.007 | 0.126 | 0.025 | -0.196 | -0.176 | 0.490 | **0.668** |
| H_NU_lev2 | -0.235 | -0.080 | 0.504 | 0.261 | 0.369 | -0.013 | -0.127 |
| H_NU_lev3 | -0.027 | 0.170 | -0.091 | 0.469 | 0.115 | -0.173 | 0.166 |
| H_AL_lev2 | -0.115 | 0.468 | -0.138 | -0.513 | 0.170 | 0.151 | -0.147 |
| SHDI | -0.429 | 0.050 | 0.168 | 0.506 | -0.338 | 0.341 | 0.043 |
| Variance explained (%) | 12.4 | 10.8 | 9.8 | 8.7 | 7.7 | 7.4 | 6.5 |
| Extraction Method: Principal Component Analysis. Rotation Method: Varimax with Kaiser Normalization. | | | | | | | |
| a. Rotation converged in 10 iterations. | | | | | | | |

Table D4: Sorted pattern matrix for the 29 dimensions of potential explanatory variables for landscape structures emerging from principal components analysis with a varimax rotation. Item loading values >0.6 or <-0.6 are in bold, and indicators selected for further analysis are underlined. For an explanation of the abbreviations of the explanatory variable, see Appendix A.

|  | Component | | | | | | | | |
| --- | --- | --- | --- | --- | --- | --- | --- | --- | --- |
|  | 1 | 2 | 3 | 4 | 5 | 6 | 7 | 8 | 9 |
| ED_comp1 |  |  |  |  |  |  |  |  |  |
| ED_v3_cl1 | **0.945** | 0.009 | -0.076 | -0.125 | -0.052 | 0.150 | -0.055 | 0.114 | 0.019 |
| ED_v3_cl2 | **0.942** | -0.081 | -0.104 | -0.094 | 0.034 | 0.197 | -0.003 | 0.103 | 0.031 |
| ED_v3_la | **0.940** | -0.080 | -0.141 | -0.053 | -0.031 | 0.200 | 0.043 | 0.122 | 0.029 |
| Sprawl | **0.742** | 0.011 | -0.077 | 0.032 | -0.074 | 0.018 | 0.182 | -0.156 | 0.122 |
| PD_v3_cl2 | **0.607** | -0.518 | -0.270 | 0.059 | -0.310 | -0.010 | -0.118 | 0.087 | -0.048 |
| PD_comp2 |  |  |  |  |  |  |  |  |  |
| PD_v3_cl1 | 0.082 | **-0.912** | -0.020 | -0.008 | 0.125 | -0.030 | 0.147 | 0.038 | 0.103 |
| PD_v3_la | 0.264 | **-0.910** | -0.165 | 0.018 | 0.011 | -0.049 | 0.115 | 0.031 | 0.078 |
| PD_v2_la | 0.132 | **-0.895** | -0.233 | -0.047 | 0.211 | -0.147 | 0.014 | 0.012 | 0.035 |
| MSI_v3_la | 0.250 | **0.682** | 0.226 | -0.098 | 0.156 | -0.027 | 0.354 | 0.309 | 0.130 |
| MSI_v2_la | 0.254 | **0.648** | 0.255 | -0.072 | 0.202 | 0.096 | 0.413 | 0.276 | 0.153 |
| MSI_v3_cl2 | 0.426 | **0.604** | 0.333 | -0.105 | 0.291 | -0.017 | 0.276 | -0.011 | 0.167 |
| MSI_v3_cl1 | 0.003 | **0.601** | -0.130 | 0.129 | -0.126 | 0.054 | 0.233 | 0.591 | 0.140 |
| TCA_comp3 |  |  |  |  |  |  |  |  |  |
| TCA_v2_la | -0.237 | 0.180 | **0.936** | -0.045 | 0.060 | 0.019 | 0.009 | 0.043 | -0.034 |
| TCA_v3_la | -0.327 | 0.183 | **0.880** | -0.058 | 0.202 | -0.057 | -0.013 | 0.078 | -0.036 |
| TCA_v3_cl1 | 0.165 | 0.212 | **0.841** | -0.079 | -0.286 | 0.065 | 0.042 | -0.020 | -0.020 |
| TCA_v3_cl2 | -0.478 | 0.130 | **0.701** | -0.039 | 0.379 | -0.098 | -0.038 | 0.106 | -0.038 |
| ENN_comp4 |  |  |  |  |  |  |  |  |  |
| ENN_v3_la | -0.083 | 0.010 | -0.124 | **0.961** | -0.072 | -0.029 | 0.016 | -0.058 | 0.058 |
| ENN_v3_cl1 | -0.030 | -0.029 | -0.058 | **0.943** | -0.022 | 0.012 | 0.050 | 0.203 | 0.047 |
| ENN_v2_la | -0.072 | -0.019 | 0.015 | **0.863** | -0.028 | 0.113 | -0.006 | -0.224 | -0.112 |
| S_52 | 0.391 | 0.185 | -0.241 | 0.015 | **-0.775** | -0.240 | 0.077 | -0.080 | -0.005 |
| ED_v2_la | 0.553 | -0.056 | -0.193 | -0.169 | **0.656** | -0.062 | -0.054 | 0.203 | -0.023 |
| S_51 | 0.205 | 0.137 | 0.003 | -0.068 | 0.234 | **0.812** | -0.082 | 0.051 | 0.157 |
| S_40 | -0.028 | 0.002 | -0.001 | -0.028 | 0.123 | -0.094 | **-0.834** | -0.071 | -0.018 |
| ENN_v3_cl2 | -0.111 | 0.043 | -0.244 | 0.140 | -0.255 | -0.134 | -0.022 | **-0.718** | 0.159 |
| S_41 | -0.102 | 0.091 | 0.150 | -0.062 | -0.112 | -0.095 | -0.228 | 0.131 | **-0.741** |
| S_42 | 0.023 | 0.079 | 0.139 | -0.103 | -0.220 | 0.147 | -0.401 | 0.143 | **0.639** |
| S_10 | 0.138 | 0.138 | 0.085 | 0.218 | -0.070 | 0.592 | 0.410 | 0.025 | -0.060 |
| S_30 | -0.478 | 0.036 | 0.114 | -0.129 | 0.576 | -0.556 | -0.191 | -0.050 | -0.108 |
| S_20 | 0.226 | 0.003 | -0.123 | -0.008 | -0.164 | 0.502 | -0.109 | 0.378 | 0.320 |
| Variance explained (%) | 23.6 | 18.7 | 11.5 | 7.6 | 6.1 | 5.5 | 4.5 | 3.6 | 3.5 |
| Extraction Method: Principal Component Analysis. Rotation Method: Varimax with Kaiser Normalization. | | | | | | | | | |
| a. Rotation converged in 10 iterations. | | | | | | | | | |

Table D5: Sorted pattern matrix for the 24 dimensions of potential explanatory variables for LULC types emerging from principal components analysis with a varimax rotation. Item loading values >0.6 or <-0.6 are in bold, and indicators selected for further analysis are underlined. For an explanation of the abbreviations of the explanatory variable, see Appendix A.

|  | Component | | | | | | | | |
| --- | --- | --- | --- | --- | --- | --- | --- | --- | --- |
|  | 1 | 2 | 3 | 4 | 5 | 6 | 7 | 8 | 9 |
| SH_322000 | **0.858** | 0.101 | -0.248 | 0.312 | -0.016 | 0.224 | 0.048 | -0.060 | 0.109 |
| SH_222200 | **0.830** | 0.118 | -0.312 | 0.333 | 0.038 | 0.163 | 0.006 | -0.075 | 0.111 |
| SH_322100 | **0.805** | 0.092 | -0.191 | 0.371 | -0.208 | 0.259 | -0.001 | -0.032 | 0.033 |
| SH_314000 | **0.799** | 0.021 | 0.237 | -0.162 | 0.052 | -0.154 | 0.078 | 0.091 | 0.148 |
| SH_313000 | **0.682** | -0.051 | 0.276 | -0.330 | 0.333 | -0.188 | -0.126 | 0.147 | -0.031 |
| SH_310000 | 0.588 | 0.239 | 0.386 | 0.212 | -0.161 | -0.228 | -0.026 | 0.067 | -0.170 |
| SH_212000 | 0.547 | 0.102 | -0.091 | 0.274 | -0.073 | -0.029 | -0.350 | -0.239 | -0.252 |
| SH_133000 | -0.253 | **0.699** | 0.124 | 0.115 | 0.222 | 0.251 | 0.024 | 0.078 | -0.093 |
| SH_234000 | -0.390 | **0.613** | 0.098 | 0.080 | 0.145 | 0.163 | -0.212 | -0.039 | 0.189 |
| SH_111000 | -0.083 | **0.612** | 0.380 | 0.103 | 0.179 | 0.250 | 0.007 | 0.091 | -0.194 |
| SH_232000 | -0.439 | 0.591 | -0.029 | 0.119 | 0.039 | -0.108 | -0.113 | -0.038 | 0.340 |
| SH_221000 | 0.118 | -0.567 | 0.187 | -0.175 | -0.104 | 0.426 | 0.069 | 0.167 | -0.140 |
| SH_222100 | -0.305 | -0.286 | **-0.632** | 0.102 | 0.006 | -0.187 | -0.222 | -0.079 | -0.234 |
| SH_311000 | 0.412 | 0.122 | 0.548 | -0.077 | -0.167 | -0.361 | -0.139 | -0.023 | -0.206 |
| SH_413000 | -0.199 | -0.378 | 0.283 | **0.606** | 0.369 | 0.005 | 0.090 | 0.072 | -0.022 |
| SH_122100 | -0.140 | -0.361 | 0.189 | 0.498 | 0.399 | 0.018 | -0.123 | 0.307 | -0.071 |
| SH_231000 | -0.283 | -0.341 | 0.227 | 0.458 | 0.249 | -0.027 | 0.275 | -0.179 | -0.030 |
| SH_211000 | 0.356 | -0.057 | -0.164 | -0.400 | **0.686** | -0.099 | 0.001 | -0.018 | -0.034 |
| SH_322100 | 0.374 | 0.055 | -0.242 | -0.119 | **0.622** | -0.060 | 0.171 | -0.103 | 0.274 |
| SH_452000 | 0.054 | -0.160 | 0.006 | -0.137 | -0.135 | 0.530 | -0.117 | 0.494 | 0.218 |
| SH_113000 | -0.185 | -0.211 | 0.076 | 0.295 | -0.083 | -0.396 | -0.341 | 0.023 | 0.395 |
| SH_451000 | -0.142 | 0.394 | -0.376 | 0.088 | 0.045 | -0.144 | 0.434 | 0.155 | -0.428 |
| SH_131000 | 0.136 | 0.158 | -0.154 | 0.169 | -0.267 | -0.454 | 0.459 | 0.488 | 0.219 |
| Variance explained (%) | 0.136 | 0.158 | -0.154 | 0.169 | -0.267 | -0.454 | 0.459 | 0.488 | 0.219 |
| Extraction Method: Principal Component Analysis. Rotation Method: Varimax with Kaiser Normalization. | | | | | | | | | |
| a. Rotation converged in 10 iterations. | | | | | | | | | |

Table D6: Sorted pattern matrix for the 24 dimensions of potential explanatory variables for land use emerging from principal components analysis with a varimax rotation. Item loading values >0.6 or <-0.6 are in bold, and indicators selected for further analysis are underlined. For an explanation of the abbreviations of the explanatory variable, see Appendix A.

|  | Component | | | | | |
| --- | --- | --- | --- | --- | --- | --- |
|  | 1 | 2 | 3 | 4 | 5 | 6 |
| LU_orchard_comp1 |  |  |  |  |  |  |
| Pest_total | **0.858** | -0.285 | 0.096 | 0.226 | -0.103 | -0.018 |
| LU_orchard | **0.851** | -0.117 | -0.019 | 0.215 | -0.163 | 0.018 |
| Pest_orchard | **0.816** | -0.276 | 0.090 | 0.260 | -0.206 | -0.026 |
| Pest_density | **0.762** | -0.169 | 0.036 | 0.226 | -0.235 | -0.073 |
| LU_settl* | **0.685** | 0.183 | 0.068 | 0.126 | -0.371 | 0.172 |
| CUT_mean | 0.207 | **0.698** | -0.038 | 0.007 | -0.339 | -0.193 |
| LU_gras_e | -0.563 | **-0.672** | 0.065 | 0.169 | -0.076 | 0.083 |
| LU_pasture | -0.565 | **-0.667** | 0.069 | 0.209 | -0.103 | 0.084 |
| LU_forest | 0.218 | **0.657** | -0.083 | -0.178 | 0.246 | -0.029 |
| LU_gras_i | -0.247 | **0.615** | -0.026 | 0.384 | 0.005 | 0.198 |
| LU_LSU_comp2 |  |  |  |  |  |  |
| LI_LSU | -0.162 | 0.210 | **0.926** | -0.004 | -0.007 | 0.014 |
| LI_cattle | -0.139 | 0.223 | **0.914** | -0.077 | -0.023 | 0.018 |
| LI_horse | -0.079 | 0.255 | **0.673** | 0.124 | 0.311 | 0.196 |
| Pest_vine | 0.584 | -0.217 | 0.075 | -0.047 | 0.483 | 0.041 |
| Hail_net | 0.594 | -0.223 | 0.074 | 0.138 | 0.116 | 0.187 |
| LI_LSD | 0.590 | -0.143 | 0.230 | -0.178 | 0.235 | -0.075 |
| d2n | 0.258 | 0.586 | -0.184 | 0.480 | -0.007 | 0.065 |
| Pest_arable | 0.156 | 0.364 | 0.225 | -0.174 | -0.272 | -0.233 |
| Tourism | 0.351 | -0.023 | 0.488 | -0.372 | 0.007 | 0.217 |
| LI_sheep | -0.295 | -0.186 | 0.465 | **0.661** | 0.014 | -0.028 |
| LI_goat | -0.225 | -0.029 | 0.193 | 0.539 | 0.163 | -0.322 |
| LU_arable | -0.172 | 0.383 | -0.321 | 0.484 | 0.376 | -0.038 |
| LU_vine | 0.567 | -0.090 | -0.112 | -0.030 | **0.651** | -0.072 |
| LU_aband | -0.046 | 0.152 | -0.216 | 0.061 | -0.047 | **0.778** |
| Variance explained (%) |  |  |  |  |  |  |
| Extraction Method: Principal Component Analysis. Rotation Method: Varimax with Kaiser Normalization. | | | | | | |
| a. Rotation converged in 10 iterations.  * For the sake of completeness this variable was included for the further analysis. | | | | | | |

Table D7: Sorted pattern matrix for the 24 dimensions of potential explanatory variables emerging from principal components analysis with a varimax rotation. Item loading values >0.5 or <-0.5 are shown in bold. For an explanation of the abbreviations of the explanatory variable, see Appendix A.

|  | Component | | | | | | | | | | | | | | | | | | | | | | | |
| --- | --- | --- | --- | --- | --- | --- | --- | --- | --- | --- | --- | --- | --- | --- | --- | --- | --- | --- | --- | --- | --- | --- | --- | --- |
|  | 1 | 2 | 3 | 4 | 5 | 6 | 7 | 8 | 9 | 10 | 11 | 12 | 13 | 14 | 15 | 16 | 17 | 18 | 19 | 20 | 21 | 22 | 23 | 24 |
| LU_forest | **0.82** | 0.07 | -0.15 | 0.09 | 0.04 | 0.01 | 0.21 | 0.10 | -0.15 | 0.07 | -0.06 | -0.04 | 0.09 | 0.09 | 0.01 | -0.10 | -0.11 | 0.17 | 0.00 | 0.01 | -0.04 | 0.09 | 0.02 | 0.06 |
| S_52 | **0.79** | 0.11 | -0.14 | 0.05 | -0.25 | -0.07 | 0.16 | 0.15 | -0.17 | 0.09 | -0.08 | -0.04 | -0.01 | 0.09 | -0.01 | -0.11 | -0.15 | 0.21 | -0.04 | 0.04 | -0.06 | 0.12 | 0.01 | 0.07 |
| S_30 | **-0.79** | -0.14 | -0.03 | -0.02 | -0.39 | -0.14 | 0.11 | 0.00 | -0.11 | -0.16 | -0.05 | -0.12 | -0.06 | -0.12 | 0.09 | 0.12 | -0.04 | -0.10 | 0.03 | 0.08 | -0.04 | -0.02 | -0.01 | -0.01 |
| ER_N | **-0.77** | -0.01 | -0.08 | -0.02 | -0.09 | -0.04 | 0.16 | 0.01 | -0.07 | 0.16 | -0.02 | -0.02 | 0.03 | 0.01 | 0.02 | -0.27 | 0.07 | 0.14 | -0.08 | -0.18 | -0.05 | 0.13 | -0.01 | 0.09 |
| SH_311000 | **-0.76** | 0.00 | 0.00 | -0.13 | -0.09 | -0.06 | 0.14 | -0.03 | -0.08 | 0.15 | -0.04 | -0.03 | -0.02 | 0.05 | 0.00 | -0.22 | -0.06 | 0.09 | -0.09 | -0.10 | -0.05 | 0.09 | -0.03 | 0.00 |
| SH_310000 | **-0.74** | -0.01 | 0.08 | 0.00 | -0.12 | -0.10 | 0.09 | -0.02 | -0.12 | 0.17 | -0.07 | -0.02 | 0.03 | 0.01 | -0.11 | 0.00 | -0.04 | 0.11 | 0.12 | 0.24 | -0.01 | 0.14 | 0.02 | 0.13 |
| ER_M_F | **0.71** | 0.02 | -0.18 | -0.08 | -0.08 | 0.16 | -0.09 | 0.04 | 0.13 | 0.31 | -0.10 | -0.07 | 0.01 | 0.07 | -0.35 | -0.21 | -0.02 | 0.08 | -0.01 | -0.05 | -0.05 | 0.09 | -0.04 | 0.03 |
| SHDI | **-0.70** | -0.17 | 0.15 | -0.07 | -0.16 | -0.03 | 0.17 | -0.01 | 0.10 | -0.19 | 0.12 | 0.00 | -0.15 | -0.11 | 0.01 | 0.23 | 0.09 | -0.30 | 0.10 | 0.07 | 0.08 | -0.16 | 0.00 | -0.07 |
| T_10 | **0.60** | 0.37 | -0.16 | 0.16 | -0.03 | 0.23 | -0.21 | -0.03 | 0.02 | 0.13 | 0.10 | 0.36 | 0.05 | -0.03 | -0.01 | -0.12 | -0.07 | 0.11 | -0.05 | 0.01 | -0.06 | 0.09 | -0.01 | 0.03 |
| CUT_mean | **0.51** | -0.41 | -0.12 | 0.08 | -0.23 | 0.03 | -0.23 | 0.03 | 0.13 | 0.33 | 0.02 | 0.23 | 0.01 | 0.11 | 0.00 | -0.11 | 0.05 | 0.18 | 0.03 | 0.15 | 0.03 | 0.00 | 0.07 | 0.00 |
| P_W10 | **-0.50** | -0.06 | -0.31 | -0.19 | -0.24 | -0.37 | 0.05 | 0.01 | -0.04 | -0.03 | -0.06 | -0.13 | -0.06 | -0.03 | -0.03 | 0.16 | 0.14 | 0.15 | 0.19 | 0.05 | 0.16 | -0.21 | 0.17 | -0.05 |
| ED_comp1 | 0.48 | -0.08 | 0.04 | 0.20 | 0.17 | -0.15 | -0.01 | -0.07 | -0.15 | 0.05 | 0.27 | 0.08 | -0.05 | 0.35 | 0.27 | -0.01 | 0.22 | 0.27 | 0.09 | 0.03 | 0.02 | 0.07 | 0.17 | 0.13 |
| P_10 | -0.45 | -0.12 | -0.41 | 0.29 | -0.19 | -0.28 | 0.18 | -0.01 | -0.19 | -0.05 | -0.11 | -0.10 | -0.05 | 0.10 | -0.11 | 0.31 | -0.08 | 0.02 | 0.05 | 0.16 | 0.09 | -0.09 | -0.01 | -0.08 |
| SH_322000 | -0.39 | -0.16 | -0.10 | 0.32 | -0.24 | -0.15 | 0.19 | -0.08 | -0.03 | 0.05 | -0.01 | -0.10 | 0.08 | -0.02 | 0.02 | 0.15 | 0.36 | -0.09 | 0.26 | 0.32 | 0.07 | -0.20 | 0.04 | -0.07 |
| SH_133000 | 0.03 | **0.89** | -0.01 | 0.00 | 0.02 | -0.07 | -0.07 | 0.00 | -0.01 | -0.04 | -0.02 | 0.04 | 0.08 | 0.02 | 0.00 | -0.03 | -0.02 | -0.09 | -0.02 | 0.01 | -0.01 | -0.05 | -0.03 | -0.02 |
| Pest_vine | 0.10 | **0.82** | -0.02 | 0.06 | -0.04 | -0.04 | 0.04 | -0.02 | 0.29 | 0.06 | 0.02 | -0.05 | -0.10 | -0.04 | -0.04 | -0.02 | 0.01 | 0.13 | 0.00 | 0.00 | 0.00 | 0.00 | -0.01 | -0.04 |
| LU_vine | 0.19 | **0.80** | -0.14 | -0.02 | -0.10 | 0.11 | -0.01 | -0.07 | 0.07 | 0.17 | 0.03 | 0.13 | 0.02 | -0.10 | -0.18 | -0.15 | 0.11 | -0.11 | -0.05 | -0.06 | -0.03 | 0.06 | -0.01 | 0.04 |
| LI_LSD | 0.07 | **0.59** | 0.02 | 0.24 | -0.09 | -0.17 | 0.02 | -0.05 | 0.32 | -0.01 | 0.12 | -0.07 | 0.12 | 0.08 | 0.06 | -0.04 | 0.03 | 0.01 | 0.23 | 0.26 | 0.03 | -0.09 | 0.11 | -0.05 |
| TCA_comp3 | -0.14 | -0.05 | **0.85** | 0.04 | 0.01 | -0.17 | 0.00 | -0.06 | -0.06 | -0.02 | -0.05 | 0.06 | 0.05 | -0.13 | -0.06 | -0.20 | 0.11 | 0.02 | 0.16 | -0.01 | 0.02 | -0.02 | 0.04 | 0.02 |
| LU_LSU_comp2 | 0.09 | 0.03 | **0.80** | 0.28 | 0.02 | 0.03 | 0.06 | 0.00 | -0.04 | 0.06 | 0.05 | 0.00 | 0.13 | 0.11 | 0.10 | 0.09 | 0.12 | 0.29 | 0.05 | -0.05 | 0.06 | -0.03 | 0.11 | 0.01 |
| Adj_hunt_area | -0.36 | -0.03 | **0.80** | -0.02 | -0.01 | -0.09 | 0.20 | -0.09 | -0.08 | 0.01 | -0.06 | 0.00 | 0.01 | -0.15 | -0.10 | -0.12 | 0.09 | -0.02 | 0.18 | -0.03 | 0.01 | -0.01 | 0.04 | 0.02 |
| LI_sheep | -0.16 | -0.16 | **0.73** | -0.23 | 0.10 | 0.15 | 0.05 | -0.05 | 0.19 | 0.01 | -0.02 | -0.20 | -0.05 | 0.02 | 0.06 | 0.11 | -0.05 | -0.20 | -0.12 | -0.04 | -0.04 | 0.02 | -0.03 | -0.01 |
| LI_goat | 0.00 | -0.05 | 0.46 | -0.20 | -0.19 | 0.03 | 0.07 | -0.10 | -0.02 | 0.04 | 0.04 | 0.01 | 0.02 | 0.29 | 0.15 | -0.01 | -0.01 | -0.37 | -0.18 | 0.37 | -0.05 | 0.07 | 0.01 | 0.07 |
| Hunt_manag | 0.06 | 0.01 | -0.06 | **0.81** | -0.10 | -0.32 | -0.11 | -0.12 | 0.17 | 0.08 | 0.07 | -0.04 | -0.03 | -0.06 | -0.02 | -0.03 | 0.12 | 0.19 | 0.05 | 0.04 | 0.06 | 0.02 | 0.03 | 0.02 |
| P_10_std | -0.14 | -0.02 | 0.03 | **-0.78** | -0.06 | 0.11 | 0.11 | 0.03 | -0.08 | 0.16 | -0.05 | 0.05 | -0.06 | 0.05 | 0.04 | 0.15 | -0.03 | 0.00 | -0.09 | 0.13 | 0.06 | -0.11 | -0.07 | -0.17 |
| T_SP10_std | -0.27 | -0.09 | -0.02 | **-0.74** | 0.02 | -0.13 | 0.09 | 0.07 | -0.03 | -0.08 | -0.02 | -0.15 | 0.05 | -0.01 | 0.03 | 0.01 | 0.18 | 0.14 | 0.22 | -0.06 | 0.09 | -0.19 | 0.16 | -0.04 |
| Tourism | 0.00 | 0.21 | 0.24 | **0.60** | 0.08 | -0.22 | 0.11 | 0.00 | 0.10 | 0.04 | -0.04 | 0.13 | 0.00 | 0.09 | -0.03 | 0.03 | 0.17 | 0.11 | -0.01 | -0.02 | 0.28 | -0.23 | 0.02 | -0.12 |
| Prot_status | 0.00 | -0.03 | -0.01 | 0.42 | -0.02 | -0.02 | -0.03 | -0.02 | 0.08 | 0.05 | 0.02 | -0.09 | -0.02 | -0.04 | -0.06 | -0.01 | 0.01 | -0.07 | -0.01 | 0.06 | 0.07 | 0.42 | 0.13 | -0.21 |
| H_F_lev2 | 0.13 | -0.09 | -0.06 | 0.02 | **0.82** | 0.01 | 0.06 | -0.03 | -0.07 | 0.03 | -0.01 | -0.13 | 0.16 | 0.02 | -0.06 | -0.03 | 0.00 | 0.06 | 0.03 | -0.06 | -0.01 | 0.02 | 0.00 | 0.03 |
| S_51 | 0.09 | -0.14 | 0.09 | -0.08 | **0.79** | 0.23 | 0.11 | -0.11 | 0.12 | 0.02 | 0.04 | -0.09 | 0.15 | 0.09 | 0.03 | 0.13 | 0.14 | -0.10 | 0.02 | -0.08 | 0.01 | -0.01 | 0.00 | -0.01 |
| S_10 | 0.20 | 0.19 | 0.10 | 0.04 | **0.66** | 0.03 | 0.07 | -0.05 | 0.11 | 0.06 | 0.14 | 0.38 | -0.08 | 0.06 | -0.20 | -0.08 | -0.04 | 0.01 | 0.10 | -0.06 | 0.12 | -0.13 | -0.01 | -0.10 |
| ER_M_S | 0.26 | -0.15 | 0.04 | -0.02 | **0.54** | -0.02 | -0.07 | 0.03 | -0.01 | 0.21 | 0.35 | -0.16 | -0.02 | 0.04 | 0.49 | 0.05 | 0.09 | 0.02 | -0.09 | 0.16 | -0.03 | 0.08 | 0.05 | 0.12 |
| H_AL_lev2 | 0.13 | -0.03 | -0.08 | -0.12 | 0.10 | **0.84** | 0.09 | 0.11 | -0.08 | -0.08 | -0.08 | 0.07 | -0.02 | 0.04 | 0.10 | 0.00 | -0.07 | 0.06 | 0.02 | 0.01 | -0.04 | -0.03 | 0.02 | 0.03 |
| LU_arable | 0.20 | -0.04 | -0.04 | -0.28 | 0.01 | **0.79** | -0.01 | 0.02 | -0.14 | 0.05 | -0.08 | 0.18 | -0.05 | 0.04 | -0.11 | -0.01 | -0.07 | -0.13 | -0.11 | 0.02 | -0.05 | 0.00 | -0.09 | -0.02 |
| H_AB_comp2 | -0.04 | -0.08 | 0.01 | 0.03 | 0.00 | **0.54** | 0.02 | 0.11 | -0.02 | -0.10 | -0.01 | -0.02 | 0.32 | -0.15 | 0.34 | -0.04 | 0.09 | 0.07 | 0.36 | 0.05 | -0.01 | -0.07 | 0.09 | 0.06 |
| S_20 | 0.34 | 0.23 | 0.08 | -0.10 | 0.15 | 0.49 | -0.07 | -0.14 | 0.05 | 0.21 | 0.22 | -0.06 | 0.12 | 0.15 | -0.01 | 0.03 | 0.29 | -0.13 | -0.04 | -0.19 | 0.08 | -0.01 | 0.04 | 0.01 |
| LU_gras_i | 0.23 | -0.20 | 0.05 | -0.05 | 0.26 | 0.41 | -0.26 | 0.07 | -0.16 | 0.14 | 0.20 | -0.03 | -0.10 | 0.32 | 0.34 | 0.28 | -0.07 | 0.14 | -0.17 | 0.06 | -0.03 | 0.02 | -0.07 | -0.05 |
| PD_comp2 | 0.02 | 0.10 | -0.03 | 0.01 | 0.08 | -0.02 | **0.92** | -0.04 | -0.08 | 0.09 | -0.01 | 0.00 | 0.07 | -0.04 | 0.04 | -0.04 | 0.00 | 0.02 | 0.02 | -0.04 | -0.01 | 0.02 | 0.02 | 0.03 |
| H_PRD_comp1 | 0.24 | 0.13 | -0.22 | 0.05 | -0.04 | -0.08 | **-0.83** | 0.06 | -0.06 | 0.16 | -0.04 | -0.02 | 0.13 | 0.02 | 0.02 | 0.04 | -0.01 | 0.03 | 0.04 | -0.01 | -0.05 | 0.05 | 0.00 | 0.03 |
| Hunter_dens | 0.20 | 0.12 | -0.12 | 0.30 | -0.04 | -0.17 | **-0.72** | -0.10 | 0.31 | 0.12 | 0.08 | 0.08 | 0.05 | -0.01 | -0.07 | -0.09 | 0.09 | 0.22 | 0.01 | 0.02 | -0.01 | 0.03 | 0.05 | 0.05 |
| ER_M_VB | 0.13 | -0.17 | 0.09 | 0.05 | -0.37 | 0.20 | -0.48 | 0.28 | -0.06 | -0.07 | -0.16 | -0.23 | -0.06 | 0.34 | 0.14 | 0.08 | -0.18 | 0.23 | 0.03 | -0.14 | 0.00 | -0.04 | -0.01 | -0.06 |
| LU_aband | 0.06 | -0.01 | -0.05 | -0.06 | -0.03 | 0.05 | -0.04 | **0.95** | 0.00 | 0.05 | 0.00 | -0.01 | -0.03 | 0.00 | 0.05 | 0.05 | -0.08 | -0.01 | -0.01 | 0.01 | -0.01 | 0.02 | 0.00 | 0.01 |
| SH_413000 | 0.04 | -0.02 | 0.00 | -0.06 | -0.04 | 0.08 | -0.03 | **0.92** | 0.01 | 0.05 | -0.02 | 0.02 | -0.01 | -0.04 | 0.14 | 0.09 | -0.02 | 0.02 | 0.02 | 0.01 | -0.02 | 0.01 | 0.00 | 0.01 |
| SH_122100 | 0.07 | -0.06 | -0.08 | -0.06 | -0.08 | -0.09 | 0.04 | **0.80** | -0.03 | -0.15 | -0.04 | -0.03 | -0.01 | 0.07 | -0.22 | -0.10 | 0.03 | -0.09 | -0.03 | -0.01 | -0.01 | 0.01 | -0.03 | -0.02 |
| SH_231000 | 0.03 | -0.05 | -0.06 | 0.06 | 0.05 | 0.20 | -0.47 | **0.55** | 0.03 | 0.10 | 0.03 | 0.04 | -0.03 | -0.04 | 0.28 | -0.01 | -0.12 | -0.02 | -0.05 | -0.02 | 0.03 | -0.07 | 0.04 | -0.01 |
| LU_orchard_comp1 | 0.01 | 0.23 | 0.03 | 0.05 | 0.03 | -0.13 | -0.13 | -0.01 | **0.87** | 0.06 | 0.07 | 0.18 | -0.08 | 0.03 | -0.05 | -0.02 | -0.05 | 0.03 | -0.03 | -0.01 | 0.03 | 0.01 | 0.01 | 0.01 |
| LU_settl | 0.19 | 0.09 | -0.07 | 0.24 | 0.07 | -0.11 | -0.17 | 0.07 | **0.63** | -0.05 | 0.14 | 0.39 | 0.01 | 0.20 | -0.03 | -0.07 | -0.15 | 0.21 | 0.01 | 0.03 | 0.04 | 0.00 | -0.02 | -0.02 |
| Hail_net | 0.03 | 0.37 | 0.00 | 0.27 | 0.04 | -0.03 | 0.03 | -0.02 | **0.59** | 0.09 | -0.09 | 0.06 | -0.10 | -0.06 | 0.00 | -0.02 | 0.00 | -0.08 | -0.09 | -0.08 | -0.07 | 0.11 | -0.03 | 0.08 |
| SH_452000 | -0.01 | -0.05 | -0.19 | 0.06 | -0.15 | 0.10 | 0.05 | -0.05 | 0.02 | **-0.76** | -0.02 | -0.08 | -0.03 | -0.04 | -0.04 | -0.10 | 0.16 | 0.10 | -0.03 | -0.06 | 0.03 | -0.01 | 0.02 | -0.02 |
| LU_pasture | **-0.51** | -0.13 | 0.28 | -0.27 | -0.04 | -0.04 | 0.03 | 0.01 | -0.12 | **-0.54** | 0.09 | -0.10 | -0.15 | -0.19 | 0.06 | 0.04 | -0.07 | -0.16 | -0.09 | -0.05 | -0.08 | 0.13 | -0.03 | 0.07 |
| LU_gras_e | **-0.50** | -0.11 | 0.26 | -0.27 | -0.08 | -0.06 | 0.15 | 0.03 | -0.14 | **-0.54** | 0.09 | -0.10 | -0.11 | -0.18 | 0.08 | 0.04 | -0.09 | -0.16 | -0.06 | -0.07 | -0.07 | 0.14 | -0.04 | 0.07 |
| ER_SA_F | 0.04 | -0.34 | 0.00 | 0.06 | 0.12 | -0.25 | 0.00 | 0.09 | -0.26 | **-0.53** | -0.09 | -0.30 | -0.24 | 0.07 | 0.35 | 0.04 | -0.10 | -0.09 | 0.04 | 0.06 | 0.01 | -0.06 | 0.00 | -0.01 |
| H_S_lev3 | 0.06 | -0.03 | -0.06 | 0.05 | 0.05 | -0.04 | 0.01 | -0.02 | -0.02 | -0.04 | **0.89** | 0.03 | 0.01 | 0.02 | -0.03 | -0.05 | -0.06 | 0.09 | -0.02 | 0.04 | -0.05 | 0.05 | -0.06 | 0.00 |
| H_S_comp3 | -0.10 | 0.10 | 0.02 | 0.02 | 0.06 | -0.02 | -0.02 | -0.03 | 0.12 | 0.03 | **0.89** | -0.08 | 0.00 | -0.07 | 0.01 | -0.04 | 0.02 | -0.04 | -0.03 | -0.24 | 0.04 | -0.06 | 0.04 | -0.02 |
| ENN_comp4 | 0.05 | -0.05 | -0.03 | 0.01 | -0.04 | 0.09 | 0.01 | 0.03 | 0.21 | 0.10 | -0.06 | **0.77** | -0.08 | 0.00 | 0.03 | -0.07 | -0.08 | -0.10 | 0.00 | 0.03 | -0.03 | 0.06 | 0.00 | 0.01 |
| SH_232000 | 0.21 | **0.51** | -0.02 | 0.01 | -0.12 | 0.22 | 0.02 | -0.07 | 0.16 | 0.15 | -0.05 | **0.61** | 0.07 | 0.04 | -0.15 | -0.02 | 0.01 | 0.16 | -0.01 | -0.06 | -0.03 | 0.06 | 0.04 | 0.03 |
| S_42 | 0.10 | -0.07 | 0.17 | 0.05 | 0.13 | 0.14 | 0.02 | 0.03 | 0.01 | 0.07 | 0.01 | -0.15 | **0.83** | -0.05 | 0.09 | 0.02 | 0.00 | 0.07 | -0.02 | 0.03 | 0.02 | -0.01 | -0.01 | -0.06 |
| SH_451000 | 0.15 | 0.18 | 0.01 | -0.03 | 0.16 | -0.10 | -0.07 | -0.08 | -0.14 | 0.08 | 0.01 | 0.06 | **0.78** | 0.13 | 0.09 | -0.06 | 0.09 | -0.13 | -0.01 | 0.04 | 0.00 | -0.01 | 0.04 | 0.04 |
| SH_131000 | -0.15 | -0.03 | -0.19 | -0.05 | -0.05 | -0.07 | 0.03 | -0.07 | -0.05 | 0.01 | -0.06 | 0.11 | **0.53** | -0.08 | -0.17 | **0.51** | -0.13 | 0.11 | -0.01 | -0.01 | -0.05 | 0.08 | 0.05 | 0.12 |
| SH_222100 | **0.52** | -0.23 | -0.15 | 0.02 | 0.10 | -0.11 | 0.13 | 0.06 | -0.05 | 0.09 | -0.07 | -0.13 | 0.03 | **0.68** | -0.03 | -0.06 | -0.07 | 0.09 | -0.01 | 0.00 | -0.04 | 0.02 | -0.01 | 0.01 |
| d2n | 0.45 | 0.01 | -0.06 | -0.14 | 0.18 | 0.21 | -0.27 | 0.02 | 0.22 | 0.05 | -0.05 | 0.13 | -0.05 | **0.63** | 0.04 | -0.01 | -0.16 | 0.00 | -0.05 | 0.03 | -0.08 | 0.05 | -0.07 | 0.00 |
| SH_221000 | 0.16 | -0.27 | 0.10 | 0.07 | 0.02 | -0.21 | 0.13 | 0.11 | -0.24 | -0.28 | 0.02 | -0.31 | -0.31 | -0.49 | 0.23 | 0.04 | -0.14 | 0.08 | -0.03 | 0.07 | -0.02 | 0.02 | 0.03 | 0.07 |
| S_40 | -0.15 | -0.10 | 0.00 | -0.12 | -0.17 | 0.13 | 0.02 | 0.07 | -0.02 | -0.08 | -0.08 | -0.02 | 0.15 | -0.01 | **0.78** | -0.04 | 0.03 | -0.12 | 0.06 | -0.05 | 0.00 | 0.01 | -0.04 | -0.04 |
| H_G_lev2 | -0.06 | -0.13 | -0.05 | -0.18 | 0.08 | 0.05 | -0.07 | 0.07 | -0.04 | 0.04 | -0.08 | -0.12 | -0.03 | 0.01 | 0.04 | **0.79** | -0.03 | -0.05 | -0.10 | 0.00 | -0.01 | 0.06 | -0.03 | -0.02 |
| H_AB_lev2 | -0.10 | -0.11 | -0.12 | 0.23 | -0.21 | -0.14 | 0.12 | 0.04 | 0.00 | 0.05 | -0.04 | -0.02 | 0.04 | -0.12 | 0.01 | 0.46 | 0.40 | 0.00 | 0.19 | 0.00 | -0.08 | -0.11 | -0.09 | -0.06 |
| SH_113000 | 0.03 | -0.11 | -0.23 | -0.05 | -0.16 | 0.12 | 0.04 | 0.18 | 0.09 | 0.01 | 0.14 | 0.15 | -0.08 | 0.16 | -0.13 | 0.07 | **-0.66** | -0.01 | 0.00 | 0.09 | -0.01 | -0.11 | -0.06 | -0.06 |
| ENN_v3_cl2 | 0.23 | -0.15 | -0.25 | 0.00 | -0.15 | -0.17 | 0.02 | 0.01 | 0.08 | 0.25 | -0.21 | -0.02 | 0.03 | -0.21 | 0.18 | 0.01 | **-0.55** | -0.10 | -0.06 | -0.06 | 0.01 | -0.08 | -0.03 | -0.06 |
| Pest_arable | 0.20 | -0.06 | 0.05 | 0.02 | -0.04 | -0.03 | -0.09 | -0.09 | 0.06 | -0.01 | 0.05 | -0.03 | -0.01 | 0.05 | -0.08 | -0.02 | 0.03 | **0.73** | -0.07 | 0.07 | 0.03 | -0.02 | -0.01 | -0.05 |
| H_NU_lev3 | -0.04 | -0.01 | 0.15 | -0.02 | 0.05 | -0.03 | 0.00 | -0.02 | -0.05 | 0.03 | -0.05 | 0.02 | -0.05 | -0.02 | 0.04 | -0.07 | 0.05 | -0.06 | **0.79** | 0.03 | -0.04 | 0.09 | -0.04 | 0.04 |
| S_41 | -0.04 | -0.16 | 0.07 | 0.01 | -0.12 | -0.15 | 0.14 | 0.13 | 0.00 | -0.21 | -0.13 | 0.06 | -0.09 | -0.22 | 0.15 | -0.30 | 0.35 | -0.01 | -0.38 | 0.11 | 0.01 | -0.10 | -0.07 | -0.08 |
| H_NU_lev2 | -0.19 | -0.08 | 0.04 | 0.13 | 0.07 | -0.04 | 0.15 | -0.05 | 0.00 | 0.01 | 0.36 | -0.10 | -0.07 | 0.07 | 0.03 | -0.12 | 0.10 | -0.10 | 0.04 | **-0.67** | 0.09 | -0.14 | 0.05 | -0.07 |
| SH_212000 | -0.33 | -0.03 | -0.16 | 0.13 | -0.11 | -0.03 | 0.11 | -0.04 | -0.07 | 0.20 | -0.03 | -0.13 | 0.01 | 0.06 | 0.00 | -0.18 | 0.09 | 0.05 | 0.15 | **0.55** | 0.11 | -0.19 | 0.06 | -0.17 |
| KH_R | -0.07 | 0.01 | 0.00 | 0.17 | 0.09 | -0.01 | 0.01 | 0.00 | -0.14 | 0.05 | -0.01 | 0.02 | -0.03 | 0.04 | -0.09 | 0.03 | -0.02 | -0.07 | -0.06 | 0.03 | **0.69** | 0.10 | 0.00 | 0.11 |
| KH_T | 0.03 | -0.03 | 0.00 | -0.12 | -0.05 | -0.04 | 0.00 | -0.01 | 0.13 | -0.04 | 0.01 | -0.05 | 0.03 | -0.07 | 0.08 | -0.05 | 0.01 | 0.10 | 0.00 | -0.03 | **0.67** | 0.07 | -0.06 | 0.03 |
| KH_S | 0.02 | -0.02 | 0.01 | 0.13 | 0.00 | -0.04 | 0.02 | 0.01 | 0.02 | -0.05 | -0.02 | 0.13 | 0.01 | 0.05 | 0.03 | 0.05 | 0.07 | -0.01 | 0.14 | -0.02 | 0.19 | **0.65** | -0.04 | -0.16 |
| SH_211000 | -0.04 | -0.05 | 0.11 | 0.21 | 0.36 | -0.21 | 0.12 | -0.02 | -0.23 | 0.00 | -0.07 | 0.12 | -0.01 | -0.03 | -0.06 | -0.05 | 0.07 | -0.18 | 0.22 | -0.10 | 0.35 | -0.39 | 0.05 | -0.30 |
| Release | 0.00 | 0.01 | -0.06 | -0.11 | 0.00 | -0.01 | 0.00 | 0.00 | 0.01 | -0.03 | -0.02 | 0.02 | -0.03 | 0.00 | 0.00 | 0.01 | 0.00 | -0.01 | -0.02 | -0.06 | 0.01 | 0.02 | **0.77** | -0.08 |
| Predators_n | 0.03 | -0.03 | 0.24 | 0.21 | 0.01 | -0.01 | 0.00 | -0.02 | -0.02 | 0.03 | 0.01 | -0.01 | 0.08 | -0.03 | -0.01 | -0.06 | 0.03 | 0.00 | 0.01 | 0.08 | -0.10 | -0.03 | **0.69** | 0.11 |
| Interspec_comp_n | 0.00 | -0.03 | 0.05 | 0.07 | 0.00 | 0.02 | 0.00 | 0.01 | 0.04 | 0.00 | -0.02 | 0.01 | 0.01 | 0.00 | -0.01 | -0.01 | 0.04 | -0.05 | 0.05 | -0.01 | 0.12 | -0.17 | 0.00 | **0.81** |
| Variance explained (%) | 14.8 | 8.0 | 6.5 | 5.8 | 4.3 | 3.8 | 3.7 | 3.2 | 3.0 | 2.6 | 2.5 | 2.3 | 2.1 | 2.0 | 1.9 | 1.8 | 1.6 | 1.6 | 1.5 | 1.5 | 1.4 | 1.4 | 1.3 | 1.3 |
| Extraction Method: Principal Component Analysis. Rotation Method: Varimax with Kaiser Normalization. | | | | | | | | | | | | | | | | | | | | | | | | |
| a. Rotation converged in 23 iterations. | | | | | | | | | | | | | | | | | | | | | | | | |

**Appendix E: Results of the single species models**

**Ungulates and mesocarnivores**

Table E1: Results of the generalized linear model with gamma distribution and natural logarithm as link to explain the effects of variables for ungulates (U) and mesocarnivores (C): Only the significant coefficients are given (italic: p<0.05; normal: p<0.01; bold: p<0.001). x: exclusion due to lack of variance; Suffixes indicate multicollinearity between the explanatory variables with the same letter.
S.s.: wild boar, R.r.: chamois, Ca.c.: roe deer, C.e.: red deer, V.v.: red fox, M.m.: European badger, Martens: stone and pine martens. For an explanation of the abbreviations of the explanatory variable, see Appendix A.

|  | ***S.s.*** | ***R.r.*** | ***Ca.c.*** | ***C.e.*** | ***V.v.*** | ***M.m.*** | **Martens** |
| --- | --- | --- | --- | --- | --- | --- | --- |
| Habitat community type | U | U | U | U | C | C | C |
| (Intercept) | **-6.816** | -4.646 | *-2.007* | **-5.713** | *1.331* | **-5.690** |  |
| **Abiotic drivers** |  |  |  |  |  |  |  |
| Climate |  |  |  |  |  |  |  |
| T_10 | *-0.072* | -0138 | *-0.090* |  | -0.049 |  | -0.090 |
| T_SP_10_s.d. |  | *^a^* |  | ^a^ | ^a^ | ^a^ | ^a^ |
| P_10 | 0.002 |  | **0.002** | **-0.004** | -0.001 |  |  |
| P_10_s.d. |  | **^b^** | **^a^** |  | **^b^** | **^b^** | **^b^** |
| P_W_10 | *-0.012* | *0.015^a^* |  | ^a^ | _a_ | ^a^ | ^a^ |
| **Landscape** |  |  |  |  |  |  |  |
| Ecoregions |  |  |  |  |  |  |  |
| ER_M_VB |  | **-0.084** |  | *0.025* | **0.016** |  | *0.027* |
| ER_M_S |  | **-0.084** |  |  |  | *0.016* |  |
| ER_M_F |  |  |  | **0.044** |  |  | ^c^ |
| ER_SA_F | *0.019* |  |  |  |  |  |  |
| ER_N |  | *-0.060* |  |  |  |  |  |
| Landscape diversity |  |  |  |  |  |  |  |
| H_PRD_comp1 |  |  |  | -0.317 |  | -0.326 | **-0.450** |
| H_AB_comp2 |  |  |  |  | *0.075* | *0.143* |  |
| H_S_comp3 |  |  |  | 0.438 |  |  | **0.226** |
| H_F_lev2 |  | **-0.058** |  |  | **-0.022** |  |  |
| H_G_lev2 |  |  |  |  |  |  |  |
| H_AL_lev2 |  |  |  |  | *0.076* |  |  |
| H_AB_lev2 |  |  |  |  |  |  |  |
| H_S_lev3 |  |  |  | **-3.911** |  |  |  |
| H_NU_lev2 |  |  |  |  |  | *-1.396* |  |
| H_NU_lev3 |  |  |  | *1.769* |  |  |  |
| SHDI |  | **2.742^c^** |  | **3.345^b^** | ^c^ |  | ^c^ |
| Landscape structures |  |  |  |  |  |  |  |
| S_10 |  | **0.064** |  |  |  | -0.025 |  |
| S_20 |  | *0.036* | -0.046 |  |  |  |  |
| S_30 |  | **^c^** |  | **^b^** | ^c^ |  | ^c^ |
| S_40 |  |  |  | *0.159* | **-0.094** |  |  |
| S_41 |  |  |  | 0.208 |  |  |  |
| S_42 |  | 0.179 | 0.133 | -0.131 |  |  |  |
| S_51 |  |  | **0.054** |  |  | **0.037** |  |
| S_52 |  |  | **0.025** |  |  |  | ^c^ |
| ED_comp1 | -0.298 | *-0.322* | *-0.233* |  | *0.116* |  |  |
| PD_comp2 |  |  | 0.353 |  | **0.175** |  |  |
| TCA_comp3 |  |  |  |  | **0.278** |  |  |
| ENN_comp4 |  |  |  |  |  |  | **0.315** |
| ENN_cl |  |  |  |  |  |  |  |
| Special habitats |  |  |  |  |  |  |  |
| SH_113000 |  |  |  | -0.878 |  |  | -0.575 |
| SH_122100 |  |  |  |  |  | *-0.765* |  |
| SH_131000 |  | **-1.893** | **-1.472** |  |  | *0.809* | **1.269** |
| SH_133000 |  | *-0.983* |  | 1.235 |  | *1.045* |  |
| SH_211000 |  | *0.135* |  | -0.142 |  | *0.112* |  |
| SH_212000 | *-0.469* |  |  | **-1.207** |  |  |  |
| SH_221000 |  | **0.055** |  |  |  | **0.027** | **0.030** |
| SH_222100 |  | 0.027 |  | **-0.019** |  |  | *-0.018* |
| SH_231000 |  |  |  |  |  |  |  |
| SH_232000 |  | -0112 | 0.058 |  |  |  |  |
| SH_310000 |  | 0.253 |  | **0.271** |  |  |  |
| SH_311000 |  |  | **-0.090** |  |  |  |  |
| SH_322000 |  |  |  | **-0.112** |  |  |  |
| SH_413000 | ^a^ | ^d^ |  | ^c^ | **-0.681** |  | **-1.084d** |
| SH_451000 |  |  |  |  | 0.062 |  | 0.111 |
| SH_452000 |  |  |  |  |  |  |  |
| **Mortality** |  |  |  |  |  |  |  |
| Hunting |  |  |  |  |  |  |  |
| Adj_hunt_area | *0.004* | **-0.015** |  |  |  | **0.011** |  |
| Hunt_manag (time effect) | *0.465* | **3.112^b^** | **3.759^a^** | **8.192** | **2.027^b^** | **1.056^b^** | **-1.007^b^** |
| Hunter_dens |  |  | **0.744** |  |  |  |  |
| Release | x | x | x | x | x | x | x |
| Protected status | x | x | x | x | x | x | x |
| Wildlife disease |  |  |  |  |  |  |  |
| KH_T | x | x | x | x |  | **-0.587** |  |
| KH_S | x | x | x | x |  |  |  |
| KH_R |  | -2.276 |  | *-1.822* | 0.776 |  |  |
| Competition and predation |  |  |  |  |  |  |  |
| Predators_n | x | x | **-0.036** | x | x | x | x |
| Interspec_comp_n | x | x |  |  | x | x | x |
| **Land use** |  |  |  |  |  |  |  |
| Land-use type |  |  |  |  |  | **-0.563** |  |
| LU_orchard_comp1 |  |  |  | -0.582 |  |  |  |
| LU_gras_i |  |  |  |  |  |  |  |
| LU_gras_e |  | ^e^ |  | ^d^ |  |  | **-0.043^e^** |
| LU_pasture |  | ^e^ |  | ^d^ | *-0.010* |  | **^e^** |
| LU_arable |  | -0.140 |  |  |  |  | **^d^** |
| LU_vine |  | **0.000** | -0.109 | **-0.175** |  | *0.087* | -0.121 |
| LU_aband | ^a^ | ^d^ | **-1.763** | **-0.000** |  |  |  |
| LU_forest |  | *0.022* |  |  |  |  |  |
| LU_settl |  |  |  | -0.006 | *-0.070* |  |  |
| Land-use intensity |  |  |  |  |  |  |  |
| LI_LSU_comp2 |  | **0.976** |  |  | **0.234** |  | *0.209* |
| LI_LSD |  |  | **-0.172** | **-0.251** | **0.068** |  |  |
| LI_sheep | *-0.003* |  |  | 0.006 |  |  |  |
| LI_goat |  |  | 0.009 | -0.011 |  |  | 0.008 |
| CUT_mean | **0.450** |  | **-0.979** |  |  | **1.180** | 0.462 |
| Hail_net |  |  |  | 0.006 |  | **0.008** |  |
| Tourism | 0.001 | -0.001 |  |  | -0.000 | *-0.000* | **-0.000** |
| d2n |  |  |  |  | *3.571* | 7.466 |  |
| Use of pesticides |  |  |  |  |  |  |  |
| Pest_total |  |  |  |  |  |  |  |
| Pest_arable |  |  |  |  |  |  |  |
| Pest_vine |  |  |  |  | **-0.000** |  | 0.000 |
|  |  |  |  |  |  |  |  |
| HabPrefid | 1 | 1 | 1 | 1 | 2 | 2 | 2 |
| SpeciesID | 1 | 2 | 3 | 4 | 8 | 9 | 10 |
| Sample size | 40 | 160 | 159 | 157 | 160 | 135 | 146 |
| #parameter | 6 | 35 | 29 | 32 | 31 | 25 | 30 |
| Pseudo-R^2^ (response) | 0.482 | 0.599 | 0.630 | 0.609 | 0.880 | 0.613 | 0.553 |
| Pseudo-R^2^ (link) | 0.594 | 0.888 | 0.834 | 0.925 | 0.908 | 0.693 | 0.686 |
| pvalue1 | < 0.001 | < 0.001 | < 0.001 | < 0.001 | < 0.001 | < 0.001 | < 0.001 |
| pvalue2 | 0.467 | 0.032 | 0.543 | 0.145 | 0.179 | 0.509 | 0.398 |
| maxvif | 8.913 | 7.123 | 8.233 | 5.822 | 7.871 | 6.457 | 6.288 |
| meanvif | 3.550 | 3.984 | 3.291 | 2.954 | 2.059 | 2.716 | 2.924 |

Note: #parameter denotes the number of model parameters; pvalue1 is the p-value of the test concerning the significance of the model as a whole, i.e. null model (just intercept) versus proposed model; pvalue2 is the p-value concerning model fit, i.e. proposed model versus saturated model; maxvif is the maximum of the VIF values of the independent variables in the model; meanvif is the mean of the VIF values of the independent variables in the model.

**Small alpine games and small forest games**

Table E2: Results of the generalized linear model with gamma distribution and natural logarithm as link to explain the effects of variables for small alpine games (A) and small forest games (F): Only the significant coefficients are given (italic: p<0.05; normal: p<0.01; bold: p<0.001). x: exclusion due to lack of variance; Suffixes indicate multicollinearity between the explanatory variables with the same letter.
Ma.m.: Alpine marmot, Le.t.: mountain hare, L.m.: rock ptarmigan, T.u.: capercaillie, L.t.: black grouse, T.b.: hazel grouse, S.r.: Eurasian woodcock, G.g.: Eurasian jay. For an explanation of the abbreviations of the explanatory variable, see Appendix A.

|  | ***Ma.m*** | ***Le.t.*** | ***L.m.*** | ***T.u.*** | ***L.t.*** | ***T.b.*** | ***S.r.*** | ***G.g.*** |
| --- | --- | --- | --- | --- | --- | --- | --- | --- |
| Habitat community type | A | A | A | F | F | F | F | F |
| (Intercept) | **-6.531** |  |  |  |  |  | **8.263** | **6.225** |
| **Abiotic drivers** |  |  |  |  |  |  |  |  |
| Climate |  |  |  |  |  |  |  |  |
| T_10 | -0.215 | **-0.197** | -0.195 |  |  |  | *-0.125* |  |
| T_SP_10_s.d. |  |  |  |  |  |  |  |  |
| P_10 |  | ^a^ | *^a^* | *0.001* | *0.002* | ^a^ |  |  |
| P_10_s.d. |  | **b** | **^b^** | ^a^ | **^a^** | *0.009^b^* |  | **a** |
| P_W_10 |  | ^a^ | *-0.013^a^* |  | ^b^ | ^a^ | **-0.033** | ^b^ |
| **Landscape** |  |  |  |  |  |  |  |  |
| Ecoregions |  |  |  |  |  |  |  |  |
| ER_M_VB | *-0058* |  |  |  | -0.028 |  |  |  |
| ER_M_S | **-0.88** | 0.040 |  | **-0.037** |  | **0.057** | 0.031 |  |
| ER_M_F | **^a, b^** | **^c^** | *-0.030^c^* | **-0.045^b^** | ^b^ | ^c^ |  | ^b^ |
| ER_SA_F |  | **-0.076** | **-0.073** |  |  | **-0.041** | **-0.076** | **-0.071** |
| ER_N |  |  | *0.090* |  |  |  |  |  |
| Landscape diversity |  |  |  |  |  |  |  |  |
| H_PRD_comp1 |  |  | *-0.334* |  |  | *0.370^h^* |  |  |
| H_AB_comp2 |  |  |  | *-0.189* |  | *-0.410* | *0.197* |  |
| H_S_comp3 |  | *-0.217* |  |  |  |  | **-0.425** |  |
| H_F_lev2 | *0.043* | **0.041** |  |  |  |  |  | -0.047 |
| H_G_lev2 |  |  |  |  | **-0.033** |  |  |  |
| H_AL_lev2 |  |  |  |  |  |  |  | 0.181 |
| H_AB_lev2 |  |  |  |  | *-0.085* |  |  |  |
| H_S_lev3 | **4.790** |  |  |  |  |  |  |  |
| H_NU_lev2 |  | *-0.674* | -0.940 | *-0.506* |  |  |  |  |
| H_NU_lev3 |  | *-2.397* |  |  |  |  | *-1.586* |  |
| SHDI | **^a, b^** | **^c^** | ^c^ | **^b, c^** | ^b^ | ^c^ | ^a^ | *-1.581* |
| Landscape structures |  |  |  |  |  |  |  |  |
| S_10 | **-0.067** |  | **-0.044** |  |  |  |  |  |
| S_20 | *0.070* | **-0.063** | **-0.081** | **0.045** | -0.024 |  |  | *-0.021* |
| S_30 | ^a^ | **^c^** | *^c^* | **^b, c^** | ^b^ | ^c^ |  | ^b^ |
| S_40 |  | **0.233** |  |  | *-0.106* |  |  |  |
| S_41 |  |  |  |  | -0.128 |  |  |  |
| S_42 |  |  | **-0.236** | **-0.302** | **-0.194** | -0.143 |  |  |
| S_51 |  |  |  |  |  |  | **-0.052** |  |
| S_52 | **^b^** | **0.043^c^** | *^c^* | **^b, c^** | ^b, c^ | ^c, d^ | ^a^ |  |
| ED_comp1 |  | **0.897** | **0.881** | 0.305 |  |  |  | 0.398 |
| PD_comp2 | *-0.576* |  |  | **0.798** |  | 0.535 | **0.378** |  |
| TCA_comp3 | **1.228** |  | **0.828** | 0.352^h^ | **0.626** |  | ^b^ | **0.498^c^** |
| ENN_comp4 | *-0.003* |  |  | **-0.330** |  |  |  |  |
| ENN_cl |  |  | *0.002* | -0.002 | **-0.002** | *0.002* | -0.003 | **-0.002** |
| Special habitats |  |  |  |  |  |  |  |  |
| SH_113000 |  |  | **1.875** | *0.920* |  | *0.603* | 1.265 |  |
| SH_122100 |  | *-0.991* |  |  |  | 0.992 |  |  |
| SH_131000 |  |  |  |  | 1.324 |  | -1.929 | **1.712** |
| SH_133000 | 2.225 | *1.083* |  |  |  |  |  | *1.699* |
| SH_211000 |  | *-0.089* |  |  | *-0.084* | **-0.193** | -0.419 |  |
| SH_212000 |  | **-1.187** |  |  |  |  |  | *-0.728* |
| SH_221000 | **0.051** | -0.040 |  |  |  |  |  |  |
| SH_222100 | ^e^ | **-0.060** | **-0.045** | ^d^ | **-0.036** | ^e^ | **-0.034** |  |
| SH_231000 |  |  |  |  |  | -0.436 |  |  |
| SH_232000 |  | **-0.181** | **-0.169** | **-0.136** |  |  |  | **-0.138** |
| SH_310000 |  | *-0140* | -0.340 |  | -0.154 |  |  | *-0.193* |
| SH_311000 |  |  |  | **-0.090** | -0.058 |  | **-0.380** |  |
| SH_322000 | 0.077 |  | **0.167** |  | **0.077** | *0.043* |  |  |
| SH_413000 | ^d^ | -0.873 | **^d^** | *-0.470^f^* | *-0.480^d^* | *-0.763^g^* | ^d^ | **-1.127^d^** |
| SH_451000 |  |  |  | 0.201 |  |  | *0.127* |  |
| SH_452000 |  | *-0.619* | *-0.812* |  |  | **-0.993** |  | *-0.505* |
| **Mortality** |  |  |  |  |  |  |  |  |
| Hunting |  |  |  |  |  |  |  |  |
| Adj_hunt_area |  | **0.015** |  |  |  | **0.012** | ^b^ | **^c^** |
| Hunt_manag (time effect) | **6.398** | **2.039^b^** | **2.436^b^** | ^a^ | **-1.397^a^** | **1.232^b^** | **1.635** | **2.036^a^** |
| Hunter_dens | **-2.905** | *-0.532* | **-1.204** |  |  | *h* |  |  |
| Release | x | x | x | x | x | x | x | x |
| Protected status | x | x | x |  | x |  | x | x |
| Wildlife disease |  |  |  |  |  |  |  |  |
| KH_T | x | x | x | x | x | x | x | x |
| KH_S | x | x | x | x | x | x | x | x |
| KH_R | x | x | x | x | x | x | x | x |
| Competition and predation |  |  |  |  |  |  |  |  |
| Predators_n |  |  |  |  | *-0.006* |  | x | x |
| Interspec_comp_n |  | x | x |  | x | x | x | x |
| **Land use** |  |  |  |  |  |  |  |  |
| Land-use type |  |  |  |  |  |  |  |  |
| LU_orchard_comp1 |  |  |  | **-2.820** |  | -0.576 |  | **0.840** |
| LU_gras_i | *0.070* |  | **0.094** |  | 0.031 | *-0.035* |  |  |
| LU_gras_e | ^c^ | ^d^ | **0.059^e^** | ^e^ |  | ^f^ | **^c^** |  |
| LU_pasture | ^c^ | ^d^ | **e** | ^e^ |  | ^f^ | **-0.073^c^** |  |
| LU_arable |  | *0.063* |  |  |  |  | -0.107 |  |
| LU_vine |  |  |  | ^g^ |  |  | -0.112 | ^d^ |
| LU_aband | ^d^ |  | **-1.500^d^** | **-0.270^f^** | -0.096^d^ | -0.124^g^ |  | **^d^** |
| LU_forest | **-0.061^a^** |  |  | ^c^ | 0.016^c^ | ^d^ | ^d^ | *0.017* |
| LU_settl |  |  | **0.460** |  |  |  | *-0.186* |  |
| Land-use intensity |  |  |  |  |  |  |  |  |
| LI_LSU_comp2 | **-1.429** |  | **-1.003** | ^h^ | **-0.433** |  |  |  |
| LI_LSD |  |  |  |  |  |  |  | *0.131* |
| LI_sheep | **0.014** |  | **0.012** | 0.005 | 0.004 |  | 0.006 |  |
| LI_goat |  |  |  | 0.009 |  |  |  |  |
| CUT_mean | **2.375** | **-0.005** | **1.295** |  | **0.487** |  |  | **1.705** |
| Hail_net |  |  |  |  |  |  |  |  |
| Tourism | **0.000** |  | **0.000** |  | **0.000** |  | *0.000* |  |
| d2n | ^e^ |  |  | ^d^ |  | ^e^ |  | -8.343 |
| Use of pesticides |  |  |  |  |  |  |  |  |
| Pest_total |  |  |  |  |  |  |  |  |
| Pest_arable |  |  |  |  |  |  |  |  |
| Pest_vine |  |  |  | ^g^ |  | *0.000* |  | ^d^ |
|  |  |  |  |  |  |  |  |  |
| HabPrefid | 3 | 3 | 3 | 5 | 5 | 5 | 5 | 5 |
| SpeciesID | 5 | 7 | 14 | 11 | 12 | 13 | 20 | 25 |
| sample_size | 160 | 155 | 160 | 80 | 145 | 94 | 106 | 116 |
| #parameter | 27 | 36 | 34 | 26 | 34 | 35 | 31 | 31 |
| Pseudo-R^2^ (response) | 0.249 | 0.350 | 0.533 | 0.854 | 0.656 | 0.825 | 0.536 | 0.738 |
| Pseudo-R^2^ (link) | 0.845 | 0.821 | 0.865 | 0.892 | 0.825 | 0.843 | 0.790 | 0.836 |
| pvalue1 | < 0.001 | < 0.001 | < 0.001 | < 0.001 | < 0.001 | < 0.001 | < 0.001 | < 0.001 |
| pvalue2 | 0.484 | 0.053 | 0.221 | 0.220 | 0.310 | 0.170 | 0.313 | 0.119 |
| maxvif | 8.438 | 9.736 | 7.846 | 7.362 | 10.571 | 7.899 | 7.899 | 8.547 |
| meanvif | 3.258 | 3.646 | 3.742 | 3.444 | 2.345 | 3.710 | 3.710 | 4.244 |

Note: #parameter denotes the number of model parameters; pvalue1 is the p-value of the test concerning the significance of the model as a whole, i.e. null model (just intercept) versus proposed model; pvalue2 is the p-value concerning model fit, i.e. proposed model versus saturated model; maxvif is the maximum of the VIF values of the independent variables in the model; meanvif is the mean of the VIF values of the independent variables in the model.

**Small farmland games**

Table E3: Results of the generalized linear model with gamma distribution and natural logarithm as link to explain the effects of variables for small farmland games (FL): Only the significant coefficients are given (italic: p<0.05; normal: p<0.01; bold: p<0.001). x: exclusion due to lack of variance; Suffixes indicate multicollinearity between the explanatory variables with the same letter.
Co.c.: common quail, Pe.p.: grey partridge, Ph.c.: common pheasant, C.p.: common wood pigeon, A.g.: rock partridge, L.e.: Eurasian hare. For an explanation of the abbreviations of the explanatory variable, see Appendix A.

|  | ***Pe.p.*** | ***Ph.c.^1^*** | ***C.p.*** | ***Co.c.*** | ***A.g.*** | ***L.e.*** |
| --- | --- | --- | --- | --- | --- | --- |
| Habitat community type | FL | FL | FL | FL | FL | FL |
| (Intercept) |  | **11.755** |  |  | **9.964** |  |
| **Abiotic drivers** |  |  |  |  |  |  |
| Climate |  |  |  |  |  |  |
| T_10 |  |  |  |  | **-0.600** |  |
| T_SP_10_s.d. | ^a^ | ^a^ | ^a^ |  | ^a^ |  |
| P_10 |  | *0.003* | -0.005 |  | **-0.006** |  |
| P_10_s.d. | **^b^** | ^b^ | ^b^ |  | **^b^** | **^a^** |
| P_W_10 | ^a^ | ^a^ | ^a^ |  | ^a^ |  |
| **Landscape** |  |  |  |  |  |  |
| Ecoregions |  |  |  |  |  |  |
| ER_M_VB | **0.095** | **-0.155** |  |  |  |  |
| ER_M_S |  |  | **0.037** |  | **0.083** | **0.042** |
| ER_M_F | ^c^ | -0.060^c^ | **^c^** | **-0.029^c^** | **-0.106^c^** | **0.027^b^** |
| ER_SA_F |  |  | -0.044 | **-0.053** | **-0.194** |  |
| ER_N | *0.182* |  | 0.141 |  |  | *0.037* |
| Landscape diversity |  |  |  |  |  |  |
| H_PRD_comp1 |  |  | ^e^ |  | *0.493e* | 0.226 |
| H_AB_comp2 | *-0.342* | *0.342* |  |  |  |  |
| H_S_comp3 | **1.738** |  |  | *0.127* |  | -0.253 |
| H_F_lev2 |  |  |  |  | -0,070 |  |
| H_G_lev2 |  |  | 0.057 |  | -0.051 | *0.013* |
| H_AL_lev2 |  | -0.550 | *0.296* |  | *0.214* | *0.074* |
| H_AB_lev2 |  |  |  |  |  |  |
| H_S_lev3 |  |  |  |  |  | *1.071* |
| H_NU_lev2 |  |  |  |  |  |  |
| H_NU_lev3 |  |  |  |  |  |  |
| SHDI | ^c, d^ | ^c^ | **4.297^c, g^** |  | **^c, g^** | **1.735^b^** |
| Landscape structures |  |  |  |  |  |  |
| S_10 |  |  |  |  |  | *0.014* |
| S_20 | *-0.095* | *0.079* | **-0.065** |  |  |  |
| S_30 | ^c, d, e^ | ^c^ | -0.083^c, g^ |  | **^c, g^** | **^b^** |
| S_40 |  |  |  |  |  |  |
| S_41 | *-0.379* |  |  |  | -0.492 |  |
| S_42 |  | *-0.212* |  |  |  | **0.128** |
| S_51 |  |  |  |  |  |  |
| S_52 | ^e^ |  | ^g^ | *-0.013* | ^g^ | **^b^** |
| ED_comp1 | 0.668 | **-1.612** |  |  |  |  |
| PD_comp2 | *-0.767* |  |  |  |  |  |
| TCA_comp3 |  |  | **0.986^d, i^** |  | **0.956^d. i^** | **0.419** |
| ENN_comp4 |  | -0.565 |  |  |  |  |
| ENN_cl |  |  |  | 0.001 | **0.006** |  |
| Special habitats |  |  |  |  |  |  |
| SH_113000 |  |  |  |  | **2.195** |  |
| SH_122100 |  |  |  |  |  |  |
| SH_131000 |  |  | *1.419* |  |  |  |
| SH_133000 | **4.346** |  |  |  |  |  |
| SH_211000 |  | *-0.178* | 0.243 |  | **-0.343** | -0.095 |
| SH_212000 | *2.765* |  |  |  |  |  |
| SH_221000 | -0.053 |  |  | **0.043** |  | ^d^ |
| SH_222100 | **-0.068** | *-0.038* | **0.041** |  |  |  |
| SH_231000 | *-0.597* |  |  |  | **-0.711** |  |
| SH_232000 | -0.165 | **0.371** |  |  |  | *0.044* |
| SH_310000 | -0.515 |  | -0.296 |  | **-0.859** | -0.124 |
| SH_311000 |  |  |  |  |  |  |
| SH_322000 |  | -0.097 |  |  |  |  |
| SH_413000 | ^g^ | ^d^ |  |  |  | **-0.577** |
| SH_451000 |  |  |  |  |  |  |
| SH_452000 |  | 1.163 |  |  | 1.354 |  |
| **Mortality** |  |  |  |  |  |  |
| Hunting |  |  |  |  |  |  |
| Adj_hunt_area |  |  | ^d^ |  | **^d^** |  |
| Hunt_manag (time effect) | **-5.905^b^** | 1.324^b^ | -1.325^b^ | **-5.578** | **-3.451^b^** | **-0.870^a^** |
| Hunter_dens | *2.129* |  | 0.800^e^ | *-0.328* | *^e^* | **-0.783** |
| Release | x |  | x | x | x | x |
| Protected status |  | x | x | x | x | x |
| Wildlife disease |  |  |  |  |  |  |
| KH_T | x | x | x | x | x | x |
| KH_S | x | x | x | x | x | x |
| KH_R | x | x | x | x | x | x |
| Competition and predation |  |  |  |  |  |  |
| Predators_n |  | **-0.032** |  |  |  | -0.009 |
| Interspec_comp_n | x | x | x | x | x | x |
| **Land use** |  |  |  |  |  |  |
| Land-use type |  |  |  |  |  |  |
| LU_orchard_comp1 | ^f^ |  | ^f^ | **-0.371** | ^f^ |  |
| LU_gras_i |  | **0.184** |  |  |  | -0.025 |
| LU_gras_e | ^h^ |  | ^h^ |  | ^h^ |  |
| LU_pasture | ^h^ | **-0.101** | ^h^ |  | ^h^ |  |
| LU_arable |  | **0.507** |  |  |  |  |
| LU_vine |  |  |  |  | 0.176 | 0.074 |
| LU_aband | ^g^ | 3.483^d^ |  |  |  |  |
| LU_forest | ^e^ |  | **0.050** |  | ^g^ |  |
| LU_settl | *-0.880* |  |  |  |  | 0.426 |
| Land-use intensity |  |  |  |  |  |  |
| LI_LSU_comp2 |  |  | ^i^ |  | **^i^** |  |
| LI_LSD |  |  |  |  |  | *-0.080* |
| LI_sheep |  | *0.009* |  |  |  |  |
| LI_goat |  |  |  | **0.009** |  |  |
| CUT_mean | **2.717** | **1.562** |  | *0.434* | **1.868** | 0.426 |
| Hail_net | x |  |  | **0.005** |  | *0.002* |
| Tourism | *0.000* |  |  |  | 0.000 |  |
| d2n |  | 18.313 |  | **10.956** |  | ^d^ |
| Use of pesticides |  |  |  |  |  |  |
| Pest_total | ^f^ |  | ^f^ |  | ^f^ |  |
| Pest_arable |  |  |  |  |  |  |
| Pest_vine |  | *0.000* | **0.000** | **0.000** |  | **0.000** |
|  |  |  |  |  |  |  |
| HabPrefid | 6 | 6 | 6 | 6 | 6 | 6 |
| SpeciesID | 16 | 18 | 19 | 17 | 15 | 6 |
| Sample size | 80 | 120 | 92 | 160 | 87 | 160 |
| #parameter | 30 | 33 | 26 | 31 | 34 | 40 |
| Pseudo-R^2^ (response) | 0.607 | 0.761 | 0.799 | 0.842 | 0.616 | 0.826 |
| Pseudo R^2^ (link) | 0.919 | 0.866 | 0.815 | 0.963 | 0.898 | 0.898 |
| pvalue1 | < 0.001 | < 0.001 | < 0.001 | < 0.001 | < 0.001 | < 0.001 |
| pvalue2 | 0.473 | 0.480 | 0.477 | 0.480 | 0.474 | 0.557 |
| maxvif | 6.222 | 9.710 | 6.199 | 7.802 | 9.310 | 7.668 |
| meanvif | 3.667 | 4.217 | 2.163 | 3.608 | 2.324 | 4.434 |

^1^ Due to the data, the model result should be interpreted with caution. For the analyses based on the results of the single-species models, the results were not considered.

Note: #parameter denotes the number of model parameters; pvalue1 is the p-value of the test concerning the significance of the model as a whole, i.e. null model (just intercept) versus proposed model; pvalue2 is the p-value concerning model fit, i.e. proposed model versus saturated model; maxvif is the maximum of the VIF values of the independent variables in the model; meanvif is the mean of the VIF values of the independent variables in the model.

**Synanthropic games and waterflows**

Table E4: Results of the generalized linear model with gamma distribution and natural logarithm as link to explain the effects of variables for synanthropic games (S) and waterflows (W): Only the significant coefficients are given (italic: p<0.05; normal: p<0.01; bold: p<0.001). x: exclusion due to lack of variance; Suffixes indicate multicollinearity between the explanatory variables with the same letter.
Cor.c.: carrion crow, Pi.p.: Eurasian magpie, T.m.: Common blackbird, T.ph.: Song thrush, T.pi.: Fieldfare, ducks: mallard, garganey, common teal, F.a.: Eurasian coot. For an explanation of the abbreviations of the explanatory variable, see Appendix A.

|  | ***Cor.c.*** | ***Pi.p.*** | ***T.m.*** | ***T.ph.^1^*** | ***T.pi.*** | ***ducks*** | ***F.a.*** |
| --- | --- | --- | --- | --- | --- | --- | --- |
| Habitat community type | S | S | S | S | S | W | W |
| (Intercept) | **6.258** | 1.672 | **-4.426** |  | **-12.189** | **-15.902** |  |
| **Abiotic drivers** |  |  |  |  |  |  |  |
| Climate |  |  |  |  |  |  |  |
| T_10 |  |  |  |  |  |  | -0.168 |
| T_SP_10_s.d. |  |  |  |  |  |  |  |
| P_10 | ^a^ |  |  | **-0.010** |  |  |  |
| P_10_s.d. | **^b^** | ^a^ |  | 0.162 |  |  | **^a^** |
| P_W_10 | ^a^ |  |  |  |  | -0.019 | -0.014 |
| **Landscape** |  |  |  |  |  |  |  |
| Ecoregions |  |  |  |  |  |  |  |
| ER_M_VB |  |  |  |  |  |  | 0.058 |
| ER_M_S |  |  |  | -0.121 | **0.119** | **0.103** | **0.084** |
| ER_M_F | **-0.094^c, d^** | ^b^ | ^c, d^ |  |  | **0.112^a, b^** | ^b^ |
| ER_SA_F | **-0.101** |  |  | -0.092 |  |  |  |
| ER_N |  |  | **0.298** |  | 0.306 | **0.101** |  |
| Landscape diversity |  |  |  |  |  | 0.143 |  |
| H_PRD_comp1 |  | ^f^ | -0.875 |  |  |  |  |
| H_AB_comp2 | -0.352 | **-0.472** | -0.792 |  | 0.568 |  |  |
| H_S_comp3 |  |  | **1.057** |  |  |  |  |
| H_F_lev2 | -0.051 |  | -0.108 |  |  | -0.046 |  |
| H_G_lev2 |  |  | **-0.124** |  |  |  |  |
| H_AL_lev2 | **0.470** |  |  | 1.103 |  |  | 0.234 |
| H_AB_lev2 |  |  | **0.743** |  |  |  |  |
| H_S_lev3 |  | 1.628 |  |  |  |  |  |
| H_NU_lev2 |  |  | **-3.316** |  |  | -1.318 |  |
| H_NU_lev3 |  |  |  |  |  |  |  |
| SHDI | **^c, d^** | ^b, c^ | ^c, d^ |  |  | **5.688^a, b^** | ^b^ |
| Landscape structures |  |  |  |  |  |  |  |
| S_10 |  |  |  |  |  |  |  |
| S_20 |  |  |  | -0.182 |  |  |  |
| S_30 | ^d^ | ^b^ | ^c^ |  |  | **^a^** | ^b^ |
| S_40 |  | 0.200 |  |  |  |  |  |
| S_41 |  |  |  |  |  |  |  |
| S_42 |  | **0.484** | **0.628** |  |  | -0.247 | -0.206 |
| S_51 |  |  |  |  | -0.154 |  |  |
| S_52 | **^c^** | ^c^ | ^d^ |  |  | **^b^** |  |
| ED_comp1 |  | **0.647** | 0.516 | **2.115** |  | **-0.941** | **-0.838** |
| PD_comp2 |  |  | 0.617 |  |  | 0.621 |  |
| TCA_comp3 | 0.417^e^ | 0.364^d^ | ^g, h^ |  |  | ^c^ |  |
| ENN_comp4 |  |  |  |  |  |  |  |
| ENN_cl |  | 0.002 |  | 0.005 |  | -0.003 |  |
| Special habitats |  |  |  |  |  |  |  |
| SH_113000 |  |  |  |  | -2.372 |  |  |
| SH_122100 |  | -1.857 | -5.271 |  |  | **2.016** |  |
| SH_131000 |  |  |  |  |  |  | -1.811 |
| SH_133000 |  | **2.294^g^** | ^e^ |  |  | x | -1.443 |
| SH_211000 |  | **-0.213** |  |  |  |  | -0.141 |
| SH_212000 |  |  | -1.571 |  |  | -1.877 | -1.015 |
| SH_221000 | 0.028 |  |  |  |  | **-0.088** |  |
| SH_222100 | ^f^ |  |  |  |  |  | -0.028 |
| SH_231000 |  | **0.484** | **1.084** |  |  |  |  |
| SH_232000 | **-0.186** | 0.081 |  |  |  |  | 0.118 |
| SH_310000 |  | **-0.323** | **-0.797** |  | -0.603 |  |  |
| SH_311000 |  | 0.103 |  |  |  |  |  |
| SH_322000 |  | **0.129** |  | 0.154 |  |  |  |
| SH_413000 | -0.962 | -0.744^e^ | **-15.710** |  | -5.912 |  |  |
| SH_451000 |  | -0.174 |  |  |  |  |  |
| SH_452000 |  |  |  |  | -4.039 |  | -0.992 |
| **Mortality** |  |  |  |  |  |  |  |
| Hunting |  |  |  |  |  |  |  |
| Adj_hunt_area | ^e^ |  | ^h^ |  |  | ^c^ | 0.008 |
| Hunt_manag (time effect) | **-2.916^b^** | -1.006^a^ |  |  |  | **2.661** | **-4.249^a^** |
| Hunter_dens | 0.928 | ^f^ | **3.175** |  |  | 1.125 |  |
| Release | x | x | x | x | x | x | x |
| Protected status | x | x | x | x | x | x | x |
| Wildlife disease |  |  |  |  |  |  |  |
| KH_T | x | x | x | x | x | x | x |
| KH_S | x | x | x | x | x | x | x |
| KH_R | x | x | x | x | x | x | x |
| Competition and predation |  |  |  |  |  |  |  |
| Predators_n | x | x | x | x | x | x | x |
| Interspec_comp_n | 0.032 | **0.029** | x | x | x | x | x |
| **Land use** |  |  |  |  |  |  |  |
| Land-use type |  |  |  |  |  |  |  |
| LU_orchard_comp1 | -0.500 | 0.570 |  |  |  |  | **-0.664** |
| LU_gras_i |  |  | **-0.318** |  |  | **0.116** |  |
| LU_gras_e | -0.057 |  |  |  |  | 0.054 | ^c^ |
| LU_pasture |  | **-0.060** | **^f^** |  |  |  | ^c^ |
| LU_arable |  | 0.151 | **-0.671^f^** |  |  |  |  |
| LU_vine |  | **^g^** | ^e^ |  |  |  |  |
| LU_aband |  | ^e^ | **4.647** |  |  |  | -2.261 |
| LU_forest |  | -0.021 | **0.075** |  |  |  |  |
| LU_settl | **0.446** | -0.234 |  |  |  |  |  |
| Land-use intensity |  |  |  |  |  |  | 0.237 |
| LI_LSU_comp2 | 0.634 |  | ^h^ |  |  |  |  |
| LI_LSD | **0.225** |  |  |  |  |  | 0.185 |
| LI_sheep | -0.010 | 0.005 | **0.035** |  | **0.044** | 0.009 |  |
| LI_goat |  |  |  |  | **-0.047** |  |  |
| CUT_mean |  |  |  |  | **6.142** | **3.463** |  |
| Hail_net | 0.004 |  |  |  |  |  |  |
| Tourism | -0.000 |  |  |  |  | **0.000** |  |
| d2n | ^f^ |  | ^g^ |  |  | -22.722 |  |
| Use of pesticides |  |  |  |  |  |  |  |
| Pest_total |  |  |  |  |  |  |  |
| Pest_arable | **-0.002** |  |  |  |  |  | **-0.002** |
| Pest_vine | **-0.000** | **^g^** | ^e^ |  |  |  | **0.000** |
|  |  |  |  |  |  |  |  |
| HabPrefid | 7 | 7 | 7 | 7 | 7 | 8 | 8 |
| SpeciesID | 23 | 24 | 26 | 27 | 28 | 21 | 22 |
| Sample size | 118 | 95 | 80 | 39 | 71 | 155 | 120 |
| #parameters | 33 | 37 | 25 | 20 | 22 | 32 | 29 |
| Pseudo-R^2^ (response) | 0.767 | 0.851 | 0.874 | 0.799 | 0.334 | 0.473 | 0.905 |
| Pseudo-R^2^ (link) | 0.844 | 0.894 | 0.939 | 0.870 | 0.782 | 0.747 | 0.887 |
| pvalue1 | < 0.001 | < 0.001 | < 0.001 | < 0.001 | < 0.001 | < 0.001 | < 0.001 |
| pvalue2 | 0.479 | 0.534 | 0.474 | 0.456 | 0.473 | 0.483 | 0.479 |
| maxvif | 8.711 | 8.442 | 9.460 | 5.061 | 6.108 | 8.648 | 7.937 |
| meanvif | 4.322 | 3.725 | 3.726 | 2.622 | 2.343 | 3.468 | 3.512 |

^1^ Due to the data, the model result should be interpreted with caution. For the analyses based on the results of the single-species models, the results were not considered.

Note: #parameter denotes the number of model parameters; pvalue1 is the p-value of the test concerning the significance of the model as a whole, i.e. null model (just intercept) versus proposed model; pvalue2 is the p-value concerning model fit, i.e. proposed model versus saturated model; maxvif is the maximum of the VIF values of the independent variables in the model; meanvif is the mean of the VIF values of the independent variables in the model.

**Appendix F: Results concerning overfitting of models**

Table F1: Checking for overfitting by splitting the sample into a training set (80%) and a validation set (20%) and calculating the root mean square error (RMSE) between fitted/predicted and observed values for 1000 random splits.
Abbreviations: n = sample_size; df = Model degrees of freedom; RMSE_T_ = mean RMSE on the training set; SD RMSE_T_ = standard deviation of RMSE on the training set; RMSE_V_ = mean RMSE on the validation set, p value for a significant difference between training and validation RMSE.

| Species | Habitat guild | Sample size (n) | df | RMSE (all data) | Mean RMSE_T_ | SD RMSE_T_ | Mean RMSE_V_ | p value |
| --- | --- | --- | --- | --- | --- | --- | --- | --- |
| Wild boar | Ungulates | 40 | 33 | 3.981 | 3.994 | 0.079 | 3.949 | 0.571 |
| Chamois | Ungulates | 160 | 124 | 29.751 | 30.075 | 5.589 | 30.546 | 0.933 |
| Roe deer | Ungulates | 159 | 129 | 62.157 | 59.103 | 10.621 | 69.910 | 0.309 |
| Red deer | Ungulates | 157 | 124 | 25.977 | 23.007 | 10.472 | 26.272 | 0.755 |
| Red fox | Mesocarnivores | 160 | 128 | 6.490 | 6.679 | 0.366 | 7.142 | 0.206 |
| European badger | Mesocarnivores | 135 | 109 | 0.753 | 0.750 | 0.038 | 0.795 | 0.238 |
| Martens | Mesocarnivores | 146 | 115 | 1.482 | 1.474 | 0.071 | 1.534 | 0.398 |
| Alpine marmot | Small alpine games | 160 | 132 | 24.236 | 23.349 | 5.083 | 22.334 | 0.842 |
| Mountain hare | Small alpine games | 155 | 118 | 3.679 | 3.555 | 0.560 | 4.048 | 0.379 |
| Rock ptarmigan | Small alpine games | 160 | 125 | 14.225 | 14.285 | 4.290 | 18.029 | 0.383 |
| Capercaillie | Small forest games | 80 | 53 | 0.925 | 0.896 | 0.109 | 1.039 | 0.190 |
| Black grouse | Small forest games | 145 | 110 | 2.644 | 2.473 | 0.581 | 2.759 | 0.623 |
| Hazel grouse | Small forest games | 94 | 58 | 3.249 | 3.218 | 0.419 | 3.888 | 0.109 |
| Eurasian woodcock | Small forest games | 106 | 74 | 2.867 | 2.898 | 0.412 | 3.449 | 0.181 |
| Eurasian jay | Small forest games | 116 | 84 | 24.091 | 23.963 | 3.300 | 26.924 | 0.370 |
| Eurasian hare | Small farmland games | 160 | 119 | 18.955 | 18.410 | 1.498 | 20.757 | 0.117 |
| Rock partridge | Small farmland games | 87 | 52 | 3.291 | 3.147 | 0.688 | 3.865 | 0.297 |
| Grey partridge | Small farmland games | 80 | 49 | 2.712 | 2.675 | 0.430 | 3.230 | 0.197 |
| Common quail | Small farmland games | 120 | 88 | 1.581 | 1.646 | 0.209 | 1.885 | 0.253 |
| Common wood pigeon | Small farmland games | 92 | 65 | 5.026 | 5.136 | 0.540 | 6.021 | 0.101 |
| Carrion crow | Synanthropic games | 118 | 84 | 19.242 | 10.386 | 1.890 | 13.124 | 0.148 |
| Eurasian magpie | Synanthropic games | 95 | 57 | 6.024 | 6.410 | 1.033 | 7.618 | 0.242 |
| Common blackbird | Synanthropic games | 80 | 54 | 42.090 | 52.851 | 18.238 | 74.907 | 0.227 |
| fFeldfare | Synanthropic games | 71 | 48 | 40.345 | 40.909 | 13.013 | 51.090 | 0.434 |
| Ducks | Waterfowls | 155 | 122 | 8.359 | 8.907 | 2.034 | 10.477 | 0.440 |
| Eurasian coot | Waterfowls | 120 | 90 | 1.277 | 1.566 | 0.568 | 2.178 | 0.281 |

**Literature**

1. Troll, C. High Mountain Belts between the Polar Caps and the Equator: Their Definition and Lower Limit. *Arct. Alp. Res.* **5**, A19–A27 (1973).

2. Chimani, B., Matulla, C., Böhm, R. & Hofstätter, M. A new high resolution absolute temperature grid for the Greater Alpine Region back to 1780. *Int. J. Climatol.* **33**, 2129–2141 (2013).

3. Hiebl, J. *et al.* A high-resolution 19611990 monthly temperature climatology for the greater Alpine region. *Meteorol. Z.* **18**, 507–530 (2009).

4. Efthymiadis, D. *et al.* Construction of a 10-min-gridded precipitation data set for the Greater Alpine Region for 1800–2003. *J. Geophys. Res.* **111**, D01105 (2006).

5. Tasser, E., Ruffini, F. V. & Tappeiner, U. An integrative approach for analysing landscape dynamics in diverse cultivated and natural mountain areas. *Landsc. Ecol.* **24**, 611–628 (2009).

6. McGarigal, K. & Marks, B. J. *FRAGSTATS: spatial pattern analysis program for quantifying landscape structure.* PNW-GTR-351 https://www.fs.usda.gov/treesearch/pubs/3064 (1995) doi:10.2737/PNW-GTR-351.

7. Kapfer, A. Beitrag zur Geschichte des Grünlands Mitteleuropas Darstellung im Kontext der landwirtschaftlichen Bodennutzungssysteme im Hinblick auf den Artenund Biotopschutz. *Naturschutz Landschaftsplanung* **42**, 133–140 (2010).

8. *Wir Landschaftmacher: vom Sein und Werden der Kulturlandschaft in Nord-, Ost- und Südtirol*. (Athesia, 2012).

9. Amt für Umwelt. Umweltglossar. Düngergrossvieheinheit (DGVE). Preprint at https://www.umwelt.sg.ch/g/glossar_umwelt.Char__D.html (2017).

10. Bio Suisse. Bio Suisse Weisung „Nährstoffversorgung“. Entwurf vom 1.7.2008. Preprint at https://www.bio-suisse.ch/media/de/pdf2008/Anbau/nhrstoffversorgung.pdf (2008).

11. Schweizerische Bundeskanzlei. Bundesgesetz über den Schutz der Gewässer. (Gewässerschutzgesetz, GSchG). Preprint at https://www.admin.ch/opc/de/classified-compilation/19910022/201701010000/814.20.pdf (2017).

12. Zischg, A. & Riedl, A. *Räumlich-zeitliche Verbreitung der Hagelnetze im Mittleren Etschtal*. (Abenis Alpinexpert GmbH/srl, 2011).

13. Schirpke, U., Meisch, C., Marsoner, T. & Tappeiner, U. Revealing spatial and temporal patterns of outdoor recreation in the European Alps and their surroundings. *Ecosyst. Serv.* **31**, 336–350 (2018).

14. Rüdisser, J., Tasser, E. & Tappeiner, U. Distance to nature—A new biodiversity relevant environmental indicator set at the landscape level. *Ecol. Indic.* **15**, 208–216 (2012).

15. Bundesministerium für Ernährung, Landwirtschaft und Verbraucherschutz. *Statistisches Jahrbuch über Ernährung, Landwirtschaft und Forsten der Bundesrepublik Deutschland. 50. 2006: ...* (Wirtschaftsverl. NW, 2006).

16. Haas, D. Vergiftung von Vögeln durch Pflanzenschutzmittel: Fallbeschreibungen aus Baden-Württemberg und Behandlungs¬möglichkeiten von überlebenden Vögeln. *Ornithol. Jahrb. Baden-Württ.* **3**, 113–120 (1987).

17. Linhart, C. *et al.* Pesticide contamination and associated risk factors at public playgrounds near intensively managed apple and wine orchards. *Environ. Sci. Eur.* **31**, 28 (2019).

18. Grignolio, S., Merli, E., Bongi, P., Ciuti, S. & Apollonio, M. Effects of hunting with hounds on a non-target species living on the edge of a protected area. *Biol. Conserv.* **144**, 641–649 (2011).

19. Gortázar, C., Acevedo, P., Ruiz-Fons, F. & Vicente, J. Disease risks and overabundance of game species. *Eur. J. Wildl. Res.* **52**, 81–87 (2006).

20. Jarnemo, A. & Liberg, O. Red Fox Removal and Roe Deer Fawn Survival: A 14-Year Study. *J. Wildl. Manag.* **69**, 1090–1098 (2005).

21. Ferrari, C., Bogliani, G. & von Hardenberg, A. Alpine marmots ( *Marmota marmota* ) adjust vigilance behaviour according to environmental characteristics of their surrounding. *Ethol. Ecol. Evol.* **21**, 355–364 (2009).

22. Erlinge, S. *et al.* Predation on Brown Hare and Ring-Necked Pheasant Populations in Southern Sweden. *Holarct. Ecol.* **7**, 300–304 (1984).

23. Goszczyński, J. & Wasilewski, M. Studies on the European hare. 46. Predation of foxes on a hare population in central Poland. *Acta Theriol. (Warsz.)* **37**, 329–338 (1992).

24. Angerbjörn, A. Mountain hare populations on islands: effects of predation by red fox. *Oecologia* **81**, 335–340 (1989).

25. Baines, D., Moss, R. & Dugan, D. Capercaillie breeding success in relation to forest habitat and predator abundance: Capercaillie breeding, bilberry and predators. *J. Appl. Ecol.* **41**, 59–71 (2004).

26. Summers, R. W. *et al.* An experimental study of the effects of predation on the breeding productivity of capercaillie and black grouse. *J. Appl. Ecol.* **41**, 513–525 (2004).

27. Cotter, R. C. The Reproductive Biology of Rock Ptarmigan (Lagopus mutus) in the Central Canadian Arctic. *Arctic* **52**, 23–32 (1999).

28. Bernard-Laurent, A., Anceau, C., Faivre, T., Serres, J.-P. & Tangis, S. The reproductive biology of the Rock Partridge *Alectoris graeca saxatilis* in the southern French Alps: first evidence of double-nesting behaviour. *Bird Study* **64**, 513–522 (2017).

29. Tapper, S. C., Potts, G. R. & Brockless, M. H. The Effect of an Experimental Reduction in Predation Pressure on the Breeding Success and Population Density of Grey Partridges Perdix perdix. *J. Appl. Ecol.* **33**, 965–978 (1996).

30. Draycott, R. A. H., Hoodless, A. N., Woodburn, M. I. A. & Sage, R. B. Nest predation of Common Pheasants Phasianus colchicus: Pheasant nest predation. *Ibis* **150**, 37–44 (2008).

31. Sargeant, A. B., Allen, S. H. & Eberhardt, R. T. Red Fox Predation on Breeding Ducks in Midcontinent North America. *Wildl. Monogr.* 3–41 (1984).

32. Tomialojć, L. The Influence of Predators on Breeding Woodpigeons in London Parks. *Bird Study* **25**, 2–10 (1978).

33. Jędrzejewska, B., Jędrzejewski, W., Bunevich, A. N., Miłkowski, L. & Krasiński, Z. A. Factors shaping population densities and increase rates of ungulates in Bialowieza Primeval Forest (Poland and Belarus) in the 19th and 20th centuries. *Acta Theriol. (Warsz.)* **42**, 399–451 (1997).

34. Waite, R. K. Sympatric Corvids: Effects of Social Behaviour, Aggression and Avoidance on Feeding. *Behav. Ecol. Sociobiol.* **15**, 55–59 (1984).

35. Uuemaa, E., Antrop, M., Roosaare, J., Marja, R. & Mander, Ü. Landscape Metrics and Indices: An Overview of Their Use in Landscape Research. *Living Rev. Landsc. Res.* **3**, (2009).

36. Kie, J. G., Bowyer, R. T., Nicholson, M. C., Boroski, B. B. & Loft, E. R. LANDSCAPE HETEROGENEITY AT DIFFERING SCALES: EFFECTS ON SPATIAL DISTRIBUTION OF MULE DEER. *Ecology* **83**, 530–544 (2002).

37. Maier, J. A. *et al.* Distribution and density of moose in relation to landscape characteristics: effects of scale. *Can. J. For. Res.* **35**, 2233–2243 (2005).

38. Dechen Quinn, A. C., Williams, D. M. & Porter, W. F. Landscape structure influences space use by white-tailed deer. *J. Mammal.* **94**, 398–407 (2013).

39. Fearer, T. M. & Stauffer, D. F. Relationship of ruffed grouse *Bonasa umbellus* to landscape characteristics in southwest Virginia, USA. *Wildl. Biol.* **10**, 81–89 (2004).

40. McGarigal, K. & McComb, W. C. Relationships Between Landscape Structure and Breeding Birds in the Oregon Coast Range. *Ecol. Monogr.* **65**, 235–260 (1995).

41. Pattanavibool, A. & Dearden, P. Fragmentation and wildlife in montane evergreen forests, northern Thailand. *Biol. Conserv.* **107**, 155–164 (2002).

42. Lausch, A. & Herzog, F. Applicability of landscape metrics for the monitoring of landscape change: issues of scale, resolution and interpretability. *Ecol. Indic.* **2**, 3–15 (2002).

43. Plexida, S. G., Sfougaris, A. I., Ispikoudis, I. P. & Papanastasis, V. P. Selecting landscape metrics as indicators of spatial heterogeneity—A comparison among Greek landscapes. *Int. J. Appl. Earth Obs. Geoinformation* **26**, 26–35 (2014).

44. Šímová, P. & Gdulová, K. Landscape indices behavior: A review of scale effects. *Appl. Geogr.* **34**, 385–394 (2012).

45. Botequilha Leitão, A. & Ahern, J. Applying landscape ecological concepts and metrics in sustainable landscape planning. *Landsc. Urban Plan.* **59**, 65–93 (2002).

46. Ramesh, T., Kalle, R. & Downs, C. T. Predictors of mammal species richness in KwaZulu-Natal, South Africa. *Ecol. Indic.* **60**, 385–393 (2016).

47. Schindler, S., Poirazidis, K. & Wrbka, T. Towards a core set of landscape metrics for biodiversity assessments: A case study from Dadia National Park, Greece. *Ecol. Indic.* **8**, 502–514 (2008).

48. Tasser, E. *et al.* Long-term game species dynamic as indicator for changing landscape quality. *Sci. Total Environ.* **874**, 162375 (2023).
